# Multiferroic‐Centric Materials and Systems Engineering for Battery Applications: An Insight Into Mechanisms, Strategies, and Characterizations

**DOI:** 10.1002/advs.76021

**Published:** 2026-06-23

**Authors:** Jiaqi Su, Yanda Zhu, Hao Peng, Manman Li, Xuzihan Zhang, Meixiao Wu, Siyuan Zhang, Yuhan Zeng, Jiwen Liao, Ming Luo, Hetaishan Huang, Yutong Wang, Sean Li, Wenxian Li

**Affiliations:** ^1^ School of Materials Science and Engineering The University of New South Wales Sydney NSW 2033 Australia; ^2^ Tera Aurora Electro‐optics Technology Co., Ltd Shanghai China; ^3^ School of Chemical Engineering The University of New South Wales Sydney New South Wales Australia; ^4^ Australian Research Council Centre of Excellence for Carbon Science and Innovation The University of New South Wales Sydney New South Wales Australia

**Keywords:** condensed matter physics, materials science, nanotechnology, system of systems engineering

## Abstract

Ferroic order parameters constitute field‐addressable internal thermodynamic variables that can be switched and coupled to reshape the electrochemical boundary conditions governing batteries. Conventional approaches to engineer battery materials, such as doping, coating, defect, and entropy engineering, primarily alter chemistry and microstructure, and are often static after fabrication. Whereas multiferroicity regulation can act as a complementary design principle that offers internal biases, spin polarization, and reconfigurable strain that dynamically address critical challenges, such as space‐charge formation, sluggish charge‐transfer kinetics, interphase evolution, and dendrite‐assisted failure, found in battery systems. This review dissects ferroic‐driven electrochemical performance enhancements from the cell level down to individual components and buried interfaces, establishing a mechanistic framework that correlates single‐ and coupled‐ferroic responses to measurable electrochemical descriptors. Beyond mechanistic analysis, we assess strategies such as material architecture design and field engineering that enable deterministic control of targeted ferroic orders across diverse battery chemistries. Further, we consolidate ferroic‐resolved characterizations, including advanced magnetometry, spectroscopic and microscopic techniques, as well as the emerging *operando* and in situ setups that are critical to battery systems. Overall, this work bridges ferroic‐mediated electrochemistry and multiferroic‐centric battery engineering, enabling next‐generation battery systems in which ferroic order parameters operate as dynamic, *operando*‐addressable state variables under realistic cycling conditions.

## Graphical abstract

Multiferroic order parameters – polarization, magnetization, and ferroelastic strain – are positioned as dynamic design variables for batteries. Their mechanistic roles, practical tuning through fabrication and external fields, and ferroic‐resolved characterization routes are unified into a closed‐loop framework, revealing how coupled ferroic responses can regulate transport, interfacial reactions, and stability across next‐generation battery chemistries.

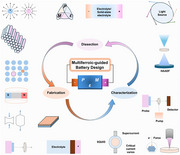

**Abbreviations**
ASR: Area‐specific resistance
ASSB: All‐solid‐state battery
AFMR: Antiferromagnetic resonance
AFM: Atomic force microscopy
BEF: Built‐in electric field
BCDI: Bragg coherent diffraction imaging
BE‐ESM: Band‐excitation ESM
CCD: Critical current density
CEI: Cathode/electrolyte interphase
CBM: Conduction band minimum
DM: Dzyaloshinskii‐Moriya
DE: Double‐exchange
DPC: Differential phase contrast
ESM: Electrochemical strain microscopy
EXAFS: Extended X‐ray absorption fine structure
*E*_F_
: Fermi level
HRTEM: High‐resolution transmission electron microscopy
IETS: Inelastic electron tunneling spectroscopy
Li–S: Lithium–Sulfur
LPS: Lithium polysulfide
LDH: Layered double hydroxide
MOFs: Metal organic frameworks
MHD: Magnetohydrodynamic
NR: Neutron reflectometry
NV: Nitrogen‐vacancy
NMR: Nuclear magnetic resonance
OER: Oxygen evolution reaction
PLM: Polarized light microscopy
PVDF: Polyvinylidene fluoride
pDOS: Partial density of states
PDS: Potential‐determining step
PNR: Polarized neutron reflectometry
PFM: Piezoresponse force microscopy
RDS: Rate‐determining step
RSXS: Resonant soft X‐ray scattering
SEI: Solid‐electrolyte interphase
SEM: Scanning electron microscope
STEM: Scanning transmission electron microscope
SPM: Scanning probe microscopy
SQUID: uperconducting quantum interference device
SHG: Second‐harmonic generation
SP‐STM: Spin‐polarized scanning tunneling microscopy
TMO: Transition‐metal oxide
TR‐MOKE: Time‐resolved magneto‐optical Kerr effect
TR‐XRD: Time‐resolved XX‐ray diffraction
THz‐TDS: THz time‐domain spectroscopy
VBM: Valence band maximum
VSM: Vibrating sample magnetometry
XRD: X‐ray diffraction
XAS: X‐ray absorption spectroscopy
XPS: X‐ray photoelectron spectroscopy
XFELs: X‐ray free‐electron lasers
ZIBs: Zinc‐ion batteries

## 1 Introduction

**FIGURE 1:**
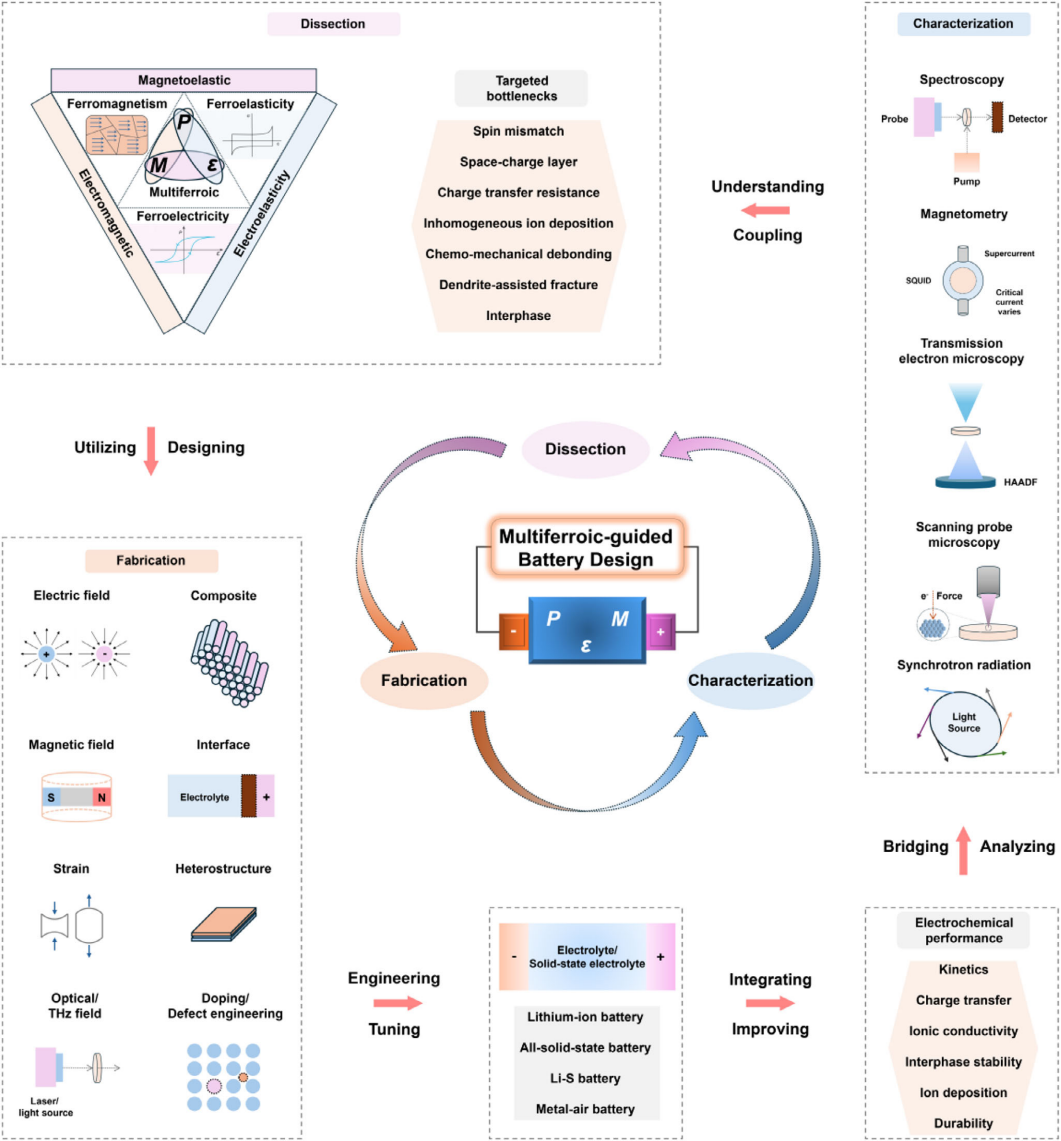
Unconventional multiferroic‐centric battery design framework. Schematic overview of the logic of this review. The top‐left panel summarizes the dissection of ferroic orders – polarization, magnetization, and ferroelastic strain – and their electromagnetic, magnetoelastic, and electromechanical couplings, which are analyzed against the key battery bottlenecks they can address. The left panel outlines representative fabrication and tuning routes, by which ferroic responses can be introduced into battery‐relevant materials. The right panel summarizes the corresponding characterizations for resolving ferroic order under working conditions. These three directions – dissection, fabrication, and characterization – form a closed‐loop framework (center) for establishing causal links between ferroic state variables and electrochemical function. The bottom panel highlights the target battery platforms (lithium‐ion, all‐solid‐state, Li–S, and metal‐air batteries) and the resulting performance descriptors, including kinetics, charge transfer, ionic conductivity, interphase stability, ion deposition behavior, and durability. Overall, this figure illustrates how multiferroicity can be translated from a set of coupled order parameters into a practical design principle for next‐generation battery materials and interfaces.

Multiferroic materials, exhibiting spontaneous polarization (ferroelectricity), magnetization (ferromagnetism), or strain variants (ferroelasticity), provide field‐addressable internal biases, spin textures, and reconfigurable strain fields that couple directly to charge and ion transport [1, 2]. Leveraging these order parameters for electrochemistry is emerging as a distinct, multidisciplinary route to advance next‐generation batteries, offering levers that conventional lewis‐acid/base catalysis, porosity/texture control, and defect/dopant chemistry cannot supply alone. Across battery chemistries, performance remains limited by buried solid‐solid space‐charge barriers and charge‐injection resistance [3]; spin‐mismatched multi‐electron catalysis in ORR/OER and chalcogen redox [4]; nonuniform metal plating with dendrite‐assisted fracture [5]; and chemo‐mechanical debonding that escalates area‐specific resistance (ASR) and undermines critical current density (CCD) [6]. Multiferroic tuning targets these bottlenecks mechanistically (Figure 1): (i) ferroelectric polarization and flexo‐fields collapse space‐charge layers, lower charge‐transfer resistance (*R*_ct_), create domain‐wall ion highways, and steer ion deposition [1, 7, 8, 9]; (ii) ferromagnetic order injects spin‐polarized carriers to enforce spin‐selection rules and strengthen *d*‐*p* hybridization [10], while modest magnetic fields homogenize magneto‐ionic flux; and (iii) ferroelastic twin architectures supply pseudo‐plastic compliance, crack deflection, and strain‐gradient potentials that stabilize interphases and sustain high‐rate cycling. Together, these couplings reduce overpotentials, raise CCD, and extend durability in diverse battery systems. These approaches collectively serve as a complementary lever to conventional battery material design principles for further advancements.

Although researchers across fields recently started to utilize multiferroic‐induced effects in electrochemistry, limitations such as ambiguous attribution to certain ferroic order [11], insufficient control and retention of ferroic properties, and lack of real‐time/in *operando* analyzing techniques are placing multiferroicity as a tunable yet often neglected variable in battery materials. These issues are owing to mechanistic and methodological gaps. The contributions of ferroelectric, ferromagnetic, and ferroelastic orders are often ambiguously assigned and neglected, impeding rational tuning and cross‐platform transferability, and restricting rational material design and fabrication. Beyond materials synthesis, the field lacks an established, battery‐relevant characterization framework to quantify polarization (*P*) [12], magnetization (*M*) [13], and ferroelastic strain (*ε*) [14] in situ/*operando* and to causally link them to electrochemical descriptors such as Rct/ASR, diffusion coefficients, interphase evolution, and fracture metrics. Conventional X‐ray diffraction (XRD)/X‐ray absorption spectroscopy (XAS)/X‐ray photoelectron spectroscopy (XPS)/Raman spectra reveal composition and average structure but rarely resolve ferroic‐mediated dynamics of the material itself or across battery components with temporal and spatial fidelity. Eventually, a vicious cycle formed: scientifically less reliable or valid analysis blankets mechanistic insights, which in turn mislead materials design, and divert the research trajectories away from predictive frameworks anchored in measurable descriptors to passive characteristic assignments.

This review aims to break the cycle and addresses the gaps by (i) dissecting multiferroicity into its ferroelectric [15], ferromagnetic [16], and ferroelastic components explicitly within an electrochemical context; (ii) mapping how each order parameter modulates charge injection, ion transport, interfacial catalysis, and fracture; and (iii) consolidating state‐of‐the‐art tuning strategies including multiphysics fields, architectural design, and ferroic‐centric defect/coordination engineering for battery materials (Figure 1). Complementing design, we establish an overarching characterization framework for batteries that integrates magnetometry, scanning probe microscopies, polarized light microscopy (PLM), ultrafast optical/X‐ray probes, synchrotron/neutron methods (including in situ and *operando* setups), and correlative electrochemistry to quantitatively link *P*/*M*/*ε* fields to performance metrics in real time. Overall, this article, to our knowledge, is the first comprehensive review that unifies multiferroic mechanisms, tuning schemes, and *operando* probes into a coherent roadmap purpose‐built for rechargeable batteries – moving beyond analogies from conventional electronics to battery‐specific constraints. We emphasize system‐matched validation and standardized descriptors that translate order parameters into electrochemical observables, enabling predictive comparisons across conventional liquid‐state battery, halide/oxide/sulfide all‐solid‐state battery (ASSB) [17], Li–Sulfur (Li–S) [18], and metal‐air systems [19]. We concluded this review by outlining opportunities where multiferroic‐driven design can unlock orthogonal performance gains – space‐charge elimination and dipole‐mediated catalysis at cathodes, spin‐selective pathways for multi‐electron reactions, ferroelastic toughening in solid electrolytes, and field‐programmable interphases – thereby advancing next‐generation battery systems while opening a new, rigorously mechanistic application frontier for multiferroics.

## 2 Dissecting Multiferroic Properties for Battery Materials Design

Multiferroics are single‐phase solids in which two or more primary ferroic order parameters coexist within the same thermodynamic phase [20]. Each ferroic order represents a collective ground state arising from the spontaneous breaking of a fundamental symmetry: ferroelectricity from inversion symmetry, magnetism from time‐reversal symmetry [21], and ferroelasticity from the rotational symmetry of the crystal lattice [20, 22, 23]. The simultaneous violation of these symmetries enables multiferroic coupling, which is rooted in the microscopic interplay of spin, orbital, and lattice degrees of freedom mediated by spin‐orbit coupling [24]. Understanding ferroic‐originated electrochemical enhancement is essential because ferroic order reshapes the interfacial electronic landscape, such as band bending [25], spin‐resolved density of states [26], and strain‐tuned orbital overlap, thereby directly governing charge‐transfer kinetics at cathode/anode and interphase boundaries [27, 28, 29]. These charge‐transfer boundary conditions subsequently manifest as transport‐limited battery metrics. Accordingly, we summarize key studies and critically extract mechanistic insights that link ferroic order parameters to charge‐transfer descriptors and cell‐level performance.

### 2.1 Background

#### 2.1.1 Previous Reviews

Several focused reviews have already established the importance of ferroelectric or dielectric polarization in selected battery problems. Li et al. summarized ferroelectric materials for high‐energy batteries from the perspective of spontaneous polarization–assisted ion transport across anodes, cathodes, and electrolytes, emphasizing fundamentals, material classes, and ferroelectric strategies for improving transport kinetics [30]. Zhang et al. further narrowed the discussion to ferro‐/piezoelectric polarization for dendrite‐free metal anodes, highlighting reduced local current density, homogenized ion flux, and stress tolerance during plating/stripping [31]. Wang et al. then expanded the dielectric viewpoint to solid‐state batteries, reviewing piezoelectric, ferroelectric, pyroelectric, and related functional dielectrics as active regulators of space‐charge layers, cation ordering, and interfacial transport in solid electrolytes and electrodes [32]. More recently, Wang et al. focused on ferroelectric‐enhanced rapid charging and long‐term stability, and importantly stressed that improvements are not always uniquely attributable to ferroelectricity, but may also arise from competing dielectric, mechanical, or interfacial effects, thereby identifying a persistent attribution problem in the field [33]. In parallel, Wu et al. reviewed ferromagnetic metal sulfides/selenides as anode materials for Na‐ and K‐ion batteries, but from a materials‐class perspective centered on synthesis, structure, storage mechanism, and electrochemical properties, rather than on magnetic order as a controllable electrochemical variable [34]. Taken together, these studies have substantially advanced the field, yet they remain largely single‐ferroic, component‐specific, or materials‐family‐based in scope. Our review aims to precisely fill these gaps and expand the scope for future research, moving beyond conventional ferroelectric‐centered narratives by systematically dissecting ferroelectric, ferromagnetic, and ferroelastic orders, as well as their couplings, in battery‐centric contexts. Also, we extend the discussion to unconventional and coupled multiferroic systems, and correlate them with measurable electrochemical descriptors.

#### 2.1.2 Fundamental Concepts of Multiferroicity

##### 2.1.2.1 Ferroelectricity

A ferroic order parameter is the primary thermodynamic variable that defines a ferroic state and can be written, switched, and retained under an external stimulus [20, 35]. Ferroelectricity arises from inversion‐symmetry breaking and produces a spontaneous polarization that is reversible under an electric field; its electrochemical significance lies in the built‐in electric fields generated at polarized surfaces and interfaces, which modulate band bending, space‐charge formation, ion distribution, and adsorption energetics [30]. In this context, spontaneous polarization refers to the intrinsic dipole ordering in zero field, whereas remanent polarization is the fraction retained after the field is removed, and thus determines whether a ferroic interface can sustain a persistent electrostatic bias under practical battery conditions [36].

##### 2.1.2.2 Ferromagnetism

Ferromagnetism, more broadly magnetic ordering, denotes the spontaneous alignment of magnetic moments below a characteristic ordering temperature [37, 38, 39, 40, 41]. Beyond static magnetization, it controls spin polarization, exchange interactions, and the symmetry of electronic states near the Fermi level, thereby modifying interfacial charge‐transfer pathways, especially for multielectron reactions involving open‐shell intermediates.

##### 2.1.2.3 Ferroelasticity

Ferroelasticity denotes the existence of symmetry‐equivalent spontaneous strain states that can be switched by stress, giving rise to reorientable structural variants [42]. Unlike ordinary elastic deformation, ferroelastic switching involves crystallographic reconfiguration and domain rearrangement, making it highly relevant to batteries where repeated lithiation, sodiation, conversion, or oxygen redox generates large internal stresses. A defining feature of ferroics is the formation of domains and domain walls: domains are regions in which the order parameter adopts a uniform orientation or variant, whereas domain walls are the interfaces between neighboring domains [43].

##### 2.1.2.4 Multiferroic Coupling

When different ferroic orders interact, the resulting cross‐response is termed multiferroic coupling [20], and specifically magnetoelectric coupling when polarization and magnetization are linked; this coupling is attractive because a single external stimulus can reshape multiple internal state variables simultaneously [44]. Finally, three electromechanical effects are particularly important for battery interfaces: piezoelectricity, the linear coupling between strain and polarization in non‐centrosymmetric solids [45]; flexoelectricity, polarization induced by strain gradients and therefore amplified at nanoscale interfaces, defects, and inhomogeneously stressed regions [46]; and chemopiezoelectricity, in which defect‐ or composition‐coupled strain, often associated with mobile vacancies or local stoichiometric gradients, generates electrically active deformation fields.

#### 2.1.3 Fundamental Concepts of Battery Electrochemistry

In battery systems, a space‐charge layer is an interfacial region of ion accumulation or depletion that forms when two phases with different chemical potentials or defect chemistries come into contact [3]. As the layer perturbs local carrier concentration and electric field, it can severely restrict ion transport across buried interfaces, especially in solid‐state batteries. Charge‐transfer resistance refers to the kinetic barrier for interfacial electrochemical reactions, that is, the difficulty of transferring charge across an electrode/electrolyte boundary during redox [47]. Area‐specific resistance (ASR) is the total resistance normalized to electrode area and is widely used as a practical descriptor of interfacial and bulk transport losses in real cells. Critical current density (CCD) is the maximum current density a system can sustain before unstable deposition, filament growth, shorting, or dendrite penetration occurs, making it a key metric for metal‐anode and solid‐state battery stability [48]. There are two fundamental transport descriptors, where ionic conductivity measures the ability of ions to move through an electrolyte or active material under an electric field [49], and the diffusion coefficient quantifies how rapidly ions redistribute in response to concentration gradients [50]. Together they define the intrinsic and effective transport capability of electrodes, electrolytes, and interfaces. Overpotential is the voltage penalty beyond thermodynamic equilibrium required to drive a given current, and reflects kinetic, ohmic, and mass‐transport limitations [51]. At buried interfaces, chemically and mechanically evolving surface layers are commonly described as the solid electrolyte interphase (SEI) on anodes [52], the cathode electrolyte interphase (CEI) on cathodes, or more generally the interphase; these layers often determine interfacial stability, ion selectivity, and long‐term resistance growth [53]. In metal‐anode systems, plating/stripping denotes the repeated deposition and dissolution of metallic species such as Li, Na, or Zn, while dendrite nucleation describes the onset of highly localized, unstable growth morphologies that can amplify current constriction, fracture interfaces, and trigger failure [54]. For cathodes and electrocatalytic interfaces, multielectron redox kinetics – including oxygen reduction/evolution (ORR/OER) and chalcogen redox – describe reactions involving multiple coupled electron‐transfer steps and reactive intermediates, whose energetics can be highly sensitive to interfacial electric fields, spin polarization, and adsorption symmetry [55]. Finally, chemo‐mechanical degradation refers to the coupled evolution of chemical instability and mechanical damage during cycling, including crack formation, particle pulverization, contact loss, and stress‐driven interfacial debonding; this is especially critical in all‐solid‐state batteries, where transport continuity depends directly on maintaining intimate solid‐solid contact.

### 2.2 Ferroelectric and Polarization‐Mediated Regulation of Battery Interfaces

Ferroelectric and polarization‐active materials regulate battery interfaces primarily by generating built‐in electric fields, surface‐bound charges, and local polar environments that couple to ion redistribution, charge transfer, defect migration, and interfacial reaction kinetics. These polarization effects can originate from coherent ionic displacement or electronic charge‐density reorganization, including lone‐pair‐driven polar distortions in cations with stereochemically active *ns* ^2^ electron configurations, geometric lattice instabilities, charge ordering, and spin‐driven symmetry breaking [22, 23, 56, 57], as well as defect‐ and interface‐induced local dipoles. Although these mechanisms differ in crystallographic and electronic origin, their electrochemical relevance lies in their ability to impose switchable or persistent electrostatic boundary conditions at electrode/electrolyte interfaces. Such boundary conditions can mitigate space‐charge accumulation, homogenize metal‐ion flux, promote directional ion migration along domain walls or polar interfaces, stabilize redox intermediates, and tune adsorption or charge‐transfer energetics. This section therefore focuses not on unconventional ferroelectrics as a separate materials class, but on how ferroelectric polarization and broader polarization‐mediated effects are exploited to regulate battery interfaces, including interfacial charge transfer, ion migration, metal deposition, dendrite suppression, and polarization‐modulated interfacial electrochemistry. Where ferroelectric switching has not been rigorously established, the relevant effects are described more cautiously as polarization‐mediated, dielectric‐field, defect‐mediated, or interfacial field‐redistribution phenomena.

#### 2.2.1 Interfacial Charge Transfer

**FIGURE 2:**
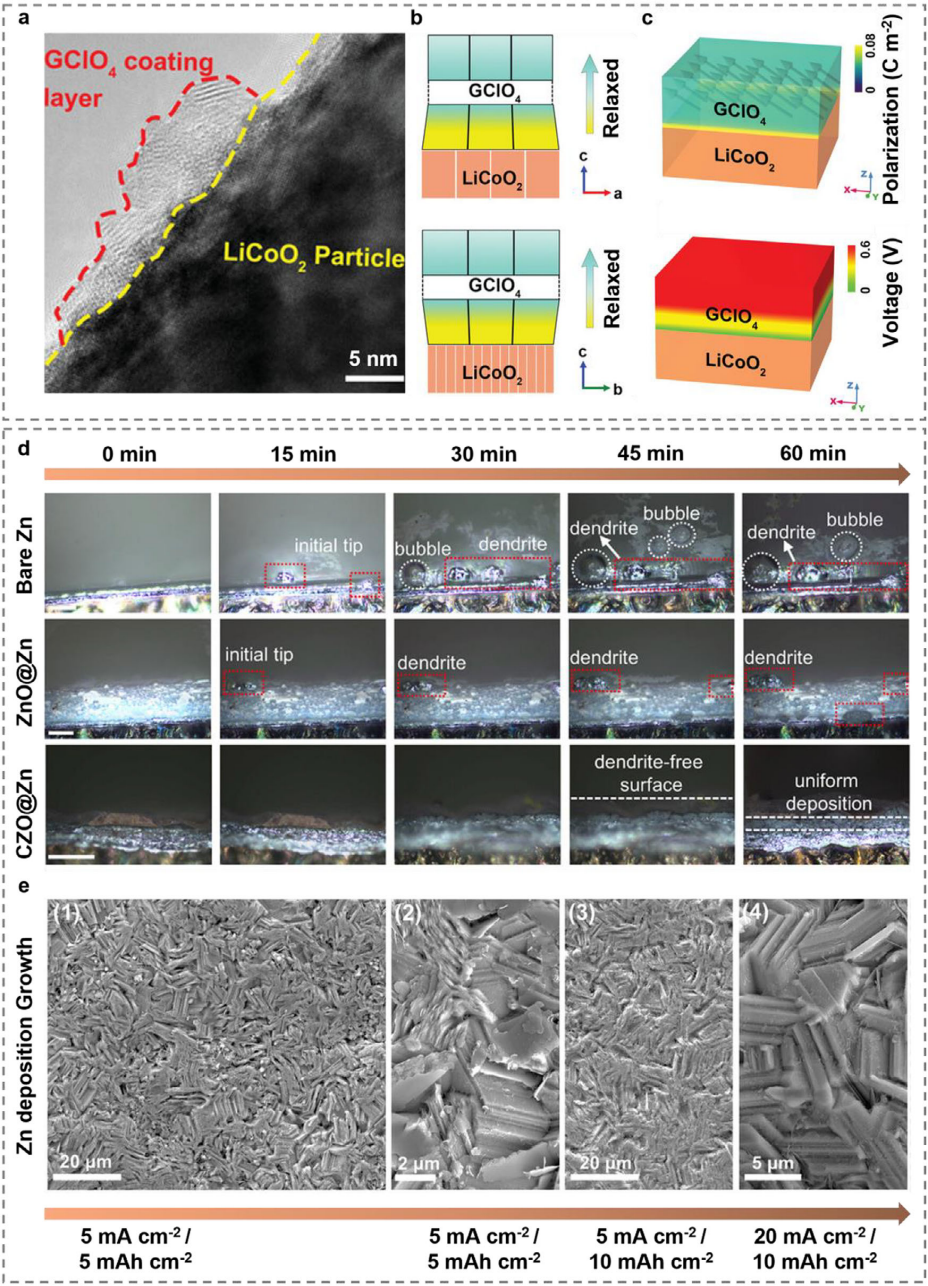
Ferroelectric coatings create built‐in electric fields at battery interfaces, which suppress space‐charge accumulation and guide more uniform Zn deposition, thereby providing a direct interfacial route to lower resistance and mitigating dendrite growth. Direct observations and mechanistic insights into ferroelectric built‐in fields and space‐charge layer modulations. (a–c) Ferroelectric built‐in electric field construction mechanism. Reproduced with permission [60]. Copyright 2023, John Wiley and Sons. (a) High‐resolution transmission electron microscopy (HRTEM) picture of GClO_4_‐coated LiCoO_2_ cathode particle. (b) Schematic diagram of cell distortion of GClO_4_ coatings, where GClO_4_ is stretched along *a* direction and compressed along *b* direction. (c) The phase‐field simulation verifies the orientation of dipoles in the ferroelectric coating and the generation of built‐in electric field. (d, e) Polarization‐guided Zn deposition. Reproduced with permission [64]. Copyright 2025, John Wiley and Sons. (d) in situ optical microscope observation of Zn plating behavior of bare Zn, ZnO@Zn, and CZO@Zn anode under 10 mA cm^−2^ (scale bar: 100 µm). (e) Scanning electron microscope (SEM) visualization of Zn deposition growth underneath the CZO layer.

Ferroelectric materials can create built‐in electric fields at battery materials’ interfaces, counteracting space‐charge barriers and facilitating ion transport. In ASSBs, ionic discontinuities at electrode/electrolyte contacts lead to space‐charge regions such as ion accumulation/depletion layers that raise interfacial resistance [58]. Ferroelectric polarization can intrinsically mitigate this problem by producing an internal electric field that drives mobile ions across the interface, suppressing space‐charge formation [59, 60, 61]. Figure 2a shows a LiCoO_2_ cathode coated with a ferroelectric guanidinium perchlorate (GClO_4_) layer, and its cell achieved ∼91.6% of the liquid‐cell capacity (≈210 mAh g^−1^) due to an “upward” self‐polarization at the interface [60]. Here, the flexoelectric effect reduces the degree of lattice distortion/mismatch with increased molecular layers (Figure 2b), which effectively guides the ferroelectric domain to uniformly nucleate and induces the single‐domain polarization state. This phenomenon is validated with phase‐field simulations – ferroelectric dipoles induced *Z‐*direction polarization and voltage differences (Figure 2c), resulting in a downward field pulling Li^+^ toward the cathode‐electrolyte junction and markedly reducing the space‐charge layer impedance [60]. Similarly, a thin interlayer of K_0.5_Na_0.5_NbO_3_ (KNN) ferroelectric was used in a Na^+^‐metal ASSB to build a local field that aligns KNN's dipole during charging, while neutralizing the interfacial potential drop and attenuating Na^+^ accumulation. This improved interfacial Na^+^ conduction, enabling an unusually high discharge capacity of ∼160 mAh g^−1^ with ∼97% retention after 165 cycles [61]. These studies highlight that orienting ferroelectric dipoles favorably at electrode contacts can eliminate interfacial ion pile‐up to facilitate fast charge transfer [62, 63].

Beyond solid interfaces, polarization‐derived or dielectric‐field effects may also benefit metal‐anode deposition at solid–liquid interfaces by homogenizing local cation flux during plating. Recently, a composite interlayer of Cr‐doped ZnO and poly(vinylidene fluoride) (PVDF) was introduced on zinc metal, and the resulting CZO@Zn layer was reported to promote a more uniform electric‐field distribution across the anode (Figure 2d), yielding a dense “concrete‐slab” Zn deposition morphology rather than spiky dendrites (Figure 2e), together with high reversibility and extended cycling stability [64]. Zinc ions were induced to deposit epitaxially as a dense film, yielding near 99.97% reversibility and ultra‐long stability (over 2400 h) even at high rates [64]. However, attributing these improvements specifically to ferroelectric polarization still requires more rigorous validation, because the reported polarization‐electric field loops are unsaturated and lossy, features that may also be consistent with leakage‐related dielectric behavior rather than unambiguous ferroelectric switching. Accordingly, the improved Zn deposition is more cautiously interpreted as arising from a combination of interfacial field redistribution, dielectric response, defect‐mediated local polarization, and modified nucleation kinetics, rather than from definitively established ferroelectricity alone. The specific role of ferroelectric polarization in regulating deposition behavior therefore, warrants further investigation, particularly through more *operando* studies; this issue is discussed in greater detail later in this review.

In Li metal cells, ferroelectric polymer coatings have been shown to carry a negatively charged surface that guides Li^+^ plating more uniformly by encouraging lateral Li^+^ diffusion along the interface [60, 65]. Overall, the internal electric field of ferroelectrics serves as a self‐regulating bias, redistributing ions in a way that avoids local depletion or excess [59]. This not only accelerates ion transfer kinetics but also prevents interfacial instabilities such as dendrites or high interfacial resistance. The key requirement is that the ferroelectric dipoles be coherently oriented in the direction beneficial for ion transport; randomly oriented or cancelling dipoles provide no net field [62]. Consequently, achieving a well‐poled ferroelectric state at critical interfaces is a primary design aim to leverage built‐in fields for improved battery performance [60, 63].

#### 2.2.2 Ion Migration

**FIGURE 3:**
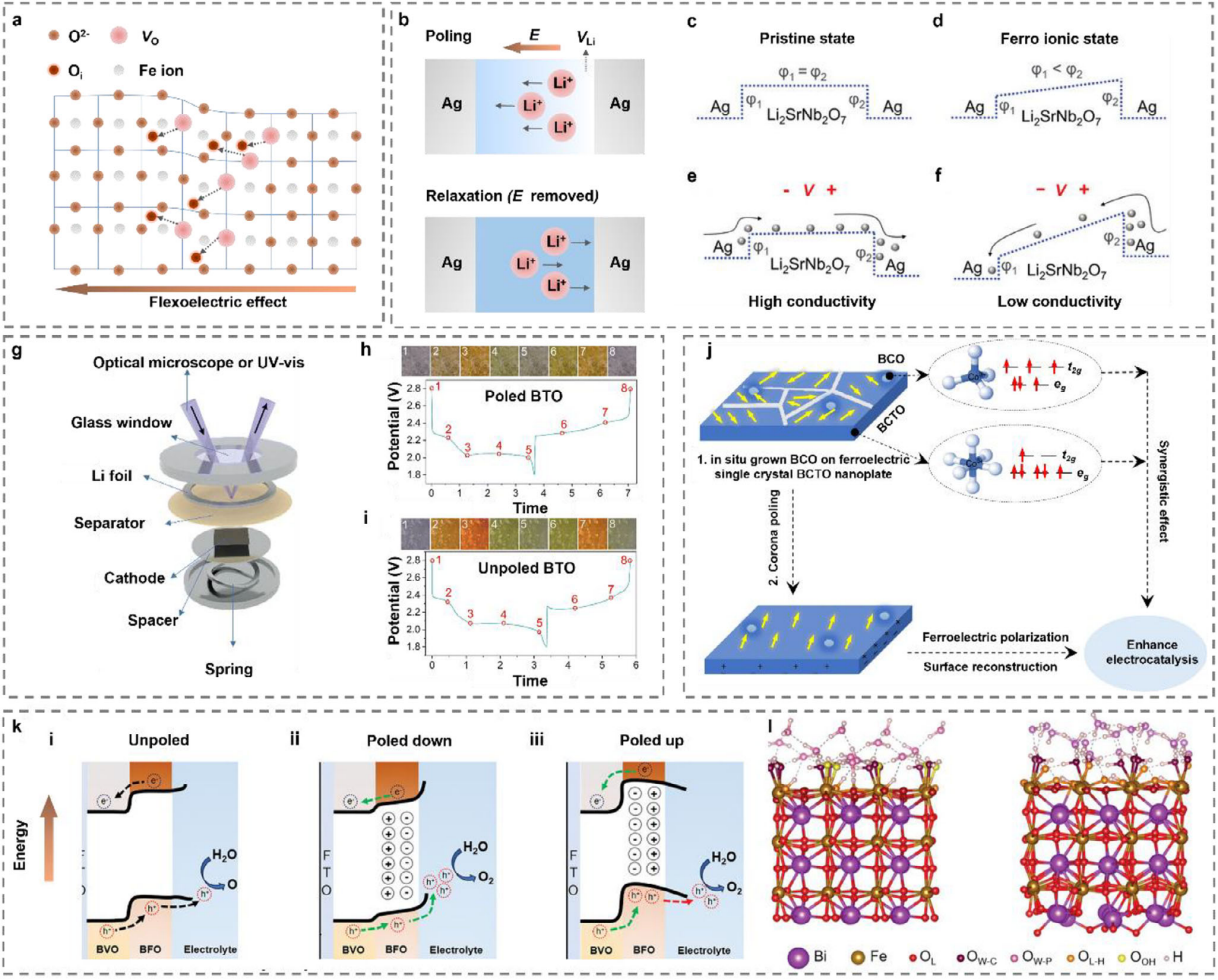
Ferroelectric polarization can actively direct ion migration, lower interfacial barriers, suppress polysulfide shuttling, and tune catalytic/charge‐transfer pathways, demonstrating that built‐in polar fields can be used to control both transport and reaction chemistry across battery and related electrochemical interfaces. (a) Schematic of anti‐Frenkel defect formation and alignment in a flexoelectric field arising from the large strain gradient at the MPB: an oxygen vacancy‐interstitial pair forms a defect dipole that couples to the flexoelectric field. Reproduced with permission [70]. Copyright 2024, John Wiley and Sons. (b–f) Field‐controlled Li‐ion migration, interfacial barriers, and rectification in the ferroionic state. Reproduced with permission [71]. Copyright 2022, John Wiley and Sons. (b) Schematic of Li^+^ migration at room temperature with and without an external electric field. Qualitative schematics of (c) the interfacial Schottky barriers in the pristine state and (d) in the ferroionic state after field cooling. Schematics of (e) the low‐conductivity and (f) high‐conductivity directions. (g–k) *Operando* UV–vis DRS and optical microscopy of Li–S cell with BaTiO_3_ cathodes. Reproduced with permission [74]. Copyright 2024, The Royal Society of Chemistry. (g) Schematic configuration of the *operando* Li–S coin cell; Evolution of polysulfide concentration at the separator during galvanostatic cycling, determined by UV–vis, for cells with the first‐cycle galvanostatic profiles at C/6 for (h) poled BTO and (i) unpoled BTO. (j) Schematic illustration of the electrocatalytic advantages of in situ‐prepared BCO/BCTO nanostructures. In the in situ grown BCO, Co ions adopt tetrahedral coordination with an (*e*_g_)^3^(*t*_2g_)^3^ configuration, whereas in ferroelectric BCTO, Co ions are octahedrally coordinated with a (*t*_2g_)^5^(*e*_g_)^1^ configuration; the coexistence of these distinct Co environments synergistically enhances OER activity. Corona poling further improves OER performance by activating ferroelectric polarization and inducing surface modification. Reproduced with permission [78]. Copyright 2019, Springer Nature. (k, l) Polarization‐controlled band engineering and interfacial chemistry in BFO/BVO. Reproduced with permission [79]. Copyright 2025, John Wiley and Sons. (k) Schematics of (i) unpoled, (ii) down‐poled, and (iii) up‐poled BFO/BVO, showing poling‐induced band shifts and band‐energy gradients within the BFO layer and their impact on charge transport; arrow colors denote relative charge‐transfer rates, with green and red indicating faster and slower transfer, respectively, compared to the unpoled benchmark (black). (l) Interfacial oxygen‐containing species at the BFO/water interface for up‐poled (left) and down‐poled (right) BFO.

Ferroelectric materials can also enhance ionic conductivity by offering highly conductive pathways at domain boundaries and through defect coupling [66]. Ferroelectric domain walls – the nanoscale boundaries between regions of different polarization orientation – often host an accumulation of charged defects that render them more conductive than the bulk domains [8, 66, 67, 68]. In perovskite oxides, oxygen vacancies tend to migrate into domain walls under the internal field of polarization [68], effectively creating conductive channels or 2D “highway” networks for ionic movement [8, 67, 69]. In La‐doped BiFeO_3_ thin films, highly distorted tetragonal‐like phases exhibit “phase boundary conduits” where an aligned array of anti‐Frenkel defect dipoles (anion vacancy‐interstitial pairs) forms along the domain interfaces (Figure 3a). These defect dipoles, aligned by the flexoelectric effect at a strained morphotropic phase boundary, give rise to a metastable interfacial ferroelectric phase with enhanced ionic conductivity (Warburg‐type impedance response) [67, 70]. In essence, the ferroelectric domain structure can spatially modulate ionic defect distributions, concentrating charge carriers in specific planes and thereby lowering ionic transport barriers [67, 70].

Ferroelectric order in Li_2_SrNb_2_O_7_ demonstrates direct interplay between polarization and ionic conduction [71]. Upon electric poling, Li^+^ vacancies in Li_2_SrNb_2_O_7_ were observed to drift and accumulate at one crystal face, effectively forming a space‐charge layer that dramatically increased in‐plane conductivity (Figure 3b). The result was a giant memristive switching, where DC conductivity rose by three orders of magnitude (to ∼2.7 × 10^−3^ S·m^−1^ at room temperature) after poling, and a rectifying junction behavior emerged due to the asymmetric Li^+^ distribution (Figure 3c–f) [71]. This behavior manifests that moving ions can switch or pin polarization, and vice versa, suggesting embedding ferroelectric regions in solid electrolytes or electrodes could create self‐adjusting ionic pathways [65]. Even though ferroelectric crystals themselves are electronic insulators, the local current active regions around polarized particles or domain walls may facilitate Li^+^ percolation across an otherwise ion‐blocking matrix [60]. For example, ferroelectric ceramic fillers (BaTiO_3_, LiTaO_3_, etc.) in a PVDF‐based composite solid electrolyte were shown to repress interfacial space‐charge and improve overall Li^+^ conductivity, achieving ∼6.5 × 10^−4^ S·cm^−1^ at room temperature [65]. The BaTiO_3_ nanofibers in that composite established a long‐range polar interface in the polymer, while also providing surface Li_2_Se regions (via a MoSe_2_ coating) that acted as fast ion‐conduction channels. In tandem, the piezoelectric nature of the polarized fibers could dynamically suppress dendrite formation by locally reversing charge build‐up under stress [65].

Beyond domain‐wall‐mediated conduction, recent studies further show that ferroelectric or polar‐lattice effects can enhance ion migration by directly reconfiguring the local transport environment at electrolyte/electrode interfaces or within defect‐sensitive perovskite frameworks. In aqueous Zn batteries, ferroelectric molecules partially replace H_2_O in the Zn^2+^ solvation sheath and enrich the inner Helmholtz plane, thereby suppressing free‐water activity, homogenizing Zn^2+^ flux, lowering the Zn nucleation overpotential, and mitigating dendrite‐promoting ion accumulation during plating/stripping [72]. Complementarily, in NaNbO_3_‐based non‐stoichiometric ion conductors [73], varying Na stoichiometry changes the dominant defect population from vacancies to interstitials, while concurrently driving NbO_6_ octahedra from compressed toward flattened/twisted configurations; these coupled defect‐structural distortions selectively expand the Na‐O‐Na and Na‐O‐Nb bottlenecks for Na^+^ transport and promote O^2−^ migration through octahedral networks. These results extend the concept of ferroelectric‐assisted ion transport beyond classical ferroelectric domain walls, indicating that local polar fields, defect chemistry, and distortion‐sensitive polyhedral frameworks can all serve as effective design handles for directing ionic pathways and improving electrochemical transport.

In summary, ferroelectric domain engineering together with defect modulation leverage domain walls as conduits and uses the internal field to steer defect distribution, enhancing ionic conductivity beyond what is possible in non‐ferroic lattices. Importantly, these effects are highly anisotropic and local – confined to the vicinity of domains or filler‐matrix interfaces – underscoring the need to design microstructures that maximize the connectivity of these fast pathways throughout the electrode or electrolyte [8].

#### 2.2.3 Polarization‐Mediated Interfacial Electrochemistry

A defining electrochemical feature of ferroelectrics is their ability to generate polarization‐derived interfacial electric fields and surface charges, which can profoundly influence local surface chemistry by attracting, repelling, or redistributing ionic species [8, 62, 74]. Ferroelectric regulation of electrochemical performance, however, is not restricted to classical surface‐charge‐mediated adsorption. A recent NaNbO_3_‐based lead‐free dielectric study shows that the functional output of a polar material can instead emerge from hierarchical polar‐order engineering, spanning sub‐angstrom electron‐cloud deformation, B‐site displacement, polar nano‐region assembly, and electrical microstructure reconfiguration [75]. In this system, electron‐cloud deformation strengthens Nb‐O dipole ionicity, while highly reversible polar nano‐regions delay polarization saturation and suppress remanent loss, demonstrating that local electronic polarization and nanoscale polar disorder can govern charge‐storage behavior even in the absence of a simple uniformly polarized ferroelectric state. This insight broadens the electrochemical role of ferroelectrics: beyond serving as charged surfaces for adsorbate binding, they can also engineer the local electrostatic landscape that precedes and conditions interfacial reaction pathways. This property has been harnessed to improve adsorption of reactants and intermediates in various battery chemistries, thereby enhancing reaction kinetics and cyclability [8, 62].

Ferroelectric additives mitigate the polysulfide shuttle in Li–S batteries because their remanent polarization generates a persistent internal electric field and surface‐bound charges that impose an electrostatic barrier against anion migration. This polarization‐derived field both strengthens the local confinement of soluble Li_2_S_x_ intermediates and suppresses their diffusion toward the Li‐metal anode, thereby reducing parasitic redox reactions, self‐discharge, and capacity decay. Ferroelectric BaTiO_3_ particles with aligned dipoles exhibit strong Coulombic interactions with polysulfide anions, enabling electrostatic anchoring and spatial localization of Li_2_S_x_ species within the cathode region [74]. An *operando* study of a Li–S cathode (Figure 3g) directly confirmed that ferroelectric dipole alignment suppresses polysulfide concentration in the electrolyte by strong Coulombic adsorption on the BTO surface (Figure 3h,i), where the poled BTO created a high surface potential that anchored polysulfide species. Crucially, high overall dipole alignment correlates with increased surface charge and thus stronger polysulfide affinity. Early proof‐of‐concept work showed that simply mixing ferroelectric BTO into a sulfur cathode could already improve polysulfide retention, attributed to its spontaneous polarization, which created an internal field anchoring “heteropolar” polysulfides [76]. In essence, ferroelectrics in separators or as coatings have been shown to both physically confine polysulfides and catalytically accelerate their conversion to insoluble phases [77]. The polarized surfaces can function like Lewis's acid‐base adsorption sites and simultaneously provide an electric field that lowers activation energy for polysulfide redox reactions.

Ferroelectric polarization has analogous benefits in other electrochemical reactions, such as the electrocatalytic oxygen evolution reaction (OER) and photoelectrochemical water splitting. Polarized ferroelectric surfaces exhibit switchable surface chemistry, as both polarization orientations endow the surface with different affinities for adsorbates and different band alignments [8, 62]. In OER, the rate‐determining step (RDS) often involves adsorption of OH^−^/O^2−^ intermediates on the catalyst surface, and a polarized ferroelectric could stabilize these intermediates on one face while facilitating their release on the opposite face. Corona‐poled multiferroic layered Bi_5_CoTi_3_O_13_ (BCTO) with in situ grown BiCoO_3_ (BCO) showed pronouncedly enhanced OER efficiency upon spintronic and ferroelectric polarization regulation [78]. This enhancement was traced to stronger chemisorption of oxygen species on the negatively polarized surface, and to accelerated charge transfer enabled by a polarization‐induced rearrangement of oxygen vacancies [67, 78]. More intriguingly, this study reveals the advantages of in situ as‐prepared multiferroic heterostructure for electrocatalysis, where Co^3+^ ions exhibit two distinct electron configurations ((*e*_g_)^3^(*t*_2g_)^3^ and (*t*_2g_)^5^(*e*_g_)^1^) that synergistically enhance OER efficiency (Figure 3j).

Introducing a ferroelectric component can dramatically improve charge separation and surface reaction kinetics – evidenced in a BFO/BiVO_4_ multiferroic heterostructure, where BFO (BiFeO_3_) provides a switchable polarization coupled with visible‐light absorption [79]. Down‐poled BFO/BVO induces favorable band bending at the semiconductor‐electrolyte interface that strengthens the internal field (Figure 3k), drives electron‐hole separation in the desired directions, and thus increases charge injection efficiency (*η_inj_*) while lowering interfacial Faradaic charge‐transfer resistance (*R_f_*). Although up‐poled BFO/BVO weakens interfacial band bending and slows *R_f_* (Figure 3k iii), it still outperforms the unpoled state (Figure 3k,i) because single‐direction polarization eliminates antiparallel‐domain compensation and reduces localized non‐radiative recombination centers, yielding stronger bulk charge separation (*η_bulk_*) and more coherent transport [79]. The two polarization states of BFO presented different surface terminations (Fe‐ vs O‐terminated facets, Figure 3l), where up‐poled BFO/BVO OER becomes adsorption‐rich but transfer‐limited, while its counterpart shows faster kinetics with lower chemisorbed coverage. Such a design could be applied to refine the balance of reactant binding strength and product desorption capability in metal‐air battery electrodes.

Across these examples, ferroelectric polarization introduces an internal electrostatic bias at material surfaces and interfaces, microscopically mediating chemical reactions. By aligning dipoles appropriately, one can stabilize desired reaction intermediates (e.g., LiPS_x_, OH^−^) or expedite charge‐transfer steps, thereby overcoming kinetic bottlenecks. This approach goes beyond traditional catalysts or hosts that rely on static surface functional groups or porosity – the ferroelectric effect is dynamic and can be switched or tuned via external fields, serving as a complementary strategy to optimize battery interfacial reactions in real time [8, 62].

Overall, ferroelectric components should be integrated in a form that complements the battery's operation, whether that is a nanoparticle additive, a coating, a composite electrolyte, or a heterojunction; the geometry and scale must allow the polarization to manifest where it's needed [59, 60]. Battery performance enhancement often requires a combination of ferroelectric engineering with electrochemical architecture design, overcoming rate limitations, stabilizing interfaces, and catalyzing reactions for next‐generation batteries [8]. Understanding ferroelectric mechanisms, from space‐charge layer elimination and ion‐channel formation to dipole‐mediated catalysis, researchers can design battery materials that proactively manage ion and electron distribution at the nanoscale, using built‐in polarization fields to unlock performance levels that were previously out of reach [59, 60, 61, 74, 76].

### 2.3 Ferromagnetism

Across sulfur and oxygen anion chemistries, ferromagnetism couples directly to electrochemical kinetics by reshaping the spin symmetry and orbital hybridization of adsorbates at catalytically active interfaces [37, 38, 39, 40, 41]. Hence, ferromagnetism‐induced spin polarization at catalytic centers directly influences reaction kinetics across chalcogen and oxygen chemistry, via symmetry‐matched electron transfer and *d*‐*p* re‐hybridization that selectively weakens key bonds and reorganizes the free‐energy landscape of the interfacial reaction sequence.

#### 2.3.1 Spin‐State Modulation and Hybridization

**FIGURE 4:**
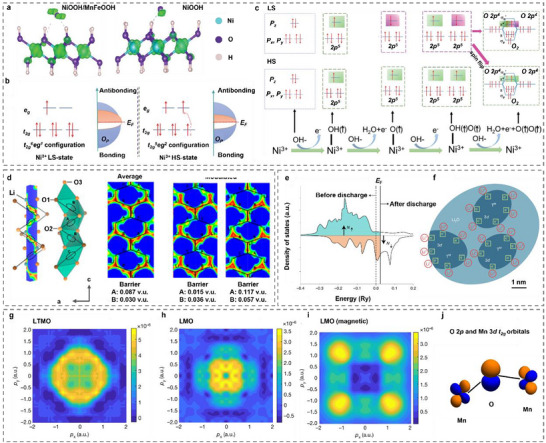
Ferromagnetic order reshapes spin polarization, orbital occupancy, and interfacial electronic structure, thereby altering catalytic reaction pathways, spin‐polarized charge storage, and Li‐ion diffusion behavior in battery‐relevant materials. (a–f) Electronic spin modulation in heterostructured catalysts. Reproduced with permission [38]. Copyright 2024, John Wiley and Sons. (a) Spin‐polarization difference maps for NiOOH/MnFeOOH and NiOOH; green contours denote spin‐up electron density. (b) Schematic of Ni^3+^ electronic configurations in low‐ and high‐spin states. (c) Comparison of the OER pathway with and without spin alignment. (d) Structural and bond‐valence sum analysis of the Li‐ion diffusion pathway in the “average” LiFeBO_3_ structure with *C*2/*c* symmetry. Reproduced with permission [88]. Copyright 2012, American Chemical Society. Dashed circles indicate the locations of the A, B, and C “tetrahedral” sites external to the edge‐sharing LiO_5_ polyhedral chains that enable Li‐ion diffusion along the [001] chain direction, shown superimposed both onto the polyhedral chains (center of figure, two views rotated by 90°) and onto a tubular square section of the 3D valence map running through the center of the LiO_5_ chains shown in the same orientation. The distance between the sections is 1.2 Å, and the colors indicate a Δ*V* ranging from 0 (red) to 0.16 and larger (blue), with the portions of the map that are not blue representing the lowest energy positions for Li within the LiFeBO_3_ lattice through which Li can most easily diffuse. Areas A, B, and C are part of the potential diffusion pathway for Li, and correspond to bridging T “tetrahedral” sites that Li might pass through as it diffuses along the [001] LiO_5_ chains. (e, f) Schematic of surface capacitance with spin‐polarized electrons at the Fe/Li_2_O interface. Reproduced with permission [89]. Copyright 2021, Springer Nature. (e) Schematic of spin‐polarized density of states at the surface of ferromagnetic metal grains (before and after discharge), which is opposite to the bulk spin polarization for the case of Fe. (f) Formation of a space charge zone in the surface capacitance model for extra lithium storage. (g–j) Reconstructed 2D‐EMD maps. Reproduced with permission [89]. Copyright 2021, Springer Nature. (g) Map of the anionic redox orbitals in LTMO that correspond to a lithium concentration between *x* = 0.8 and *x* = 0.4. (h) Map of the cationic redox orbitals in Li*_x_* Mn_2_O_4_ that correspond to a lithium concentration between *x* = 1.079 and *x* = 0.496. (i) Map of the magnetic *t*_2g_ orbitals in Li*_x_* Mn_2_O_4_ extracted from magnetic Compton profile simulations [91]. (j) Diagram showing oxygen 2*p* and manganese *t*_2g_ orbitals. A weak *π*‐type antibonding interaction exists between the oxygen 2*p* and manganese *t*_2g_ orbitals [92]. *p_x_* and *p_y_* are the momentum components parallel to the [100] and [010] directions, respectively, in the Brillouin zone.

Ferromagnetic‐element‐based materials’ inherent spin density and magnetic moments could be exploited for accelerating reaction kinetics [80, 81]. In sulfur conversion, Fe‐N_4_ single‐atom sites display spontaneous spin polarization that populates spin‐selected metal *d* states and maximizes *d*‐*p* hybridization with the *p*‐dominated polysulfide frontier orbitals. This effect weakens the S─S bond in Li_2_S_2_ and lowers the free‐energy barrier for the Li_2_S_2_‐Li_2_S RDS, mechanistically explaining the origin of accelerated Li_2_S nucleation [37]. The partial density of states (pDOS) further shows an almost vanishing bandgap at Fe‐N_4_ and explicit overlap between sulfur *p* (*p*_z_, *p*_x/y_) and Fe *d* (*d*_z_^2^, *d*_xz_/*d*_yz_) orbitals, providing a direct electronic route for faster interfacial electron transfer during the solid‐solid conversion [37]. Quantitatively, Fe‐N_4_ exhibits the largest spin density and magnetic moment (≈1.9 *μ*_B_) among Fe/Co/Ni analogues; reduced antibonding occupancy in Li_2_S_2_‐Fe‐N_4_ correlates with the strongest binding to S‐containing intermediates, establishing spin polarization as the driver of bond‐activation at the Li–S cathode interface. Also, ferromagnetism can align interfacial electrons’ spin states with radical intermediate, lowering barriers for multi‐electron steps. In Al–S cells, where single‐atom Fe on porous carbon (PC‐SAFe) shows the highest spin polarization and unpaired‐electron count, raising the energy of antibonding states in the hybrid orbitals and thereby facilitating charge transfer to polysulfides during both discharge and charge. The mechanistic picture is corroborated by in situ Raman tracking of S_8_→S_2_^−^→S^2−^ transformations and by the explicit formulation of the Al–S half‐reactions [82]. Beyond Li–S/Al–S batteries, ferromagnetism‐oriented spin‐state modulation has also been applied to Zn‐air battery. For oxygen electrocatalysis, reconstructing the Ni/MnFe_2_O_4_ heterostructure in situ into NiOOH/MnFeOOH induces a higher Ni spin state, promotes spin alignment of oxygen intermediates, and enables a more favorable OER pathway (Figure 4a–c). This reduces the potential‐determining OER barrier and shifts the potential‐determining step to an earlier step, yielding an overpotential of ≈0.31 eV in theory and 261 mV at 10 mA cm^−2^ in experiment, with Zn‐air cells sustaining high OCP and >1000‐cycle durability [38]. Hence, ferromagnetism‐induced spin polarization at catalytic centers directly influences reaction kinetics across chalcogen and oxygen chemistry, via symmetry‐matched electron transfer and *d*‐*p* re‐hybridization that selectively weakens key bonds and reorganizes the free‐energy landscape of the interfacial reaction sequence.

#### 2.3.2 Charge and Mass Transfer

Beyond interfacial bond‐activation, ferromagnetic order governs charge and mass transport relevant to batteries [83, 84]. In the canonical double‐exchange (DE) framework, ferromagnetic alignment of mixed‐valence Mn^3+^/Mn^4+^ maximizes Mn‐O‐Mn orbital overlap, broadens the *e_g_* bandwidth, and drives an insulator‐metal transition near the Curie point. The A‐site‐ordered quadruple perovskite Pb(Pb_1/3_Hg_2/3_)_3_Mn_4_O_12_ explicitly demonstrates that enlarging the Mn‐O‐Mn angle to ≈153° strengthens DE and yields colossal magnetoresistance with |MR| up to 2250% at 16 *T* around *T_C_* ≈ 120 K [85]. While devised outside battery operation, spin alignment co‐determines carrier mobility through exchange‐controlled bandwidth, implying that any redox‐active oxide whose conduction involves TMO networks will show conductivity that is sensitive to magnetic order. In layered conjugated metal organic frameworks (MOFs), transport anisotropy provides a complementary, interface‐relevant pathway: edge‐on oriented Cu_2_[PcM‐O_8_] films conduct predominantly along the interlayer direction with measurable Hall mobility (∼4.4 cm^2^ V^−1^ s^−1^), indicating that oriented, π‐stacked pathways can support directional, potentially spin‐preserving interfacial conduction [39].

In lithium metal and sulfur systems, ferromagnetic‐field coupling alters ion motion directly through magnetohydrodynamic (MHD) forces [86]. Under a modest external field, magnetized ferromagnetic Fe_3_Ga scaffolds deepen Li^+^ penetration, redistribute the diffusion layer by Lorentz‐force‐driven convection, and suppress dendrites [87]. Concomitantly, the nucleation overpotential for Li plating drops from ≈137 to ≈22 mV at 5 mA cm^−2,^ and the charge‐transfer resistance decreases, evidencing a reduced kinetic barrier for metal deposition. Mechanistically, the same external field enhances *d*‐*p* electron‐cloud overlap around the Fermi level in *d*‐*p* hybridized Fe_3_M catalysts, accelerating Li–S reaction kinetics while the MHD effect homogenizes Li‐ion flux – thereby coupling ferromagnetic‐tunable electronic and hydrodynamic channels in a single device. Finally, bulk lattice order modulates how magnetic exchange and ionic transport co‐develop. In LiFeBO_3_, a commensurate modulation doubles the *a*‐axis periodicity, opens a ∼3.5 eV gap, and reorganizes 1D Li pathways with higher diffusion barriers than those in the unmodulated analogue (Figure 4d); spin‐projected DOS calculations in the ferromagnetic cell highlight O/Fe orbital mixing at the valence edge, underscoring that correlated‐electron rearrangements track with Li‐migration channels [88]. These transport studies, from DE to MHD and modulation‐controlled diffusion, therefore bridge ferromagnetism to electrochemistry by showing that spin order and its field control reshape both the electronic bandwidth available for charge transfer and the mesoscale flux patterns that govern roughness, dendrites, and polarization [85, 87].

#### 2.3.3 Spin‐Sensitive Charge Storage and Redox‐Orbital Symmetry

In situ magnetometry on conversion‐type TMOs shows that already‐reduced metallic nanoparticles continue to store charge during low‐voltage discharge by accumulating large populations of spin‐polarized electrons within a Thomas‐Fermi screening depth, forming a surface space‐charge zone while balancing Li^+^ accumulation at grain boundaries (Figure 4e,f) [89]. The measured change in magnetization quantifies a surface capacitance that dominates the anomalous “extra capacity” in Fe_3_O_4_/Li and generalizes to CoO, NiO, FeF_2_, and Fe_2_N (Figure 4f). This establishes a causal chain from electron spin population to stored charge and delivered capacity, formalizing ferromagnetism as an active state variable in battery operation rather than a passive materials attribute.

On the cathode side of oxygenated systems, tomographic reconstruction of oxygen redox orbitals in lithium‐rich oxides images the symmetry and phase of the active O‐2*p* states in *operando*. Spin‐resolved pDOS and COOP analyses identified antibonding O‐2*p* character just above the Fermi level (*E*_F_) and reveal how electrostatic and covalent contributions evolve with Li content [90]. Mechanistically, interfacial electron transfer from ferromagnetic catalysts should be most efficient when the spin of injected carriers and the symmetry of the accepting anion orbitals are matched, as illustrated by reconstructed redox‐orbital maps and O 2*p*‐Mn *t_2g_* orbital interactions in lithium transition‐metal oxides (Figure 4g–j). This spin‐orbital matching is precisely the mechanism exploited in the NiOOH/MnFeOOH OER system (Figure 4a–c), where raising the Ni spin state aligns the spin of adsorbed oxygen radicals, facilitates triplet O─O bond formation, lowers the OER overpotential, and shifts the potential‐determining step (PDS) [38].

Overall, ferromagnetism modifies interfacial reaction coordinates by providing spin‐polarized electrons that selectively fill or depopulate antibonding states (Li–S and Al–S), it governs transport by exchange‐controlled bandwidth and spin‐coherent pathways (DE oxides and oriented semiconductors), and it couples to mass transport through MHD forces that homogenize ion flux under field (Li metal and Li–S). Critically, spin‐aware diagnostics close the loop by turning magnetization changes and redox‐orbital maps into quantitative proxies for stored charge and orbital availability, enabling mechanistic prediction of when ferromagnetism will accelerate a given electrochemical step and when symmetry or spin‐forbidden transitions will bottleneck it.

### 2.4 Ferroelasticity

Ferroelasticity emerges from a hierarchical competition between elastic strain energy and twin‐wall interfacial energy [42], producing multivariant domain patterns that minimize the total free energy and reconfigure under external stimuli [93]. This variational principle – explicitly balancing bulk elastic, electric, and interfacial terms – sets the foundation for how microstructures accommodate and redirect stress during electrochemical operation, and therefore how they can be tuned for battery performance. In ferroelastic and ferroelectric ceramics, the equilibrium twin density and lamellar width scale with grain size, with critical grain sizes below which twinning is suppressed and above which more complex banded patterns appear. These scaling rules provide the first quantitative bridge between processing (grain growth), microstructure (twin density), and macroscopic compliance under cycling loads [94].

#### 2.4.1 Chemopiezoelectrics in Perovskites

**FIGURE 5:**
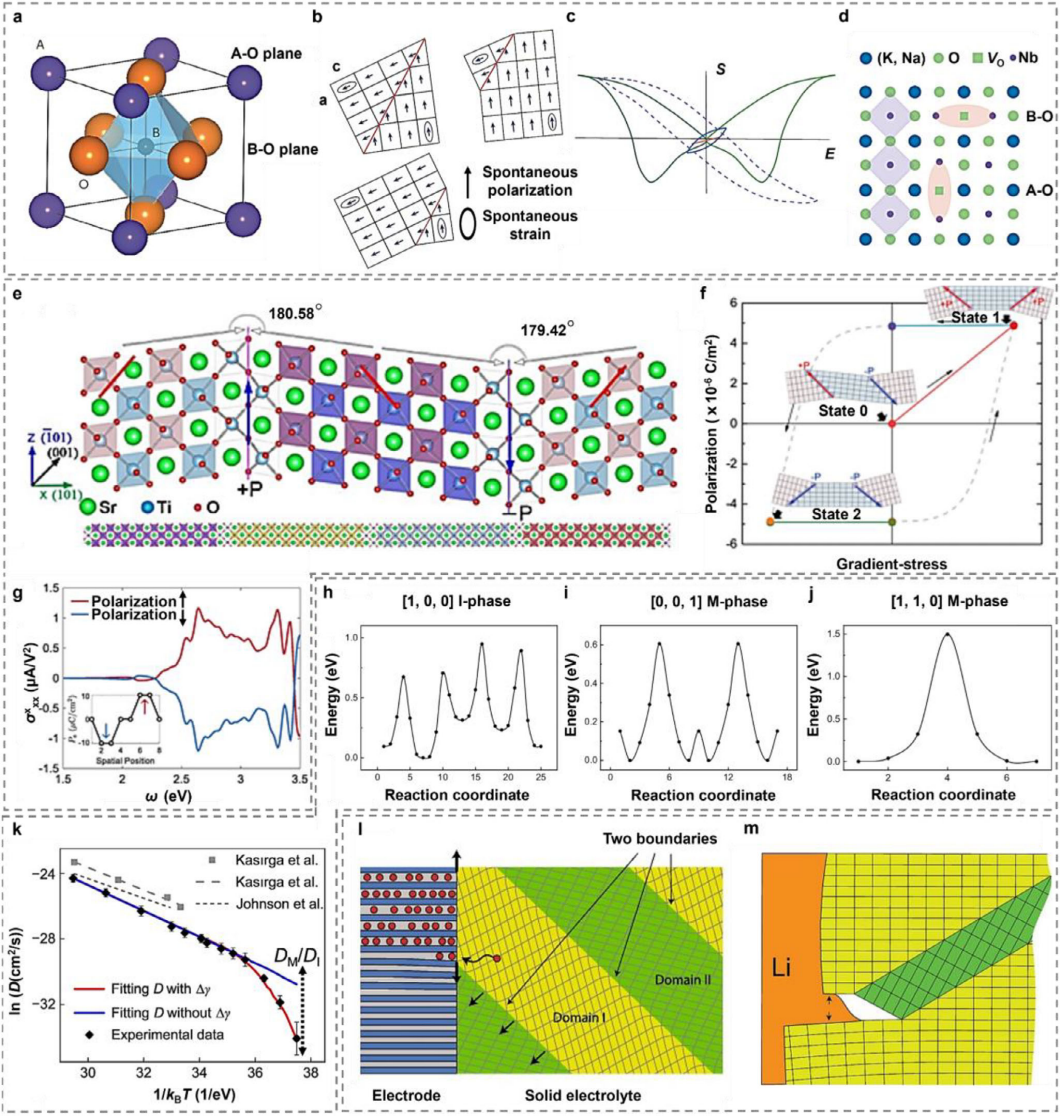
Ferroelasticity and chemopiezoelectricity turn strain, defects, and twin‐wall structures into active transport and toughening elements, enabling vacancy‐coupled electrostrain, polar/conductive twin walls, phase‐dependent ion diffusion, and crack‐deflecting mechanical accommodation in solid‐state battery architectures. (a–d) Electrostrain arising from the chemopiezoelectric effect. Reproduced with permission [96, 109]. Copyright 2025, Springer Nature. (a) Cubic perovskite ABO_3_ structure, highlighting A‐site (e.g., Pb, Ba, K, Na, Bi) and B‐site (e.g., Zr, Ti, Nb, Ta, Fe, Mg, Zn) cations and their potential substitution by dopants or modifiers; A‐O and B‐O planes are indicated, with the BO_6_ octahedron centered on the B‐site cation. (b) Schematic of a 90° domain cluster and its electric‐field‐induced shape evolution when the field is applied parallel to the domain wall (red lines). The symmetric configuration (top left) corresponds to zero field, whereas asymmetric configurations (top right and bottom left) represent domain‐wall displacement under opposite field directions; arrows and ellipses denote spontaneous polarization and strain (one per domain for clarity), with a and c indicating crystallographic axes (tetragonality exaggerated). (c) Strain response regimes: intrinsic piezoelectric strain at low field (red), domain‐wall‐mediated hysteretic strain at moderate fields (blue), butterfly loop under large‐scale domain switching (green), and a hypothetical defect‐associated strain contribution (dashed purple); loop magnitudes are schematic. (d) Pseudo‐2D projection of (K,Na)NbO_3_, with BO_6_ octahedra (purple) and anisotropic strain tensors (red ellipses) surrounding oxygen vacancies (green squares), illustrating vacancy‐induced repulsion between adjacent Nb cations. (e–g) Twin‐wall‐mediated polarization and nonlinear optical response. Reproduced with permission [98]. Copyright 2024, American Physical Society. (e) Atomistic structure of a twin wall (TW), with exaggerated octahedral tilts; blue arrows indicate spontaneous polarization parallel to the TW. (f) Polarization versus gradient‐stress hysteresis in an SrTiO_3_ membrane containing two TWs; insets depict polar states (1 and 2) and the nonpolar state (0) in the corresponding regions. (g) Nonlinear optical (NLO) response localized at the TW; inset shows the local electric polarization along the x direction. (h–k) Phase‐dependent hydrogen diffusion and transport modeling in VO_2._ Reproduced with permission [101]. Copyright 2024, Springer Nature. (h–j) Calculated lowest‐energy path and energy barriers for hydrogen diffusion along the [1,0,0]_I‐phase_, [0,0,1]_M‐phase_, and [1,1,0]_M‐phase_ directions, respectively. (k) Effective hydrogen diffusivity in VO_2_ fitted using two models: a single Arrhenius fit to high‐temperature data without an additional barrier (blue line) and a model incorporating an extra low‐temperature barrier (red line). High‐temperature literature data are included for comparison, showing consistent slopes [110, 111]. The dashed arrow (*D*_M_/*D*_I_) denotes the diffusivity ratio between the M‐phase W_0.015_V_0.985_O_2_ film and the I‐phase VO_2_ at the same annealing temperature. (l, m) Ferroelastic‐toughening mechanisms in ASSBs. Reproduced with permission [106]. Copyright 2016, Springer Nature. (l) Schematic of the proposed transformation‐toughening mechanism: Li intercalation into the electrode induces interlayer expansion (left), generating strain that is accommodated by the expansion of favorably oriented ferroelastic domains in the solid electrolyte (right). The rectangular variant domains and diffuse twin boundaries arise from a square‐to‐rectangle martensitic transformation, modeled using gradient theory of nonconvex elasticity at finite strain. (m) Ferroelastic toughening mitigates stress concentration via crack‐tip blunting, thereby reducing stress intensity as Li dendrites propagate along cracks.

Building on classic ferroelastic classifications and domain‐wall orientation rules, ABO_3_ perovskites (Figure 5a) host rich interplays between ferroelastic transitions, domain reorientation (Figure 5b), and transport or electronic phase changes, foreshadowing the electromechanical couplings now leveraged in electrochemical environments [95]. Mobile oxygen vacancies can act as elastic dipoles that generate anisotropic local strains and, when driven by fields or chemical potential gradients, contribute a dominant extrinsic component to electrostrain while fragmenting domains into smaller, more mobile units – an effect that can be visualized at the atomistic level as vacancy‐induced strain tensors in perovskite lattices (Figure 5d) [96]. Using chemical expansivity *β*c as an atomistically calibrated gauge, lattice‐parameter changes can be converted into sub‐unit‐cell resolved oxygen‐vacancy maps, directly linking defect thermodynamics to local strain fields in cathode‐relevant cobaltites and enabling deliberate placement of vacancy sources/sinks at ferroelastic‐compatible interfaces [97].

Domain walls are not passive boundaries; in ferroelastic perovskites and titanates, they can be polar/flexoelectric and even conducting, as evidenced by localized polarization at twin walls (Figure 5e), tunable gradient‐stress polar states in membranes (Figure 5f), and enhanced local photovoltaic conductivity at polar walls (Figure 5g) [98]. Thereby, ferroelastic twin architecture can seed anisotropic transport pathways and interfacial polarization that couple to charge and ion motion. Halide perovskites exemplify how the “chemical nature” of ferroelastic twin walls controls mesoscale mechanics and transport; their ferroelasticity reorganizes microstructure and interfaces, offering a sensitive electromechanical lever that is mechanistically transferable to battery electrodes and interfaces despite different carrier species [99, 100].

In VO_2_, hydrogen experiences a threefold higher migration barrier and over an order‐of‐magnitude lower diffusivity when crossing a metal‐insulator domain wall, due to an additional volumetric energy penalty associated with reducing the latent heat [101]. This “super‐susceptibility” implies generally retarded atomic diffusion in phase‐transforming systems where the transformation temperature is coupled to composition. Figure 5h–j shows the calculated minimum‐energy paths and diffusion barriers for hydrogen along the [1,0,0] direction in the I phase and the [0,0,1] and [1,1,0] directions in the M phase, while Figure 5k compares effective hydrogen diffusivity in VO_2_ fitted with a single Arrhenius model and a model including the additional low‐temperature barrier, together with consistent high‐temperature literature data. As a result, diffusion along the microbeam axis [0,0,1] is faster than perpendicular to the axis direction in the pure M phase. This shows that ferroelastic domain architecture can modulate ionic diffusion landscapes, which could be transferred to solid‐state electrolyte design, engineering both microscopic ferroelastic domain walls and macroscopic strain textures to modify Li^+^ migration energy landscapes. Researchers can try to establish percolating low‐barrier channels and concurrently strengthen solid‐state electrolytes’ mechanical compliance, achieving dual enhancement.

#### 2.4.2 Strain Management

Oxygen vacancy concentration and site preference synergistically pose impacts on elastic dipoles, local anisotropic strains, and domain‐wall mobility, thus defect and coordination engineering control the extrinsic electrostrain that relieves cycling stresses [102]. Chemopiezoelectric framework shows that thin perovskite oxides with higher near‐surface vacancy densities exhibit large, robust electrostrains that add to intrinsic piezoelectric and ferroelastic switching, offering a stress‐buffering channel during redox expansion/contraction [96]. In cobaltate perovskites, classic mechanical tests already demonstrate that ferroelastic domain switching increases fracture toughness and dissipates energy at crack tips – evidence that cathodes with switchable variants should be less prone to crack growth under oxygen stoichiometry cycling or lithiation‐induced expansion [99]. Complementarily, hydration‐driven chemical expansion and elastic modulus must be co‐optimized for proton‐ceramic air electrodes, by engineering ferroelastic accommodation to maintain percolation and contact at interfaces [103]. At buried heterointerfaces, polar/rotostrictive twin walls in titanates suggest routes to stabilize space‐charge layers and bias ion distributions, providing a mechanically addressable interfacial dipole for charge‐transfer control [98].

In solid electrolytes [104], ferroelastic and transformation toughening mechanisms – via facile twin‐boundary migration – may suppress catastrophic fracture during cycling and accommodate cathode lattice‐parameter oscillations, directly targeting dendrite‐assisted cracking and interfacial debonding (Figure 5l,m) [105, 106]. Recent analyses show symmetry breaking and weak martensitic transformations that create twinned microstructures can, despite lower bulk symmetry, open diffusion channels or provide diffuse twin boundaries with “more cubic” character that function as superionic conduits at the mesoscale – thereby reconciling mechanical toughness with high ionic conductivity [105]. In halide perovskite, ferroelasticity reorganizes interfaces and microstructure, reinforcing this logic for sulfide/oxide SSEs that must maintain intimate, low‐impedance contact under stress [100]. More importantly, ferroelastic switching can alter ionic flux and interfacial chemistry, evidenced in layered Ruddlesden‐Popper Li_2_SrNb_2_O_7_ that exhibits in‐plane Li^+^ conduction, memristive responses, and tunable ferroelasticity [71]. Collectively, electric poling and field‐cooling reconfigure ferroelastic/ferroelectric states, alter Li‐vacancy distributions at electrodes, and shift macroscopic conductivity by orders of magnitude. Overall, defect‐driven anisotropic strain [107], twin‐wall polarity/conductivity [108], and domain mobility serve as transferrable design levers to facilitate transport and mechanical resilience in heterostructures for battery materials such as solid electrolytes.

Hence, a rational ferroelastic design strategy for batteries should integrate three scales. At the atomic scale, oxygen‐ and lithium‐vacancy chemical expansivity must be calibrated and used prospectively to place compressive/tensile fields where they stabilize the required variants and interfacial contact – by controlling stoichiometry gradients during synthesis and cycling, then validating via unit‐cell‐level lattice metrology [97]. At the mesoscale, domain topology must be engineered to guide ionic paths and electric fields. Macroscopically, grain‐size control and texturing can modulate twin density and compliance windows modulations, leveraging the known scaling of twin lamellae with grain size to maximize reversible ferroelastic deformation without sacrificing intergranular transport [94].

Although ferroelastic strain accommodation could, in principle, also benefit conventional liquid‐electrolyte batteries, its current functional significance is more direct in ASSBs. In liquid‐electrolyte systems, such as Ni‐rich layered cathodes and Si‐based anodes, stress accumulation certainly drives anisotropic deformation, particle cracking, and structural degradation; however, once cracks form, the liquid electrolyte can still infiltrate the newly generated surfaces and maintain local ionic access. Under these conditions, degradation rapidly evolves into a coupled chemo‐mechanical process dominated by CEI/SEI re‐formation, electrolyte decomposition, transition‐metal dissolution, and other parasitic interfacial reactions, rather than by immediate interruption of ionic percolation itself. This mechanistic distinction explains why conventional Li‐ion batteries have been improved mainly through electrolyte optimization, interphase engineering, coatings, binders, and particle/microstructure design, whereas explicit ferroelastic‐domain engineering has not yet emerged as an established strategy. By contrast, in ASSBs, where ion transport depends on persistent solid‐solid contact across brittle interfaces, crack formation and interfacial debonding directly block ionic pathways and amplify local current constriction; therefore, ferroelastic switching, twin‐boundary motion, and transformation toughening can simultaneously relieve stress, deflect cracks, and preserve transport continuity. In this sense, ferroelasticity should not be regarded as intrinsically irrelevant to liquid‐electrolyte batteries, but its electrochemical leverage is presently much greater in solid‐state architectures, where mechanical accommodation is itself a first‐order transport and stability variable.

### 2.5 Coupled Multiferroic Systems

In coupled multiferroic systems, multiferroicity serves as a complementary design principle to engineer charge transfer, catalytic pathways, and ion transport. Below, we extract three mechanistic insights directly translatable to battery architectures [112].

#### 2.5.1 Magneto‐Electric Effects

Magnetoelectric multiferroics exploit coupling between magnetic and ferroelectric orders so that one can be controlled by the conjugate field of the other [112, 113]. Spaldin and Ramesh formalized how composite and single‐phase multiferroics realize cross‐control of magnetization and polarization, establishing the symmetry and interface requirements for efficient magnetoelectric coupling [114]. This framework shows that magnetoelectric coupling converts coupled ferroelectric‐ferromagnetic order into electrochemical control variables: electric or magnetic fields can reversibly write polarization and magnetization, thereby reprogramming interfacial boundary conditions such as band bending/space‐charge, local ionic chemical potential, and charge‐transfer barriers [115].

**FIGURE 6:**
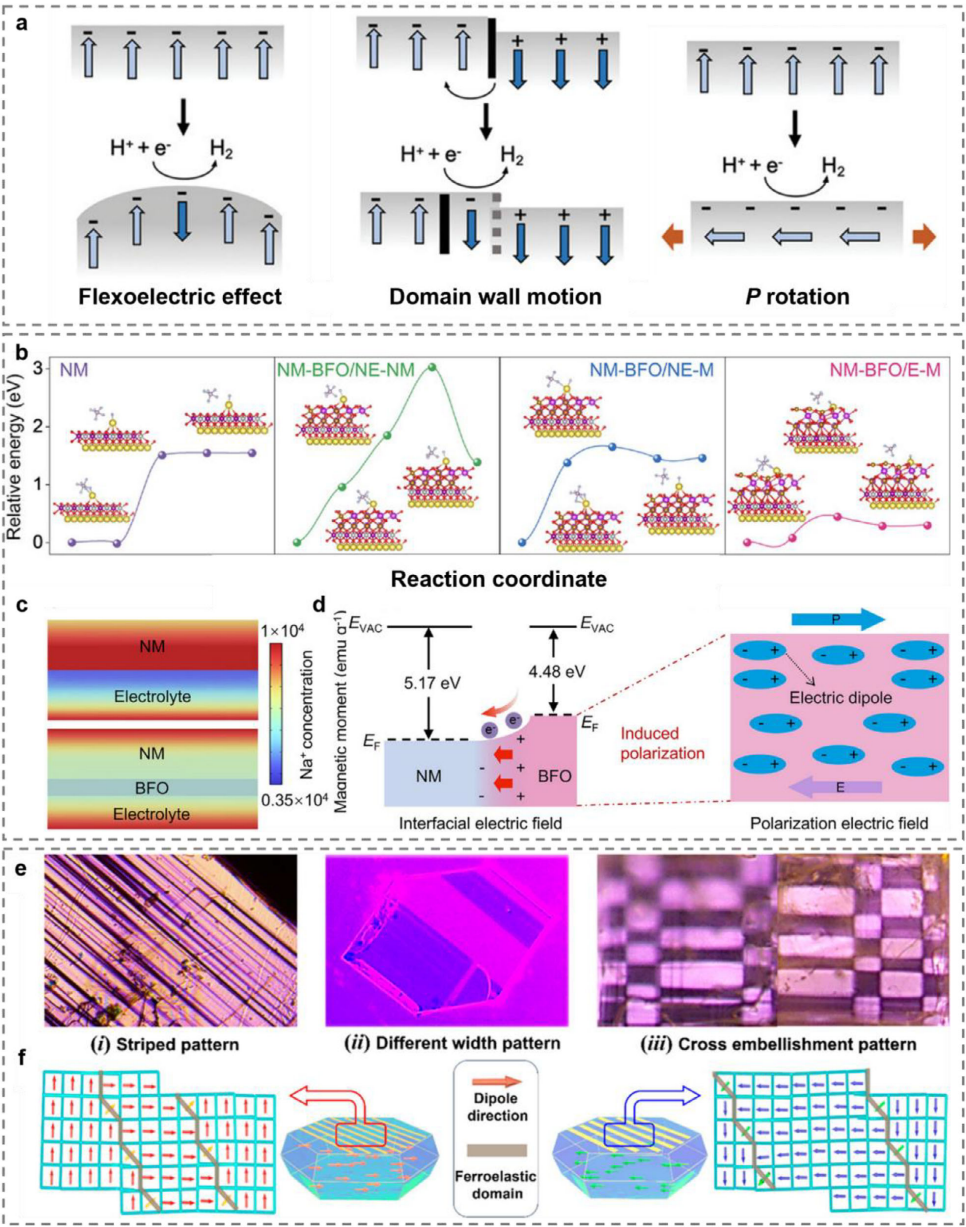
Coupled multiferroic responses – magnetoelectric polarization switching, interfacial electric‐field reconstruction, and field‐driven ferroelastic domain evolution – can cooperatively tune electrolyte decomposition, interfacial energetics, and stress‐adaptive microstructures to refine electrochemical behavior. (a) Magnetoelectrically driven polarization reversal mechanisms in core‐shell nanoparticles: (left) strain‐gradient‐induced flexoelectric switching, (middle) strain‐mediated domain‐wall motion, and (right) tensile‐strain‐driven in‐plane polarization rotation. Reproduced with permission [116]. Copyright 2022, John Wiley and Sons. (b‐d) Interfacial modulation of NaPF_6_ dissociation and magnetoelectric polarization. Reproduced with permission [9]. Copyright 2025, John Wiley and Sons. (b) Simulated dissociation of P‐F species from NaPF_6_ on NM, NM‐BFO/NE‐NM, NM‐BFO/NE‐M, and NM‐BFO/E‐M surfaces, with the corresponding energy barriers. (c) Magnetic hysteresis loop of NM and NM‐BFO‐2; (d) Schematic illustration of the interfacial electric field originating from the work‐function difference between NM and BFO, the resulting BFO polarization and polarization‐induced electric field, together with COMSOL‐simulated polarization and polarization electric field after BFO modification. (e, f) Ferroelastic domain structures and their field‐driven evolution. Reproduced with permission [117]. Copyright 2024, American Chemical Society. (e) Optical images of ferroelastic domains on sheet‐like MFP single crystals, showing (i) striped domains, (ii) alternated domain width, and (iii) cross‐embellished domain patterns. (f) Schematic of ferroelastic domain evolution under applied electric and mechanical stress fields.

In the CoFe_2_O_4_‐BiFeO_3_ (CFO‐BFO) core‐shell nanocatalysts, an alternating magnetic field drives magnetostriction in the CFO core, elastically transferred into the BFO shell, which in turn reverses BFO ferroelectric polarization via magnetoelectric coupling [116]. The polarization reversal generates uncompensated surface charge and band bending at the BFO‐electrolyte interface, providing the driving force for magnetically triggered hydrogen evolution in the absence of applied bias (Figure 6a) [116]. Here, the magnetic field is transduced into a local electrochemical potential via coupled ferroic order, illustrating a remotely addressable, spatially penetrative stimulus to activate buried interfaces – highly relevant for dense battery stacks such as ASSB, flow configurations, and metal‐air electrodes where optical access is limited.

In a BiFeO_3_‐mediated multiferroic interlayer on NaNi_0.5_Mn_0.5_O_2_ (NM) [9], the ferroelectric‐magnetic synergistic state (NM‐BFO/E‐M) lowers the NaPF_6_ *P*‐*F* bond dissociation barrier (Figure 6b) and strengthens binding of NaPF_6_, solvent, and NaF relative to purely ferroelectric or purely magnetic analogues, promoting the formation of a thin, NaF‐rich, inorganic‐dominated cathode/electrolyte interphase (CEI) with suppressed organic accumulation. This coupled state also homogenizes Na^+^ distribution (COMSOL simulation, Figure 6c) and mitigates NiO_6_ distortion at the surface, correlating with ultrafast (50–100 C) kinetics and long‐term cycling stability. This work shows that genuine ferroelectric‐magnetic coupling can be exploited to encode directional interfacial electric fields via polarization (Figure 6d), stabilize desired decomposition pathways and robust inorganic interphase chemistries, and use magnetic or electric stimuli to reconfigure interface energetics.

#### 2.5.2 Domain‐Mediated Transport and Stress‐Adaptive Functionality

Coupled ferroelectric‐ferroelastic systems extend multiferroicity into the lattice degree of freedom, making strain and domain architecture active state variables for electrochemistry – highly pertinent under repeated battery volume changes. In Li_2_SrNb_2_O_7_, ferroelasticity, ferroelectricity, and antiferroelectricity coexist in a layered Ruddlesden‐Popper framework, with electric poling reconfiguring ferroelastic/ferroelectric domains and inducing giant in‐plane memristive behavior [71]. Enhanced Li^+^ in‐plane conduction and tunable rectification arise from poling‐driven accumulation/depletion of Li^+^ vacancies at crystal‐electrode interfaces, directly tied to domain configuration, suggesting ionic distribution and ferroelectric polarization are mutually coupled. Such behavior foreshadows solid electrolytes and interlayers where mechanical/field history modifies ion‐conduction pathways and interfacial barriers, enabling self‐adaptive current homogenization, resistive memory integrated into electrodes, or domain‐guided Li^+^ highways. In a ferroelectric‐ferroelastic hybrid perovskite, (cyclohexanemethylaminium)_2_PbCl_4_ (MFP), where polarization vectors and strain tensors are strictly cross‐linked and inter‐switchable (Figure 6e) [117]. The dense hierarchy of ferroelastic domains under both electric and mechanical fields establishes a model in which internal stress, lattice distortion, and polarization cannot vary independently (Figure 6f). Such fully coupled systems suggest thin‐film interlayers or solid electrolytes whose ionic/electronic conductivity tensors reorient with stress or poling, allowing batteries to dynamically redistribute local current in response to chemo‐mechanical loading. Bismuth oxychalcogenides (Bi_2_O_2_Se, Bi_2_O_2_S, Bi_2_O_2_Te) add a crucial ingredient: high carrier mobility combined with ferroelectric/ferroelastic order. Wu and Zeng predict that Bi_2_O_2_X phases host switchable in‐plane or out‐of‐plane polarization and ferroelasticity, where ferroelastic switching rotates the polarization and simultaneously reorients the high‐mobility conduction axes, while strain can further induce giant polarizations and tune the bandgap [118]. Such coupling makes Bi_2_O_2_X archetypes for electrode or SSE materials in which stress, polarization, and conductivity are entangled, enabling domain‐engineered anisotropic transport and robust, nonvolatile control of interfacial band alignment. Overall, ferroelectric‐ferroelastic coupling converts unavoidable mechanical fields (volume change, pressure, dendrite‐induced stress) into a potential control lever for ionic and electronic transport. Coupled multiferroics serve as proofs of concept for stress‐adaptive solid electrolytes, ferroelastic‐buffered interfaces, and domain‐encoded fast‐ion pathways directly relevant to high‐rate solid‐state and metal‐air batteries.

## 3 Tuning Multiferroicity

Currently, battery materials are predominantly designed with conventional strategies – doping [119], defect [120], and entropy engineering [121]. Many reported optimizations remain largely empirical and phenomenological, with limited connection to clearly defined functional targets. This limitation is particularly acute for multiferroic systems, where tuning ferroic order demands a mechanistic understanding of its microscopic origin and of how these order parameters translate into space‐charge regulation, interfacial optimization, transport anisotropy, and mechanical resilience, as outlined in the preceding sections. In practice, the relationship between multiferroic properties and electrochemical performance is intrinsically multi‐variable and non‐linear, so that meaningful gains rely not only on incremental compositional changes, but also on rigorously designed, experimentally verifiable tuning strategies.

Despite rapid progress, no review papers have systematically distilled the latest, mechanistically reliable design schemes for tuning multiferroicity specifically in battery‐relevant materials. Moreover, many concepts established in conventional multiferroics for electronics‐magnetoelectric coupling, field‐programmable polarization, ferroelastic toughening, and spin‐selective transport have not yet been fully translated into electrochemical applications. To bridge this gap, this section consolidates and re‐frames the available strategies into three overarching, actionable categories: (i) multiphysics field control, (ii) architectural engineering, and (iii) multiferroic‐targeted defect engineering. Together, these provide a coherent design and fabrication roadmap for future multiferroic‐centric energy materials, enabling deliberate rather than incidental exploitation of coupled ferroic orders in next‐generation batteries.

### 3.1 Multiphysics Field Tuning of Multiferroicity for Battery Applications

#### 3.1.1 Magnetic Field

**FIGURE 7:**
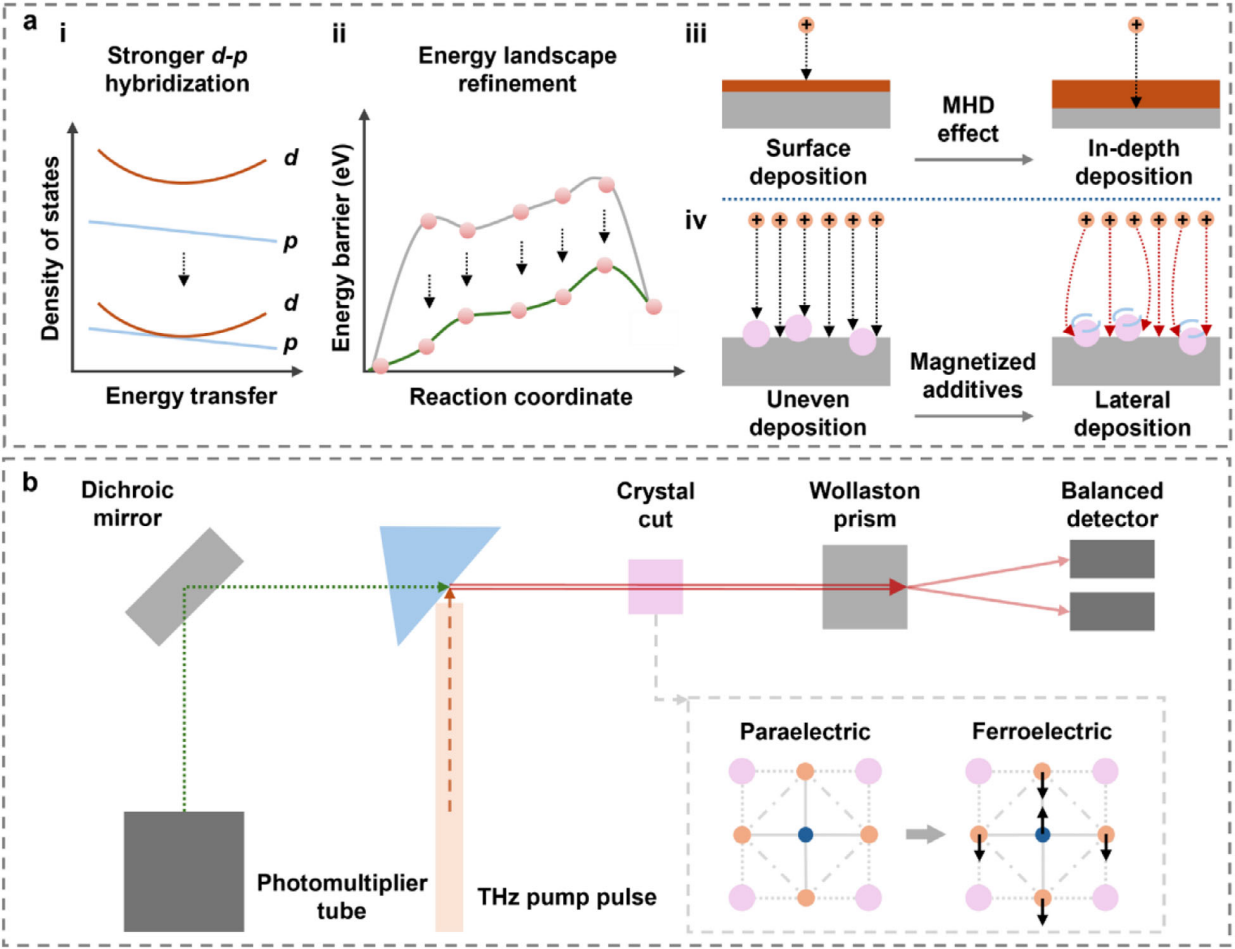
External magnetic and terahertz fields can actively reconfigure ferroic order and transport by tuning spin‐dependent electronic structure, ion‐migration energetics, ion‐flux uniformity, and even inducing transient ferroelectric‐like states, thereby offering dynamic field‐based routes to control battery reactions and deposition behavior. (a) Schematic illustration of magnetic‐field‐mediated effects in battery‐relevant and multiferroic systems [87]: (i) A schematic of DOS graph, where the red curve represents *d* orbital and the blue line represents *p* orbital. spin polarization enhances *d*‐*p* hybridization near the Fermi level, refining the electronic structure for improved adsorption and catalytic activity. (ii) A schematic of energy landscape refinement. Magnetic fields reshape the local energy landscape and can reduce ion‐migration barriers within the lattice. (iii) MHD convection homogenizes ion distributions and promotes deeper, more uniform penetration; and (iv) ferromagnetic additives generate Lorentz‐force‐guided ion trajectories that favor lateral, dendrite‐suppressing metal deposition. (b) THz pump‐probe scheme in SrTiO_3_ (STO) as a proof of concept for ultrafast field‐driven ferroic control: a single‐cycle THz field lowers the crystal symmetry, observed via partial depolarization (THz Kerr effect) and THz‐induced second‐harmonic generation, with the inset for (100)‐cut STO illustrating the THz‐driven evolution from a paraelectric to a ferroelectric‐like state. Reproduced with permission [124]. Copyright 2019, American Association for the Advancement of Science.

Magnetic fields have emerged as a versatile tool to modulate electrochemical processes by influencing ionic transport and the electronic/spin state of materials. In Li–S systems, an external magnetic field can exert Lorentz forces on charged species to alter lithium polysulfide (LPS) migration pathways, while aligning the spins of magnetic components (Figure 7a). This dual effect enhances electron transfer kinetics and reaction rates, and can even induce net magnetic moments in ferromagnetic electrodes for improved charge‐transfer processes [122]. Sun et al. employed ferromagnetic bimetallic Fe_3_M (M = Al, Si, Ga, Ge, Sn) nanostructures as sulfur electrocatalysts and conductive scaffolds, and showed that applying a ∼280 mT field fostered the Fe‐M *d*‐*p* hybridization around the Fermi level, leading to higher intrinsic catalytic activity for polysulfide conversion (Figure 7a i, ii) [87]. Simultaneously, the magnetized Fe_3_M framework guided Li^+^ diffusion uniformly via Lorentz forces and MHD convection, facilitating dendrite‐free lithium deposition (Figure 7a iii, iv), underscoring that external magnetic fields can simultaneously enhance ferromagnetic cathode reaction kinetics and stabilize lithium metal anodes. Additionally, magnetic‐field‐induced spin polarization, shifting electrons from low‐spin to high‐spin configurations, increases the number of unpaired electrons and alters surface energy, which can strengthen bonding interactions and promote faster electrochemistry [122].

Magnetic ordering directly influences the kinetics of reactions involving open‐shell species via spin selection rules [116], which can be tuned by electric fields via magnetoelectric coupling, enabling reversible spin‐polarization control at catalytic surfaces [116, 123]. In oxygen electrocatalysis, triplet O_2_ formation in the OER proceeds more efficiently on ferromagnetically aligned surfaces, where the spin angular momentum of surface states matches that of the adsorbed *OO species. Conversely, spin‐forbidden transitions are suppressed, improving selectivity and potentially reducing overpotentials. The catalytic utility of this mechanism extends to spin filtering, as orbital angular momentum determines the spatial symmetry of spin‐polarized surface states and their overlap with adsorbate orbitals, establishing selective spin channels and stabilizing specific intermediates [24]. These phenomena illustrate that magnetic fields can directly tune ferromagnetic/antiferromagnetic order and magnetic moments in Li–S and metal‐air battery components, thereby coupling to their electrochemical behavior.

#### 3.1.2 Terahertz Field

Terahertz (THz) field offers a unique route to dynamically manipulate lattice vibrations and coupled ferroic orders on ultrafast timescales [125]. Intense THz electric field pulses can resonantly drive soft phonon modes in incipient ferroelectrics, thereby inducing non‐equilibrium ferroelectric or multiferroic states that are inaccessible under static conditions. Li et al. applied a single‐cycle THz pulse to selectively excite the 2.7 THz polar soft mode of quantum paraelectric SrTiO_3_ [124]. The THz field coerced ions to move collectively along the reaction coordinate of the ferroelectric distortion, lowering the crystal symmetry and generating a macroscopic dipole moment. This THz field‐induced ferroelectric state persisted transiently after the pulse, evidenced by distinct changes in the phonon spectrum and second‐harmonic generation signals (Figure 7b). Essentially, the THz excitation steered the lattice directly into a new crystalline configuration within picoseconds, achieving “collective coherent control” over materials structure. Beyond inducing ferroelectricity, THz fields can also generate magnetization in nominally non‐magnetic materials via dynamical multiferroicity. Basini et al. applied an intense circularly polarized THz field to excite two degenerate phonon modes with a 90° phase offset, continuously rotating lattice electric polarization, which in turn induced a magnetic moment through the coherent rotation of ionic charges and converted lattice angular momentum into electronic spin alignment [126]. The underlying mechanism aligns with the theoretical notion that a time‐varying polarization *P*(*t*) can generate a magnetization (analogous to a Barnett effect on the crystal lattice). Overall, THz light coherently controls lattice vibrations to induce materials’ magnetization that exhibits neither static ferroelectric nor magnetic order, thus driving multiferroicity. These THz‐driven effects demonstrate that high‐frequency fields can dynamically bridge ferroelectric and magnetic degrees of freedom with specific phonon modes of electrode/solid‐electrolyte materials, and access metastable polarized states for dielectric modulations such as inducing ferroelectric‐like surface charge ordering to influence ion transport, potentially tuning phonon‐ion coupling or triggering phase transitions for ionic conductivity enhancement [7, 127, 128, 129].

#### 3.1.3 Built‐In Electric Field

**FIGURE 8:**
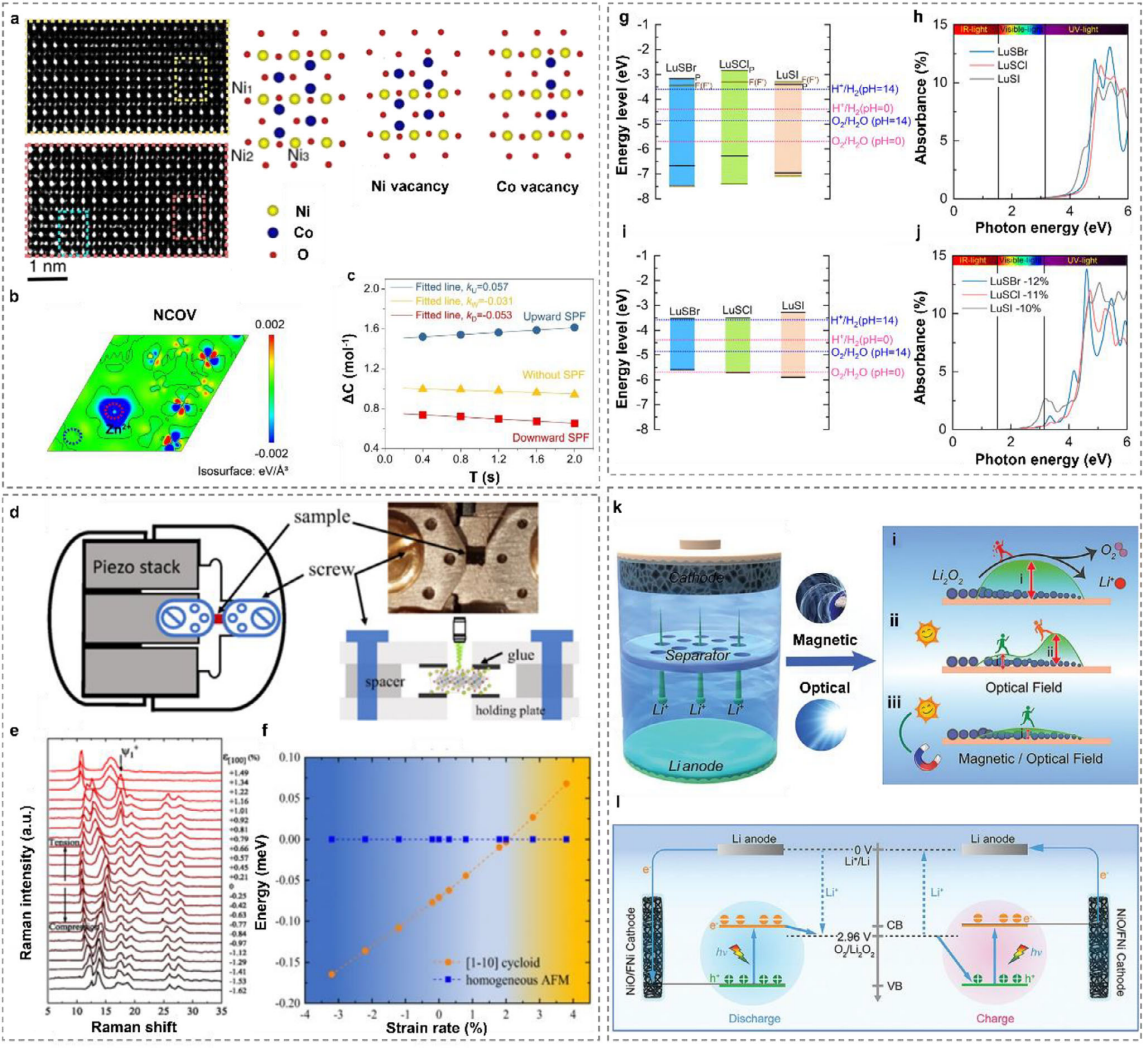
Built‐in electric fields, strain, magnetic fields, and light can be used as practical external or internal tuning knobs to reshape ferroic states, interfacial charge transfer, Zn/Li deposition behavior, band alignment, and reaction pathways, thereby improving transport uniformity and electrochemical reversibility across multiple battery systems. (a–c) Ferroelectric‐induced built‐in electric field facilitating charge transfer and suppressing dendritic growth in Zn‐air battery. Reproduced with permission [132]. Copyright 2023, John Wiley and Sons. (a) Atomic‐resolution filtered scanning transmission electron microscope (STEM) images along the [010] zone axis: the yellow (upper) and red (lower) regions reveal Ni vacancies (larger missing atomic columns) and Co vacancies (smaller missing atomic columns), respectively, while the blue region shows a relatively intact NCO lattice. (b) 2D electron localization function (ELF) map of NCOV on the (111) plane; black lines denote charge equipotential contours, with the Co vacancy marked by a red circle near Zn^2+^ and the Ni vacancy by a blue circle. (c) Correlation between zinc deposition time and the overall electrolyte concentration gradient. (d–f) Uniaxial–strain control of multiferroic properties. Reproduced with permission [133]. Copyright 2023, American Physical Society. (d) Schematic of the uniaxial strain application system viewed from below (piezoelectric stack), including an enlarged view of the strain apparatus and a cross‐sectional illustration showing sample fixation. (e) Low‐energy Raman spectra highlighting strain‐dependent spin excitations. (f) Calculated energy comparison of relaxed bulk BFO for two magnetic configurations: the type‐I spin cycloid state with propagation vector along [1, 2, 3, 4, 5, 6, 7, 8, 9, 10, 11, 12, 13] and the homogeneous G‐type antiferromagnetic state. (g–j) Strain‐dependent band alignment and light absorption. Reproduced with permission [135]. Copyright 2025, Royal Society of Chemistry. (g) Band‐edge positions of strain‐free LuSBr, LuSCl, and LuSI monolayers relative to the water redox potentials, calculated using the HSE06 functional; band edges of the F, P, and F′ phases are indicated by distinct lines, with the vacuum level referenced to 0 eV. (h) Calculated light‐absorption spectra of the three monolayers using HSE06. (i, j) Band‐edge positions (i) and light‐absorption spectra (j) of the same monolayers under applied compressive strains of ‐12% (LuSBr), ‐11% (LuSCl), and ‐10% (LuSI). (k, l) Magnetic‐ and optical‐field‐assisted operation of Li‐O_2_ batteries. Reproduced with permission [136]. Copyright 2022, John Wiley and Sons. (l) Schematic of Li–O_2_ battery operation under different external environments (left) and illustration of discharge‐product decomposition during charging (i‐iii, right), highlighting the underlying electrochemical mechanism. (l) Proposed working principles of discharge and charge processes under an optical field.

Electric fields – either externally applied or internally generated [130] – are a direct means to tune ferroelectric polarization and electrostatic interactions in battery materials [30, 131]. One promising strategy is to incorporate materials with spontaneous ferroelectric polarization into battery components, tailoring defect chemistry and creating built‐in electric fields (BEFs) that modulate ion transport and deposition. Bai et al. combined cation‐vacancy‐enriched NiCo_2_O_4_ (NCO, Figure 8a) that increases lattice spacing and ion storage sites via Ni/Co vacancies (Figure 8b) with a ferroelectric layer that establishes a built‐in electric field (BEF) to construct a cathode heterostructure in zinc‐ion batteries (ZIBs) [132]. While BEF facilitated electron/ion transfer into the cathode, Figure 8c demonstrates the ferroelectric spontaneous polarization field‐induced electrolyte gradient mitigation and Zn^2+^ repelling from certain facets, thus suppressing zinc dendrite growth on the anode. Tao et al.’s embedment of BaTiO_3_ (BTO) nanofibers into a solid polymer/ceramic electrolyte showed that this yields a self‐regulating interface for Li metal anodes [59]. The ferroelectric domains of BTO introduce local internal fields, and its polarization lowers the local electrochemical overpotential for Li plating, while BTO's piezoelectric effect counteracts dendrite tips means that mechanical stress generates additional electric field feedback. Together, these effects led to a more uniform Li^+^ flux and smoother metal deposition, achieving dendrite‐free cycling at Li‐metal anode in an ASSB using LiFePO_4_ cathode and a BTO‐embedded PEO‐garnet electrolyte. Also, K_0.5_Na_0.5_NbO_3_‐based ferroelectrics with oxygen vacancies have shown giant electrostrain under AC electric fields via chemopiezoelectric effect, and field‐driven redistribution of charged defects bears resemblance to how Li^+^ intercalation induces expansion in battery electrodes [96, 109]. Notably, the built‐in field concept could be generalized to other systems: for instance, polar interfaces or charged coatings on Li‐metal anodes might distribute the electric field more uniformly and mitigate dendrites or SEI heterogeneity. By exploiting ferroelectricity (BEF), dielectric polarization, and electrostriction, researchers can systematically tune ferroelectric and ferroelastic aspects of battery components, linking these multiferroic properties to improved charging efficiency, ionic conductivity, and stability.

#### 3.1.4 Strain Engineering

Strain engineering has been extensively employed in model multiferroics like BFO to manipulate its coupled ferroelectric and magnetic order, as it directly modifies crystal structure, lattice symmetry, and thus ferroic order parameters. Continuously varying uniaxial strain on bulk BFO can be used to in situ tune its polarization magnitude and antiferromagnetic spin structure [133]. Using an elasto‐Raman apparatus (Figure 8d), Hemme et al. applied incremental tensile strain and observed that BFO's ferroelectric soft mode intensity grows markedly under tension (Figure 8e), indicating an enhancement of ferroelectric polarization with strain [133]. Figure 8f compares the energies of the G‐type antiferromagnetic state and the [131] spin cycloid over −3% to +4% strain, showing that the cycloid is favored under compressive and small tensile strains but is overtaken by the homogeneous G‐type antiferromagnetic state above ∼+2% tensile strain, where low‐energy magnon modes associated with the cycloidal order are concomitantly suppressed, signaling a strain‐driven transition from the native cycloid to a homogeneous antiferromagnetic state. This novel approach could be transferred to electrodes fabricated with multiferroic additives.

Strain engineering can also tune the electronic structure and reactivity of battery electrode materials and catalysts. In Li–S batteries, imposing lattice strain on sulfur host materials has been shown to optimize the adsorption and conversion of polysulfides by shifting the *d*‐band center of catalyst surfaces, thereby adjusting their chemical bonding with lithium polysulfide species [122]. Zhao et al. found via DFT that applying strain to a TiC_3_O_3_‐terminated MXene altered the Ti─O/C bond lengths and lowered the overlap of Ti *d*‐orbitals, thereby shifting its *d*‐band center to ∼1.31 eV (closer to *E*_F_), improving sulfur utilization and translating to better capacity retention in Li–S cells [134]. Ma et al. identified 2D ferroelastic LuSX (X = Cl, Br, I) monolayers’ large spontaneous strain and the ability to cycle between two crystallographic orientations under applied stress, offering the intriguing ability to undergo reversible strain‐driven phase transitions [135]. As shown in Figure 8g,h, across the F, F', and P phases of LuSBr and the LuSCl/LuSI monolayers, the conduction band minimum (CBM) remains above the H_2_/H^+^ potential and the valence band maximum (VBM) below the O_2_/H_2_O potential over pH 0–14, satisfying the thermodynamic requirements for overall water splitting. Meanwhile, Figure 8i,j show that polymorph, composition, and strain systematically modulate the bandgaps and band‐edge positions, providing a versatile design parameter for electrochemical enhancements [135]. In a battery context, ferroelastic materials could be used to dynamically adjust an electrode's electronic structure or ionic diffusion pathways when a field or stress is applied. Even without external strain, the material's inherent reversible strain (ferroelastic domain switching) endows it with flexibility in electronic properties. In essence, strain can strengthen a material's ferroelectricity and modulate its ferroelectric‐like polarization and electronic states, also reconfigure its antiferromagnetism, thus implementing strain engineering on electrodes/interface can enhance battery reactions.

#### 3.1.5 Coupled Fields and Future Potential

Applying multiple types of fields in tandem can unlock synergistic effects on materials’ multiferroic properties and battery performance, via the interplay between different stimuli to drive more pronounced or complementary modifications in a material. Wang et al. simultaneously applied light and a magnetic field to a 3D porous NiO cathode to improve the oxygen reaction kinetics (Figure 8k). Under illumination, the NiO cathode behaves like a photoelectrode, generating electron‐hole pairs that participate in the oxygen evolution and reduction reactions, yet is limited by fast electron‐hole recombination. By introducing a magnetic field during illumination, trajectories of electrons and holes were deflected by the Lorentz force in opposite vectors, causing an effective spatial separation of charge carriers and achieving an ultralow charging potential of ∼2.73 V with minimal polarization growth in the Li–O_2_ cell (Figure 8l) [136]. Coupled‐field approaches can also leverage materials that inherently link multiple ferroic orders – one could modulate a catalyst's magnetic state and electronic structure via an applied electric field or use a magnetic field to switch the polarization of a ferroelectric separator. In a battery, a magnetoelectric coating on an electrode might allow the battery's own internal electric field (during charge/discharge) to adjust the magnetic properties of the coating in real time, potentially affecting catalysis of reactions like polysulfide conversion (since magnetic ordering can influence *d*‐band occupancy and reactivity) [122]. Similarly, piezoelectric‐ferroelectric materials inherently couple mechanical and electric fields; as demonstrated for BaTiO_3_‐based interphases and solid electrolytes, stress associated with Li protrusion growth can generate local piezoelectric/ferroelectric field feedback that redistributes Li^+^ flux and suppresses dendrite amplification [59, 137]. More speculatively, external acoustic stimulation may offer a complementary route to homogenize plating or relax concentration gradients, as surface‐acoustic‐wave and acoustic‐field studies have already shown improved ion transport and more uniform Li deposition [138, 139]. However, such multifunctional additives or field‐coupled schemes must remain electrochemically stable and avoid adverse mechanical side effects, since some ultrasound conditions can also exacerbate dead‐Li formation [140].

### 3.2 Architectural Engineering

**FIGURE 9:**
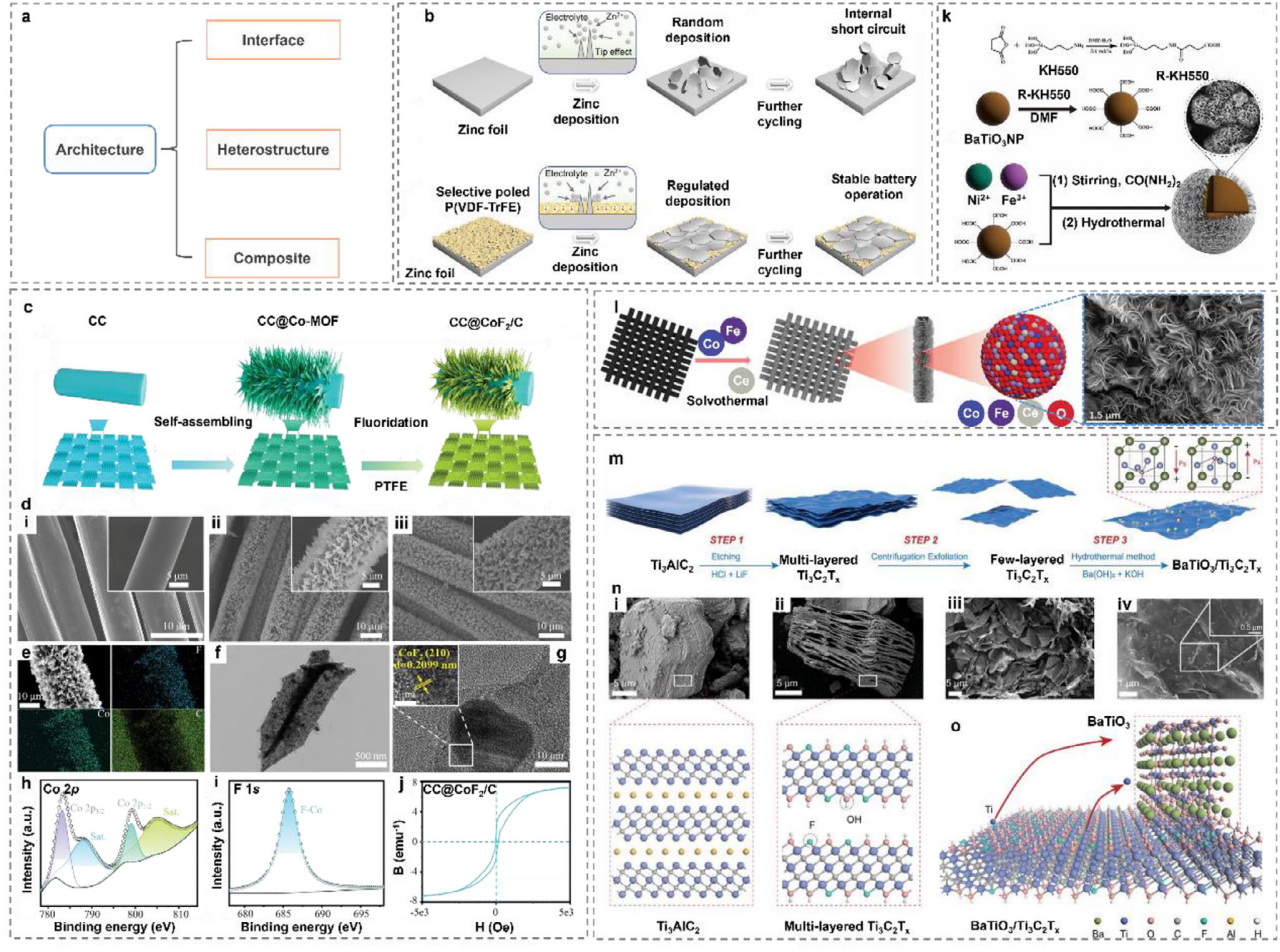
Architectural engineering – through interfaces, heterostructures, and composites – can embed and amplify ferroic functionality in practical battery materials, enabling polarization‐guided metal deposition, magnetically active current collectors, ferroelectric‐catalyst heterojunctions, and ferroelectric nanocomposites that collectively improve charge transfer, deposition uniformity, and energy storage performance. (a) There are three major approaches to modulate materials’ architecture – interface, heterostructure, and composite. (b) Schematic illustration showing the zinc‐deposition behaviors on bare zinc (top) and selectively poled P(VDF‐TrFE)‐coated zinc (bottom). Reproduced with permission [141]. Copyright 2022, John Wiley and Sons. (c–j) Synthesis and characterization of CC@CoF_2_/C. Reproduced with permission [146]. Copyright 2025, John Wiley and Sons. (c) Illustration of the synthesis procedures of CC@CoF_2_/C. (d) SEM images of (i) CC, (ii) CC@Co‐MOF, and (iii) CC@CoF_2_/C. (e) Elemental mapping of F, Co, and C elements in a single CoF_2_/C nanosheets decorated Carbon Cloth. (f) TEM of CoF_2_/C. (g) HRTEM of CoF_2_/C. XPS spectrums of (h) Co 2*p* and (i) F 1*s* for CoF_2_. (j) Magnetization hysteresis loops of the CC@CoF_2_/C. (k) Scheme demonstrating the preparation of the flower‐like core‐shell t‐BTO@NiFe‐LDH heterojunctions [147]. (l) Schematic illustration of the fabrication of CoFeCe‐2 and SEM image of CoFeCe‐2 [149]. (m–o) Nano‐ferroelectric BaTiO_3_/MXene (Ti_3_C_2_T_x_) composites for enhanced lithium storage. Reproduced with permission [150]. Copyright 2024, John Wiley and Sons. (m) Schematic diagram of the experimental process for synthesizing BT/f‐Ti_3_C_2_T_x_ composites. The inset shows the unit cell of tetragonal BaTiO_3_ with Ti center displacements. (n) SEM images of (i) Ti_3_AlC_2_, (ii) multi‐layered Ti_3_C_2_T_x_, (iii) few‐layered Ti_3_C_2_T_x_ and (iv) BT/f‐Ti_3_C_2_T_x_ composites. (o) Strategy for the in‐situ growth of BaTiO_3_ nanoparticles using metastable Ti atoms on f‐Ti_3_C_2_T_x_ nanosheets.

Architectural design of materials at the nanoscale offers a powerful lever to control multiferroic properties – ferroelectric polarization, ferroelastic strain effects, and magnetic ordering – at battery‐relevant interfaces. By employing tailored coatings, heterostructures, and composites, researchers have demonstrated precise tuning of ferroelectric and ferromagnetic responses to enhance interfacial electrochemistry (Figure 9a). Below, we review how these strategies enable built‐in electric fields, interface strain, and internal magnetic fields to improve performance in cathodes, anodes, solid electrolytes, and electrocatalysts.

#### 3.2.1 Interface Control and Coating

##### 3.2.1.1 Ferroelectric Polymer Layers for Dendrite‐Free Metal Anodes

Ultrathin ferroelectric coatings on metal anodes can create localized electric fields that guide ion flux and suppress dendrite growth. A polarized poly(vinylidene fluoride‐trifluoroethylene) PVDF‐TrFE film (∼0.5 µm) on zinc metal is a clear example [141]. The permanent dipole field of this ferroelectric polymer attracts Zn^2+^ to under‐charged areas, enforcing a uniform Zn‐ion distribution along the anode surface (Figure 9b). This “guided growth” mechanism forces zinc to plate horizontally in a dense, planar manner rather than as spiky dendrites. As a result, symmetric Zn‐Zn cells with the ferroelectric‐coated anode ran for over 2000 h at 0.2 mA cm^−2^ (0.2 mAh cm^−2^ per cycle) without short‐circuit, and could sustain high rates up to 15 mA cm^−2^. Such a thin polarized layer achieved dendrite‐free Zn deposition with minimal impact on energy density, showcasing ferroelectric surface charge engineering as a viable protective strategy. Building on this, a composite interlayer combining a ferroelectric phase with a functional oxide has yielded even greater stability for Zn anodes. In one design, Cr‐doped ZnO nanoparticles were integrated into a PVDF matrix to form a ferroelectric‐zincophilic coating on Zn [64]. The Cr doping imparts ZnO with switchable polarization, introducing a uniform internal electric field across the interface to promote thermodynamically favored epitaxial Zn growth (layer‐by‐layer plating) rather than random mossy deposits. Meanwhile, the ZnO component provides high zincophilicity (abundant nucleation sites for Zn), and its inherent hydrophobicity suppresses side reactions like the hydrogen evolution reaction. This highlights how multifunctional coatings can unify electric‐field guidance (from ferroelectric domains) with chemical advantages (nucleation sites, passivation) to stabilize reactive metal interfaces.

##### 3.2.1.2 Ferroelectric Coatings at Solid‐State Interfaces

At cathode‐electrolyte interfaces in ASSBs, ferroelectric coatings can mitigate space‐charge phenomena and improve Li^+^ transport. As mentioned, coating GClO_4_ on LiCoO_2_ cathodes develops an internal flexoelectric strain that locks it into a single‐domain, upward‐polarized state, where the built‐in electric field can effectively pull Li^+^ across the solid electrolyte interface and therefore improve lithiation kinetics [142]. Thus, strain‐engineered ferroelectric films can be leveraged to generate favorable internal fields (and associated ferroelastic lattice distortions) that eliminate space‐charge layers and boost ion transfer at solid interfaces [143]. Similarly, in the Na‐ASSB study, KNN was applied as nanoscopic islands on a 3D Na_3_Zr_2_Si_2_PO_12_ (NASICON) framework, forming a “core‐shell” structure at the particle level. The KNN coating was made ultrathin (1‐3 layers) to allow its polarization to be switched by the interfacial field of the cell [61]. This design ensured that ferroelectric domains in KNN could reversibly align with each charge‐discharge cycle, continuously minimizing the space‐charge each time [144]. The remarkable long‐term stability achieved (86% capacity retention over 180 cycles after a 2‐month rest) attests to how effective dynamic ferroelectric screening can be when the interface is engineered for it.

##### 3.2.1.3 Magnetic Coatings for Ion Deposition Control

Magnetic interface coatings have emerged as a tool to influence ion transport via MHD forces. A 3D micromagnetic coating was demonstrated by depositing core‐shell Cu‐Ni “nanonecklaces” onto battery separators, where Ni‐coated Cu nanoparticles present multidirectional magnetic moments. Under cell operation, these flexible dipoles generate localized 3D magnetic fields that stir the electrolyte via Lorentz‐force‐driven convection [145]. The magnetic separator induced strong multidirectional MHD vortex flows, which homogenize the Li^+^ concentration and electric field near the Li metal anode. This efficient charge redistribution leads to uniformly dense lithium nucleation and a robust, LiF‐rich SEI, resulting in prolonged cyclability and high‐loading LiFePO_4_ or NCM cathodes that cycle stably with dendrite‐free Li plating. Ferromagnetic interfacial additives can also be applied directly onto conductive hosts to impose order on ion trajectories, demonstrating a novel magnetically assisted route to dendrite‐free, deeply plated metal anodes [141]. Liu et al. decorated a carbon cloth with nanoscale CoF_2_ (Figure 9c‐j illustrates the fabrication process, basic material morphologies, and properties, for referencing), which is ferromagnetic at operating conditions, acts as an internal micro‐magnet to direct Li^+^ deposition [146]. The CoF_2_/Carbon coating produces a Lorentz‐force influence that guides Li^+^ flux uniformly and concurrently undergoes in situ conversion to a LiF‐enriched SEI, further smoothing the deposits. Using this magnetically active composite layer, a Li metal half‐cell ran for over 10 000 h at 1 mA cm^−2^ with an ultra‐low ∼7.8 mV overpotential, and a Li|LiFePO_4_ full cell maintained ∼122.9 mAh g^−1^ at 2C for 1000 cycles (∼92% capacity retention).

#### 3.2.2 Heterostructure Engineering

Designing heterostructures that intimately couple a ferroelectric material with an electrochemically active phase allows one to harness polarization effects at the interface. Core‐shell architectures of tetragonal BaTiO_3_ nanoparticles wrapped by NiFe layered double hydroxide (LDH) nanosheets (Figure 9k) can induce a built‐in electric field at the BTO/LDH interface, which was shown to tilt the band structure and accelerate electron transfer during OER [147]. Notably, the lattice strain at the coherent BTO/LDH interface made the BTO domains easier to polarize, enhancing surface charge separation and reactivity. Such ferroelastic strain effects – arising from lattice mismatch tension – reinforce the single‐domain polarization in BTO, showcasing how mechanically coupled heterostructures can amplify ferroelectric‐induced catalysis [148]. Likewise, in a BaTiO_3_@MOF‐Fe/Co electrocatalyst, the self‐polarizing BTO nanoparticles (high permittivity ∼270) created a favorable internal field that enhanced charge conductivity within the MOF and stabilized the catalytic active sites, dramatically improving OER kinetics. Overall, the rational design of heterostructures enabled ferroelectric polarization to directly tune surface reaction energetics – an emerging paradigm for noble‐metal‐free electrocatalysts [147, 148]. These design principles can be extended to the development of noble‐metal‐free multiferroic‐catalyst heterostructures at air cathodes, deploying built‐in ferroelectric polarization and ferroelastic strain. A solvothermal‐fabricated trimetallic CoFeCe oxide (Figure 9l), that features nanoscale domains of amorphous and crystalline oxide and yields a coherent interface, has elevated Li–O_2_ battery longevity and serves as another proof of concept for heterostructure engineering [149]. In essence, by integrating multiferroic materials and cathode catalyst as an architected heterostructure, one can modulate its ferroic domains similarly to how internal chemical environments are tuned. Conversely, the amorphous/crystalline pairing may introduce ferroic effects that mimic internal lattice stress and defect polarizability, illustrating that any deliberate heterostructure optimizing internal fields or strain can yield outsized electrochemical gains.

#### 3.2.3 Composite Architectures Design

By embedding multiferroic constituents directly into electrode matrices as composites, researchers can impart internal electric fields and structural benefits throughout the bulk material. A BaTiO_3_‐mediated Ti_3_C_2_T_x_ MXene anode prevented MXene restacking via steric hindrance (material fabrication, atomic structure, and morphology are displayed in Figure 9m–o), scavenged metastable Ti species, and even catalyzed the formation of a thin, robust SEI on the MXene surface. While the electrode's operational mechanical and chemical stability were improved, BTO's spontaneous polarization introduced local electric fields that increased Li^+^ adsorption on the MXene and facilitated charge transfer kinetics [150]. Ferroelectric fillers like BTO can be further exploited for their high permittivity and tunable polarization – from accelerating Li^+^ dissociation in electrolytes to reshaping Li deposition morphology. Composite architectures that incorporate ferromagnetic particles into a conductive scaffold represent another form of multiferroic tuning. The previously mentioned CoF_2_‐carbon cloth system can be viewed as a magnetic composite host for Li metal plating, which homogeneously directs ion flux [146]. In general, multiferroic composite construction can realize desirable properties of different phases – high ionic/electronic conductivity, polarization fields, magnetism, and mechanical toughness – into a single material platform. By distributing ferroelectric, ferromagnetic, and ferroelastic phases with controlled volume fractions and engineered interfaces throughout a conductive scaffold or porous framework, it becomes possible to construct a dense, locally connected network of internal electric fields, spin bias, and strain gradients within the electrode, thereby enabling 3D regulation of ion flux, deposition morphology, and interfacial reactions.

### 3.3 Emerging Strategies

#### 3.3.1 Spin Polarization and *d‐p* Orbital Hybridization Modulations

**FIGURE 10:**
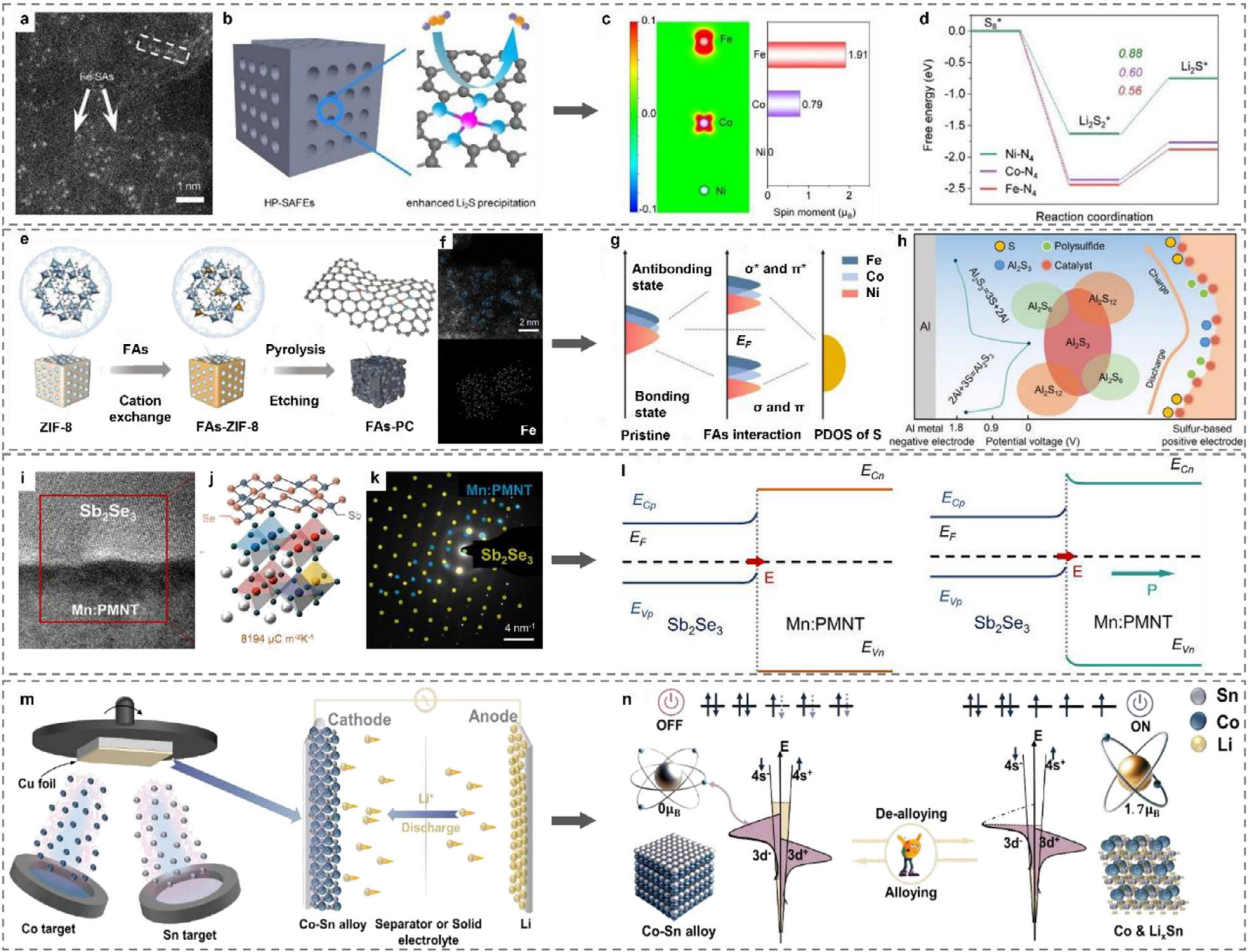
Defect engineering, single‐atom coordination, interface design, and Li‐ion‐driven compositional tuning can be used as practical routes to tailor ferroic‐related electronic structure, and thereby strengthen catalytic conversion, pyroelectric response, and electrochemical functionality in battery‐relevant systems. The left column presents methodological approaches, while the right column highlights the corresponding mechanistic insights. (a–d) Spin‐modulated catalytic design. Reproduced with permission [37]. Copyright 2023, John Wiley and Sons. (a) Schematic of the porous catalyst architecture. (b) AC‐STEM image of HP‐SAFe. (c) Calculated spin density distribution and magnetic moment of the Fe_s_‐N_4_ active site. (d) Free‐energy profiles for the reduction of Li_2_S_2_ to Li_2_S on the Fe_s_‐N_4_ site. (e–h) Single‐atom catalytic regulation in Al–S batteries. Reproduced with permission [82]. Copyright 2025, John Wiley and Sons. (e) Schematic illustration of the synthetic procedure for PC‐FeAs. (f) Atomic‐resolution HAADF‐STEM image with labeled Fe single‐atom sites and the corresponding Fe elemental mapping. (g) Schematic of orbital hybridization between Fe/Co/Ni 3*d* states and the Al_2_S_6_‐Fas‐N_4_. (h) Galvanostatic charge‐discharge profile of the Al–S battery, accompanied by the proposed redox mechanism of sulfur intermediates. (i–l) Interface‐engineered enhancement of pyroelectricity. Reproduced with permission [155]. Copyright 2024, Cell Press. (i) Unit‐cell schematic of Mn‐doped PMNT/Sb_2_Se_3_ heterostructure illustrating doping and interface engineering effects on pyroelectric properties. (j) STEM image of the heterointerface, showing partial lattice mismatch arising from structural disparity between the two materials. (k) SAED pattern of the semiconductor/single‐crystal interface. (l) Schematic band alignment depicting energy‐band evolution at the interface in polarized and unpolarized states. (m, n) Li‐ion‐controlled magnetic modulation in Sn‐Co systems. Reproduced with permission [154]. Copyright 2025, American Chemical Society. (m) Schematic of sputter deposition of a multilayered Sn‐Co film and its voltage‐driven modulation via Li‐ion exchange. (n) Illustration of magnetism tuning in differently alloyed Sn‐Co systems through changes in orbital occupancy and band filling.

In Li–S battery [151], tuning the catalyst's magnetic moment via ferromagnetic dopants can accelerate polysulfide redox kinetics, as the spontaneous spin polarization of ferromagnetic atoms directly correlates with enhanced S─S bond cleavage and long‐life Li–S battery performance. A recent study on Li–S battery cathodes introduced single‐atom Fe, Co, and Ni sites (Figure 10a,b), and revealed Fe‐N_4_ as a ferromagnetic center with the highest spin moment that exhibits the most potent catalytic effect on the sluggish solid Li_2_S_2_ to Li_2_S conversion. DFT calculations showed that the Fe dopant's unevenly distributed *d*‐electrons cause spontaneous spin polarization (Figure 10c), populating antibonding states at higher energy and thereby weakening the S─S bonds in Li_2_S_2_ (Figure 10d) [37]. Such a strategy also improves multielectron conversion in Al–S battery [82]. When in situ growing Fe, Co, and Ni on porous carbon (Figure 10e), single‐atom Fe sites exhibited the most unpaired electrons (Figure 10f), and thereby induced the strongest *d‐p* orbital coupling with polysulfide intermediates. Fe's high spin polarization increases charge transfer to adsorbed Al–S species and raises the antibonding orbital energy of the Fe─S bond (Figure 10g), thereby facilitating faster and more reversible sulfur redox reactions (Figure 10h). Thus, spin polarization can tailor transition‐metal *3d* electron occupancy to stabilize sulfur species for designing high‐performance Al–S electrodes.

At the materials‐synthesis level, this strategy should be realized by constructing atomically dispersed high‐spin Fe sites through heteroatom‐coordinated carbon frameworks, such as MOF‐derived pyrolysis or chelate‐assisted carbonization, which stabilize isolated Fe‐N_x_ motifs, suppress Fe clustering, and tune the ligand field to maximize unpaired 3*d* electrons. Precise control over Fe coordination number, Fe‐N covalency, and the local defect environment can thereby directly regulate Fe 3*d*‐S 3*p* hybridization and the antibonding‐state occupation of adsorbed sulfur species. At the device level, these spin‐polarized Fe sites could be integrated into slurry‐cast sulfur cathodes, catalytic interlayers, or separator coatings as spatially distributed sulfur‐conversion interfaces. Embedding Fe‐N_x_‐functionalized porous carbon into conductive 3D cathode networks enables polysulfide adsorption, charge transfer, and Li_2_S nucleation to occur preferentially at magnetically and electronically optimized sites, thereby translating spin‐polarization modulation into a practical electrode‐engineering route for higher sulfur utilization and more reversible multielectron conversion.

#### 3.3.2 Ferroelectric Order Modulation

Doping and defect control can likewise tailor ferroelectric order, with direct implications for energy conversion. In lead magnesium niobate‐lead titanate, Mn^2+^ substitution was used to induce lattice distortions and disrupt ferroelectric domain regularity, resulting in higher spontaneous polarization, reduced Curie temperature, and therefore an extraordinary 14‐fold increase in the material's pyroelectric coefficient (up to 8194 mC m^−2^ K^−1^) [122]. In parallel, an epitaxial interface integrated with a polar semiconductor (Sb_2_Se_3_) introduces a built‐in electric field across the heterojunction (Figure 10i‐k), adding an interfacial polarization component that supplements the bulk polarization change. Dopant‐induced and interfacial polarization synergistically yielded superior pyroelectric output, suggesting complementary doping and architecture design can maximize ferroelectric domain reconfiguration for applications ranging from thermal energy harvesters to thermal‐charge batteries (Figure 10l) [122]. Overall, ferroelectric order can be tuned through dopant‐induced lattice distortion and defect regulation. For example, Mn^2^^+^ substitution in lead magnesium niobate‐lead titanate perturbs local symmetry and disrupts regular domain organization, thereby increasing spontaneous polarization, lowering the Curie temperature, and sharply amplifying the pyroelectric response. This effect could be strengthened by constructing polar ferroelectric/semiconductor heterojunctions. In an epitaxial interface with polar Sb_2_Se_3_, the interfacial built‐in field adds an extra polarization component to the bulk ferroelectric response, promoting stronger domain reconfiguration during thermal cycling. Together, dopant‐controlled bulk polarization and interface‐engineered internal fields provide a practical route to enhance pyroelectric output in thermal energy harvesters and thermal‐charge batteries.

#### 3.3.3 Strain Heterogeneity and Defects in Perovskite Thin Films

Controlling ferroelastic strain via defect engineering is another avenue to tune material functionality. Orr et al. applied Bragg coherent diffraction imaging to visualize individual grains of mixed‐cation halide perovskites and found distinct nanoscale regions of tensile and compressive strain coexisting within single grains, as well as a surprisingly high density of dislocations forming regular networks [152]. The Sn‐substituted perovskite (Cs_0.15_FA_0.85_SnI_3_) exhibited multiple ⟨100⟩ dislocations and associated antiphase boundaries, whereas a Pb‐based analogue (Cs_0.1_FA_0.9_Pb(I_0.95_Br_0.05_)_3_) showed virtually no such extended defects. This stark contrast implicates composition (B‐site doping) as a key determinant of defect tolerance: introducing Sn^2+^ in place of Pb^2+^ likely increases lattice plasticity or alters defect formation energies, leading to more dislocation generation. Because local strain can modulate electronic band structure and ion transport, these findings highlight that managing strain heterogeneity and dislocation density through controlled doping and processing is crucial for durable performance [152]. By optimizing composition and microstructure (e.g., defect “healing” treatments), one can suppress deleterious ferroelastic domain distortion and cracking, thereby achieving mechanically robust battery electrodes and solid electrolytes with prolonged operational stability.

#### 3.3.4 Voltage‐Driven Ionic Control of Antiferromagnetic/Ferromagnetic Exchange Bias

Beyond static doping, *in operando* ion insertion can reversibly toggle magnetic order, effectively bridging battery chemistry and spintronics. A recent all‐solid‐state device demonstrates voltage‐controlled ionics to modulate an exchange‐coupled ferromagnet/antiferromagnet heterostructure [153]. In this system, a 5 nm Co film (ferromagnetic) is partially oxidized to CoO (antiferromagnetic) and interfaced with a solid Li^+^ electrolyte and Li metal gate – mimicking a nanoscale “battery”. Applying a gate bias drives Li^+^ into the CoO layer, electrochemically reducing CoO to metallic Co (creating oxygen vacancies and Li_x_CoO defects), whereas reversing the bias reoxidizes Co back to CoO. This ionic shuttle enables reversible phase transformation between antiferromagnetic and ferromagnetic states over >1000 cycles without degradation. Crucially, the exchange bias in the Co/CoO bilayer is modulated in tandem – the bias field and coercivity of the ferromagnetic Co change as the adjacent layer switches its magnetic ordering [153]. Because magneto‐ionic switching requires only a small voltage, it offers an energy‐efficient avenue to control magnetization compared to traditional spin‐torque methods. This work illustrates a general principle that defect chemistry under an electric field (here, Li‐ion insertion creating oxygen vacancies) can dynamically tune antiferromagnetic/ferromagnetic order. The result is a new dimension of control for multi‐functional battery materials – e.g., enabling electric‐field‐programmable magnetic properties – with seamless integration into solid‐state devices. This mechanism could be implemented through solid‐state magneto‐ionic heterostructures that integrate a redox‐active magnetic layer with a Li^+^ conductor and metallic Li reservoir. In the Co/CoO model system, voltage‐driven Li^+^ insertion and extraction reversibly switch the adjacent layer between antiferromagnetic CoO and ferromagnetic Co, thereby modulating exchange bias and coercivity in situ. More broadly, this strategy allows researchers to deposit thin ferromagnet/antiferromagnet bilayers onto ion‐conducting solid electrolytes, then use gate‐controlled ion transfer to dynamically regulate magnetic order through reversible redox and vacancy generation. Such architectures may translate magneto‐ionic coupling from a model phenomenon into a device‐compatible platform for low‐energy, reversible control of magnetic functionality in solid‐state electrochemical systems.

#### 3.3.5 Electrochemical Band Filling

An alternative electrochemical strategy to tailor ferromagnetic order relies on band‐filling via alloying. Bu et al. introduced a nonmagnetic metal into a ferromagnetic host using a battery‐like electrochemical cell (Figure 10m), thereby altering the host's valence electron count and magnetic moment in real time [154]. Specifically, in a Sn‐Co alloy film, electrochemical insertion/extraction of Sn was used to add or remove conduction electrons in the Co's *d*‐band (Figure 10m,n). Guided by the Slater‐Pauling rule (which correlates electron concentration with magnetization in alloys), this doping achieves an on‐off magnetic switch (Figure 10n): under a modest bias (∼1.5 V), the 70 nm film transitions from a ferromagnetic state (high moment) to a nearly non‐magnetic state and back, with ∼40 emu g^−1^ magnetization change. *operando* magnetometry confirmed that the modulation is *nonvolatile* (the altered state persists without power) and highly durable over many cycles. Furthermore, the approach was generalized to other systems (e.g. Sb‐Co and Sn‐Fe), each showing substantial magnetization tuning by electrochemical insertion of a *p*‐band metal [154]. Electrochemical band filling provides a straightforward design paradigm for multifunctional battery materials – via dopant selection and voltage input, one can continuously tune a metal's ferromagnetic ordering at room temperature. Such controllable magnetization in bulk electrodes could be leveraged for self‐sensing batteries or magneto‐electric energy storage devices, exemplifying the powerful synergy of doping chemistry and multiferroic property control in modern battery research. This strategy could be implemented by fabricating electrochemically active magnetic alloys whose *d*‐band occupancy is highly sensitive to reversible insertion of *p*‐band elements, for example through co‐sputtering, thin‐film alloy deposition, or composition‐controlled metallurgical synthesis. Precise control over alloy stoichiometry and phase homogeneity is essential to position the material near a magnetically responsive band‐filling regime, where small electrochemical changes in electron count induce large shifts in magnetic moments. At the device level, these alloys should be integrated as redox‐addressable magnetic electrodes in solid‐state or thin‐film electrochemical architectures that enable controlled alloying/dealloying under low bias. This provides a practical route to electrically programming bulk magnetization through reversible band filling, translating alloy chemistry into device‐compatible magnetic switching for self‐sensing and magneto‐electric energy‐storage systems.

## 4 Probing Multiferroicity

Probing ferroic order and multiferroic‐driven effects in battery materials demands characterization strategies that go far beyond widely used conventional tools such as XRD [156], XAS [157], XPS [158], and Raman spectroscopy [159]. These techniques largely report average structure, oxidation state, or bonding environments and are intrinsically insensitive to subtle, spatially heterogeneous polarization, ferroelastic twinning, or spin textures, especially under operating conditions. As a result, multiferroic contributions are often invoked only qualitatively – enhancements are casually ascribed to “ferroelectric effects” while potential ferroelastic toughening, flexoelectric fields, or spin‐polarized pathways remain unresolved or even misassigned – reflecting a methodological gap rather than an absence of coupling. Addressing this gap requires techniques that are explicitly designed to correlate ferroic order parameters with electrochemical function, and to do so in situ *operando* under realistic fields, currents, and chemistries. Accordingly, this section consolidates a targeted methodological framework organized into four pillars: (i) magnetometry for quantifying ferromagnetic and spin‐polarized states relevant to catalysis, transport, and “spin‐capacitive” storage; (ii) multifield probes, including ultrafast spectroscopy, advanced synchrotron radiation and x‐ray methods, and neutron‐based techniques, capable of resolving coupled spin‐charge‐lattice dynamics and buried interfaces; (iii) microscopic approaches (Scanning probe microscopy (SPM)/Polarized light microscopy (PLM) and their electrochemical, mechanical, and magnetic modes) that map local polarization, strain, conductivity, and ion mobility; and (iv) complementary measurements that bridge these ferroic signatures to other material characteristics. By articulating the analysis logic, applicable configurations, and mechanistic readouts for each class – often through battery‐oriented or readily transferrable exemplars – this framework is intended to enable researchers to rigorously identify, quantify, and ultimately engineer multiferroic effects across diverse energy materials, rather than treating them as incidental or anecdotal.

### 4.1 Magnetometry

Magnetic characterization techniques such as superconducting quantum interference device (SQUID) magnetometry [160], and vibrating sample magnetometry (VSM) have become indispensable for probing multiferroicity [161]. By sensitively tracking changes in magnetization under varied stimuli, these tools elucidate how ferromagnetic order interacts with electric polarization and even electrochemical processes. Recently, magnetometry has enabled a mechanistic understanding of ferromagnetism, ferroelectricity, and magnetoelectric coupling in battery materials.

#### 4.1.1 Static Magnetometry Probes

**FIGURE 11:**
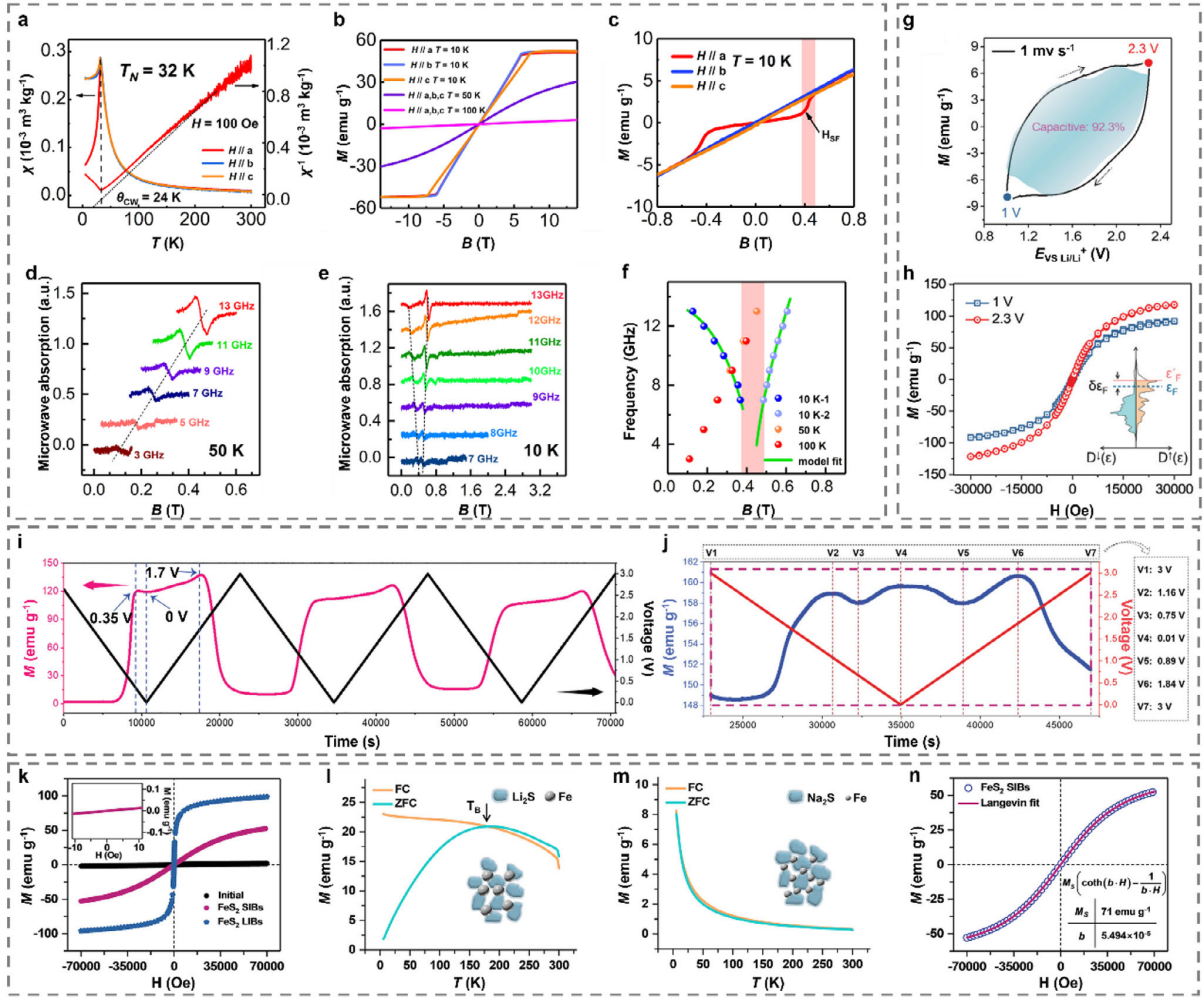
Static and *operand* *o* magnetometry can directly resolve magnetic anisotropy, field‐ or voltage‐induced magnetic‐state switching, and real‐time magnetic evolution during electrochemical cycling, thereby providing a powerful route to track how lithiation/delithiation, phase transformation, and spin‐polarized charge storage reshape battery‐relevant materials. (a–f) Anisotropic magnetic response and field‐induced spin‐flop transition. Reproduced with permission [162]. Copyright 2023, Springer Nature. (a) Temperature dependence of magnetic susceptibility measured along the crystallographic a, b, and c axes; the dotted curve represents the Curie‐Weiss fit. (b) Magnetic field dependence of magnetization (*M*‐*H*) along the a, b, and c axes at selected temperatures. (c) Enlarged minor hysteresis loops along the three axes at 10 *K*, highlighting the spin‐flop (SF) transition. Field‐dependent absorption spectra in the 3–13 GHz range at 50 *K* (d) and 10 *K* (e), obtained from AFMR measurements. (f) Magnetic field dependence of resonance frequency at various temperatures. (g, h) Voltage‐driven lithiation and delithiation of Co/TiO_2_ multilayer films. Reproduced with permission [163]. Copyright 2023, John Wiley and Sons. (g) Cyclic voltammogram of a [Co (0.7 nm)/TiO_2_ (4 nm)]_30_ film electrode measured at a scan rate of 1 mV s^−1^ versus Li/Li^+^. (h) Magnetic hysteresis loops of the as‐prepared film recorded at the two terminal voltages indicated in (g), with the voltage held for 1 h prior to measurement. The inset schematically illustrates the accumulation and dissipation of spin‐polarized electrons at the Co surface during lithiation and delithiation. (i, j) *operando* magnetic response during electrochemical cycling. Reproduced with permission [164]. Copyright 2021, John Wiley and Sons. (i) *operando* magnetic response of the p‐n‐CoO lithium‐ion battery under an applied magnetic field of 3 *T*. (j) *operando* magnetic monitoring of a CoO_1‐x_/Co LIB during CV scanning at an applied magnetic field of 3 T. (k–n) Post‐discharge magnetic characterization. Reproduced with permission [165]. Copyright 2021, American Chemical Society. (k) *M*‐*H* curves of FeS_2_ electrodes discharged to 0.01 V in SIBs and LIBs at 300 K (inset: low‐field region); ZFC/FC measurements included. ZFC/FC magnetization of SIBs (l) and LIBs (m) at 500 Oe; insets show Fe nanograins (buff) dispersed in Na_2_S or Li_2_S (blue). (n) Langevin fit of the SIB hysteresis loop in (k).

VSM provides quantitative, high‐sensitivity magnetization measurements over an exceptional dynamic range (≈10^−8^ to >10^3^ emu; 10^−11^ to 1 A·m^2^), across magnetic hardness (soft to hard) and sample forms (solids, powders, single crystals, thin films, liquids) [161]. Its modular implementations – electromagnets, Halbach arrays, or superconducting magnets for field control; cryostats/furnaces for cryogenic‐to‐high‐T operation; vector detection for anisotropy; and automated first‐order reversal curve protocols – enable rigorous mapping of *M* (H, T), magnetic anisotropy, interactions, and coercivity distributions. This unique combination of sensitivity, field/temperature breadth, sample versatility, and mechanistic diagnostic power makes VSM the indispensable workhorse of magnetometry for materials from ultrathin films to bulk permanent magnets. The complementary magnetometry analysis, which consisted of SQUID, PPMS, along with antiferromagnetic resonance (AFMR) allowed researchers to reveal a field‐induced spin‐flop transition in a type‐II multiferroic van der Waals CuCrP_2_S_6_ (CCPS) crystal (Figure 11a–f) [162]. Using magnetic measurements along different crystallographic directions, they identified a uniaxial easy axis that is common to both the electric polarization orientation and the magnetic order in CCPS (Figure 11a–c). By fitting the magnetization and resonance behavior to a spin Hamiltonian, the anisotropy energy parameters of CCPS were quantified (Figure 11d–f). Such an analytical framework offers a more mechanistically solid pathway to delve into the role of anisotropy and spin reorientation in multiferroic‐based thin‐film solid‐state electrolytes.

#### 4.1.2 *Operando* Magnetometry in Energy Storage Systems

Beyond conventional multiferroics, in situ and *operando* magnetometry has also been applied to battery materials, uncovering magnetoelectric‐like effects that link magnetic properties to electrochemical functionality. *operando* magnetometry to a Fe_3_O_4_ anode enabled real‐time magnetization monitoring during charge/discharge, taking advantage of the fact that discharging Fe_3_O_4_ produces ferromagnetic Fe^0^ nanoparticles and thus resolved the unexplored phenomenon – transition‐metal oxide (TMO) anodes exhibit excess lithium storage capacity beyond their theoretical limits [89]. As the cell was driven to very low potential, the magnetization of the electrode began to decrease even after all Fe_3_O_4_ had been converted to Fe metal, indicating that additional lithium was being stored in a non‐structural manner by injecting electrons into the Fe nanoparticles. The magnetometry data revealed that extra Li^+^ ions are adsorbed at the Fe/Li_2_O interfaces while a corresponding excess of spin‐polarized electrons is accumulated within the Fe metal, creating a charge‐compensated “spin capacitor” at the interface. By quantifying the magnetization change due to this electron accumulation, interfacial space‐charge storage becomes the dominant source of the previously unexplained extra capacity in Fe_3_O_4_.

In a Co/TiO_2_ multilayer film, reversible magnetization switching of ∼30 emu g^−1^ was achieved at room temperature by shuttling Li^+^ ions to and from the TiO_2_ layer, which in turn injects or removes electrons from the adjacent Co layer [163]. *operando* magnetometry that uses a SQUID and VSM confirmed this effect originates from space‐charge induced spin‐polarized electron accumulation on Co, which increases *E*_F_ of 3*d* electrons, enabling non‐volatile magnetization states once the bias is removed (Figure 11g,h). Importantly, these results establish that magnetometry can quantitatively capture voltage‐driven magnetization changes in real time, providing direct evidence of magnetoelectric coupling through reversible ion intercalation in ferromagnetic metals. In CoO anodes, *operando* magnetometry measurements confirmed the origin of Co oxides’ high capacities – the same spin‐polarized surface charge reservoir identified in Fe_3_O_4_, which forms once CoO is fully reduced to metallic Co. Also, the reversible formation of lithium‐containing polymer/gel films at very low voltages, arising from electrolyte decomposition [164]. Specifically, Figure 11i shows the *operando* magnetic response of a p‐n‐CoO lithium‐ion battery under an applied magnetic field of 3 *T*, while Figure 11j presents *operando* magnetic monitoring of a CoO_1‐x_/Co LIB during cyclic voltammetry at 3* T*. Magnetization tracks discerned both the onset of surface electron storage on Co nanoparticles and the separate, more subtle contribution of non‐magnetic polymer film deposition. Notably, the magnetometry data yielded direct evidence that metallic Co plays a catalytic role in forming and decomposing polymeric films, validating a hypothesis that had been proposed to explain extra capacity. *operando* magnetometry can also be used to explore Na‐ion batteries’ inferior capacity and cyclability [165]. FeS_2_ is non‐magnetic, but its conversion to metallic Fe upon full discharge yields a ferromagnetic product, allowing the reaction extent to be inferred from magnetization. By discharging identical FeS_2_ electrodes in Li and Na cells to 0.01 V (at 300 K, Figure 11k) and performing ZFC/FC magnetization measurements at 500 Oe (Figure 11l,m), the magnetization changes reflect partial FeS_2_ conversion to Fe^0^ in Na‐ion cycling, unveiling the unreacted FeS_2_ “inactive cores” persisting inside Na‐treated particles as a key origin of Na‐ion battery's lower attainable capacity. Moreover, Langevin‐fitted magnetization curves indicated that smaller Fe nanoparticle formations in the Na cell (Figure 11n), compared to those in the Li cell, imply more severe pulverization of the active material, correlating with the observed rapid capacity fading in Na‐ion cycles.

In essence, *operando* magnetometry has emerged as a uniquely powerful probe for coupling magnetic, electronic, and ionic degrees of freedom, enabling quantitative deconvolution of complex, multi‐step electrochemical mechanisms that conventional techniques cannot resolve. By directly correlating changes in magnetization with electron accumulation, interfacial space‐charge formation, phase transformations, and conversion completeness, it provides a rigorous metric for where and how charge is stored or lost. Applied broadly – from static magnetoelectric coupling in multiferroics to dynamic magneto‐ionic processes in battery electrodes – *operando* magnetic tracking establishes a unifying framework to read out functional order parameters in situ, clarifying how ferromagnetism and spin polarization interact with ferroelectricity and electrochemistry.

### 4.2 Multiphysics Probes

#### 4.2.1 Ultrafast Optical Probes of Coupled Ferroic Dynamics

**FIGURE 12:**
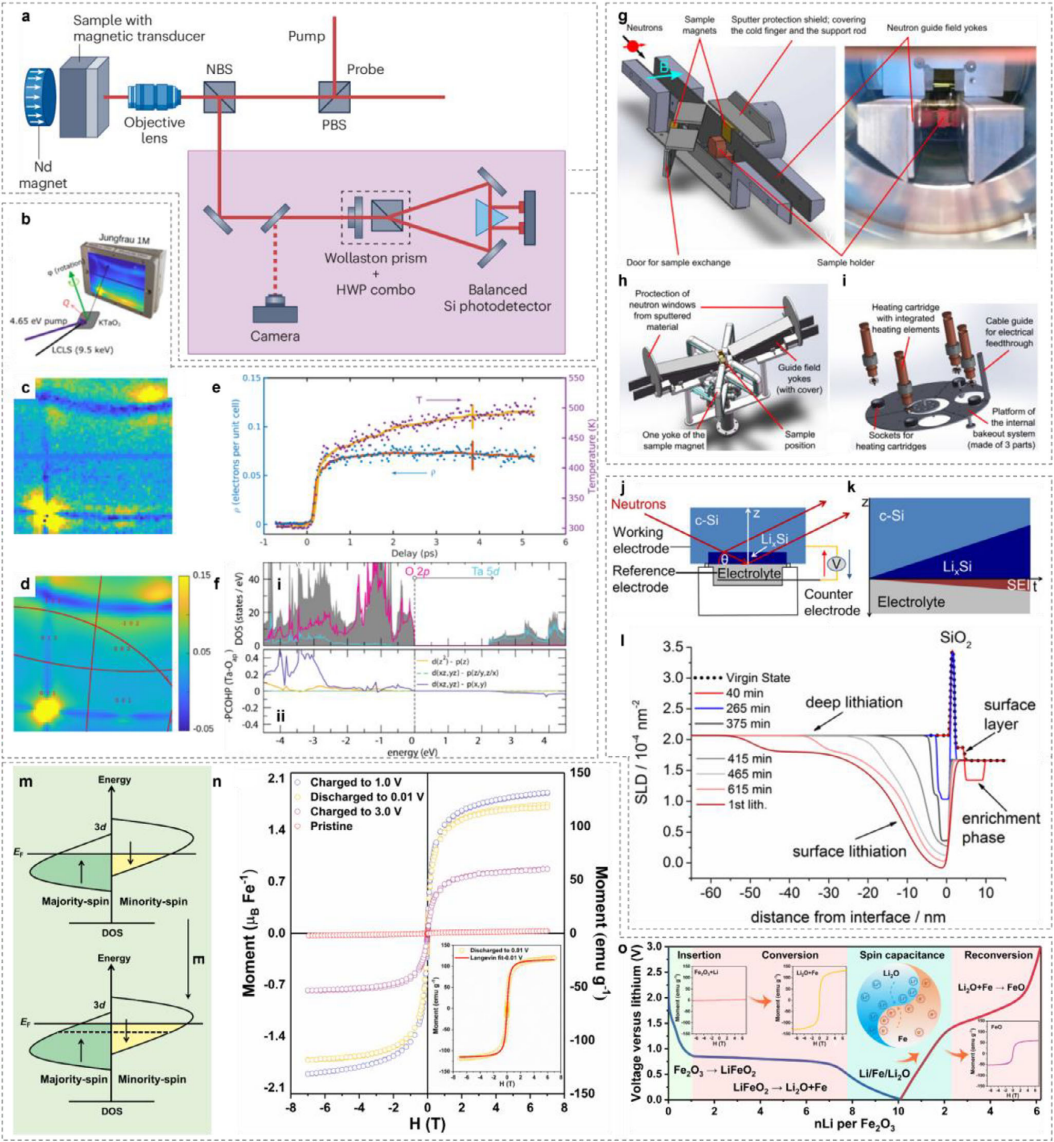
Ultrafast optical/x‐ray methods, neutron reflectometry, and *operando* magnetometry provide time‐ and depth‐resolved access to coupled lattice, magnetic, and electrochemical evolution, enabling direct observation of ferroic dynamics, interfacial lithiation processes, and reversible magnetic responses in working battery‐related systems. (a) Schema for TR‐MOKE experimental setup. Reproduced with permission [167, 168]. Copyright 2018, American Physical Society. (b–f) XFEL probing of KTaO_3_. Reproduced with permission [174]. Copyright 2022, American Physical Society. (b) Schema for XFEL experimental setup. (c) Experimentally measured and (d) fitted Δ*I* (*Q*, *t* = 3 ps). Δ*I* (*Q, t*) in (c) was calculated for 𝜌 = 0.08 electrons per unit cell and *T* = 480 *K* (see text for details). (e) Fitted carrier density *𝜌* and lattice temperature *T* for each time delay as described in the text. The solid lines show the median value within 30 points. (f) (i) Calculated electron DOS and (ii) projected crystal orbital Hamiltonian population (PCOHP) for Ta and the apical O atom. (g–i) in situ and *operando* PNR instrument. Reproduced with permission [178]. Copyright 2020, Elsevier. (g) CAD drawing (left) and photograph (right) of the magnetic field system that is based on permanent magnets. The connection of the sample holder to the cold finger and support rod is hidden from view by the sputter protection shield. (h) Drawing of the magnetic coil system (wires of the coils are not shown) and (i) the upgraded heating system. (j–l) A NR study of crystalline silicon lithiation. Reproduced with permission [180]. Copyright 2016, American Chemical Society. (j) Scheme of the electrochemical cell used for NR studies. A crystalline silicon working electrode is in contact with the electrolyte consisting of 1 M LiClO_4_ in propylene carbonate. The counter electrode consists of metallic lithium. The layer formation of the Li–Si alloy indicated in dark blue is monitored as a function of time by the evaluation of the reflectivity profiles. (k) Scheme of the surface normal, *z*, *vs* time, *t*, at the electrode/electrolyte interface during lithiation. (l) Characteristic scattering length density (SLD) profiles of the virgin state (black dots), taken during lithiation and at the end of first lithiation (dark red) corresponding to the fits to the NR data. After ∼370 min, the native SiO_2_ vanishes and the SLD further decreases with time inside the silicon electrode (dark gray). SLD values between ∼1.80 × 10^−4^ and −0.13 × 10^−4^ nm^−2^ are reached, equivalent to the deep and surface lithiation, respectively. (m–o) An *operando* magnetometry study bridging magnetic order and electrochemistry in battery materials. Reproduced with permission [181]. Copyright 2021, Cell Press. (m) Fully reversible magnetic response of Fe^0^/Li_2_O with respect to the discharging and charging pulses (measured with an applied magnetic field of 7 *T*). (n) *operando* magnetic hysteresis measured at corresponding states with an applied magnetic field of 7 *T*. Inset: Langevin fitting curve of electrode discharged to 0.01 V. (o) Schematic of the number of Li ion insertions/extractions corresponding to initial discharge/charge process. Inset: hysteresis loops of different structures in the transformed state.

Time‐resolved pump‐probe spectroscopy has become a cornerstone for interrogating the fundamental order parameters in multiferroics [166] – polarization, lattice strain, spin, and electronic excitation – and their cross‐coupling on ultrafast timescales [166]. In ferrimagnetic TbFeCo alloys, time‐resolved magneto‐optical Kerr effect (TR‐MOKE, Figure 12a) measurements have revealed an ultrafast demagnetization (∼0.6 ps) followed by a highly damped precessional motion of the magnetization, whose visibility depends sensitively on rare‐earth content and temperature [167, 168, 169]. Integrating into pump‐probe thermoreflectance setups, TR‐MOKE can measure Kerr rotation changes of a magnetic transducer yields superior sensitivity to in‐plane vs. through‐plane thermal transport coefficients, illustrating how magneto‐optical probes can simultaneously capture spin and heat dynamics [167, 168].

Ultrafast optical techniques have also probed ferroelectric order parameters directly via second‐harmonic generation (SHG) [170]. A mid‐infrared pump pulses resonant with a lattice mode in quantum‐paraelectric SrTiO_3_ were used to optically induce a long‐range polar phase, detected by the emergence of a second‐harmonic signal that is forbidden in the centrosymmetric ground state [171]. The appearance of a time‐delay‐independent SHG signal after pump (absent at equilibrium) signified the breaking of inversion symmetry and the onset of ferroelectric order in SrTiO_3_, persisting for hours up to near‐room temperature. Moreover, femtosecond optical pulses were used to manipulate the magnetization in a La_0.7_Ca_0.3_MnO_3_ layer and, via magnetostriction, perturb the ferroelectric polarization in an adjacent Ba_0.1_Sr_0.9_TiO_3_ layer [172]. Time‐resolved SHG selectively probing the Ba_0.1_Sr_0.9_TiO_3_ revealed an ultrafast spin‐lattice relaxation in the manganite, which reduces its spin order and imposes stress on the ferroelectric via the strain‐mediated piezoelectric response. Consequently, the ferroelectric polarization was modified on a timescale far faster than thermal diffusion, demonstrating that elastic coupling can mediate magnetization‐to‐polarization switching on ultrafast timescales [172]. This experiment disentangled the contributions to the SHG signal and showed that the dominant speed limit of magnetoelectric coupling in such a composite is the spin‐lattice demagnetization time in the ferromagnet. Thus, ultrafast optical probes can track polarization or magnetization in real time to identify the rate‐limiting atomic processes (e.g., spin angular momentum transfer or lattice distortion) that ultimately govern electrochemical kinetics.

#### 4.2.2 Advanced X‐Ray Techniques for Multiferroic Lattices

Ultrafast x‐ray techniques provide a complementary window into lattice and orbital dynamics with Ångström‐level spatial resolution. X‐ray free‐electron lasers (XFELs) in particular have enabled angstrom‐wavelength snapshots of structural and charge‐order evolution in photoexcited ferroics on sub‐picosecond timescales [173]. Femtosecond hard X‐ray diffuse scattering was used to capture the phonon dynamics underlying an incipient ferroelectric transition in KTaO_3_ [174]. By probing diffuse scattering between Bragg peaks (Figure 12b,c), the experiment mapped how entire time‐dependent phonon branches and carrier density responded to a 50 *fs* UV laser pulse (Figure 12d). Figure 12e,f reveal a charge‐transfer‐type insulating state in which semicovalent Ta‐O interactions produce an O 2*p*‐derived valence band and Ta 5*d*‐derived conduction band, with photoexcitation promoting electrons across this gap and the ‐pCOHP analysis for the apical O‐Ta bond resolving the corresponding bonding, antibonding, and nonbonding contributions. In essence, XFEL directly measured the photoinduced strengthening of interatomic force constants, providing atomic‐level insight into light‐induced dynamic perturbation of coupled lattice‐charge order. Understanding such non‐equilibrium structural responses could inform strategies to optically tune dielectric properties or phase stability in light‐assisted ferroelectric‐related metal‐air battery.

Time‐resolved X‐ray diffraction (TR‐XRD) and resonant X‐ray scattering are equally powerful for observing structural order parameter changes under external stimuli like electric fields or THz pulses [175]. Applying single‐cycle THz pulses can resonantly excite a soft polar phonon mode (≈1 THz) in Sn_2_P_2_S_6_ at temperatures near its Curie point, while femtosecond x‐ray pulses recorded the resulting structural modulation in real time. The measurements captured the nascent displacive shift of Sn^2+^ ions that underlies the paraelectric‐to‐ferroelectric phase transition, quantitatively tracking the time‐dependent lattice distortion corresponding to the developing ferroelectric polarization [173]. This approach effectively visualizes the ferroelectric soft‐mode dynamics in the time domain, in an electrochemical context, one could use it to monitor how a battery material's crystal structure responds to fast voltage pulses or how magnetostrictive electrode lattice strains couple to magnetic ordering.

More broadly, resonant soft X‐ray scattering (RSXS) at specific absorption edges can selectively probe electronic or magnetic order parameters in multiferroics with element specificity, even under ultrafast excitation [176]. By combining these x‐ray methods with optical pump techniques, researchers can disentangle multi‐component order parameter dynamics – for example, distinguishing lattice‐driven processes from pure electronic excitation. In ultrafast diffraction of Pb(Zr,Ti)O_3_ where pump‐induced changes in selected Bragg reflections allowed the deconvolution of tetragonal and soft‐mode oscillations and their mutual coupling. In multiferroic manganites like TbMnO_3_, time‐resolved resonant X‐ray scattering has even been used to monitor spin‐order (magnetic) reflections, showing that photoexcitation can launch coherent magnons or electromagnons whose dynamics differ markedly between ferroelectric and paraelectric phases. Altogether, the advancement of XFEL and ultrafast X‐ray spectroscopy techniques is furnishing a complete, angstrom‐and‐femtosecond‐scale picture of multiferroic lattice, charge, and spin dynamics. These tools bridge the gap between fundamental ferroelectric/magnetic coupling mechanisms and practical operating conditions – laying the groundwork for designing multiferroic battery materials that exploit ultrafast strain or polarization responses for improved performance.

#### 4.2.3 Neutron Reflectometry and Synchrotron‐Based Techniques

Beyond ultrafast phenomena, powerful neutron‐ and synchrotron‐based techniques, such as in situ and *operando* setups, are critical for examining how coupled multiferroic properties evolve in real working environments. Polarized neutron reflectometry (PNR) and neutron scattering have emerged as powerful techniques for this purpose because of neutrons’ sensitivity to magnetic ordering and light elements (like Li, H) in buried interfaces [177]. The development of a sputter‐deposition chamber integrated directly with a neutron beamline, enabling in situ and *operando* PNR measurements of thin‐film heterostructures as they are grown or actively manipulated [178]. This unique instrument can fabricate magnetic multilayers *in vacuo* while simultaneously monitoring their depth‐resolved nuclear and magnetic profiles with neutrons – all under variable temperature (10–1000 *K*), magnetic field (up to 300 *mT*), and even applied electric bias. The initial trials of this setup demonstrated its fidelity by confirming well‐known thickness‐dependent magnetic properties in Fe layers, and then went further to uncover new interfacial effects (such as emergent DM interactions at a Pd/Fe interface) that would have been elusive without *operando* measurements. This operando PNR approach paves the way to optimize material synthesis and processing conditions to achieve desired ferroic ordering, an approach that could be extended to thin‐film electrode/electrolyte fabrications with multiferroic functionality while immediately assessing their structural and magnetic integrity.

Understanding SEI in Li‐ion batteries has been a critical challenge, and *operando* neutron reflectometry (NR) was used to measure the SEI's formation and growth in a working Li half‐cell [179]. These *operando* measurements enabled sub‐nanometer precision in SEI thickness determination after various cycling protocols, which has been difficult to achieve in the buried, liquid‐solid interface. By directly observing SEI growth in real time, one can correlate electrochemical conditions to interphase thickness – information vital for validating SEI models and improving additives that aim to minimize continuous SEI buildup. Similarly, NR can also provide insight into lithium distribution during electrode reactions. *operando* NR studies of crystalline Si anodes (utilizing a tailored electrochemical cell, Figure 12j,k) during the first lithiation have revealed a two‐stage lithiation mechanism with distinct spatial regions [180]. NR profiles at various times showed a lithium‐rich amorphous layer propagating inward behind a relatively dilute lithiation front (and the disappearance of the native SiO_2_), providing direct evidence for a two‐phase lithiation process in Si (Figure 12l). Such structural data can validate diffusion‐limited lithiation models and guide strategies to mitigate mechanical stress. By quantifying the growth of the lithiated layer in *operando*, NR helps in developing silicon anodes with architectures or prelithiated coatings that promote a more uniform lithiation.

*Operando* magnetometry and synchrotron X‐ray studies have begun to bridge magnetic order and electrochemistry in battery materials, introducing the concept of spin capacitance (schematized as Figure 12m) and demonstrating electrically controlled magnetism by lithium‐ion insertion in a transition metal thin‐film system [181]. Magnetometry performed simultaneously with electrochemical cycling showed a giant, reversible change in magnetization: the ferromagnet's saturation magnetization was modulated by up to ∼0.31 *μ_B_* per metal atom by ionic intercalation, and this switching was surprisingly fast and cyclable. Notably, by driving a deeper lithiation, the study induced a transformation of the surface layer of FeO from antiferromagnetic to ferromagnetic order, as directly evidenced by the appearance of a clear hysteresis loop in *operando* magnetization measurements (Figure 12n). Figure 12o is a schematic diagram of the number of Li ion insertions/extractions in the initial discharge/charge process, and the hysteresis loops at different stages are also reflected in the inset, which realizes giant and fast control of magnetism and demonstrates the effectiveness of *operando* magnetometry verification. The magnetization changes tracked linearly with the CV curves, confirming that ion insertion was the driver of the magnetic modulation.

In summary, these approaches allow researchers to observe transient and in‐depth mechanisms originating from spins, charges, and lattices as they interact, and to follow the gradual “evolution” of those interactions during continuous operation. By leveraging such Multiphysics characterization, we gain not only a fundamental understanding of coupled ferroic mechanisms but also practical guidance for enhancing electrochemical performance. This synergy of techniques stands to accelerate the development of next‐generation energy materials that fully integrate ferroelectric, ferroelastic, and ferromagnetic functionalities for improving battery durability, efficiency, and even multi‐functional energy storage applications.

### 4.3 Advanced Microscopic Techniques

#### 4.3.1 Magnetic Imaging

**FIGURE 13:**
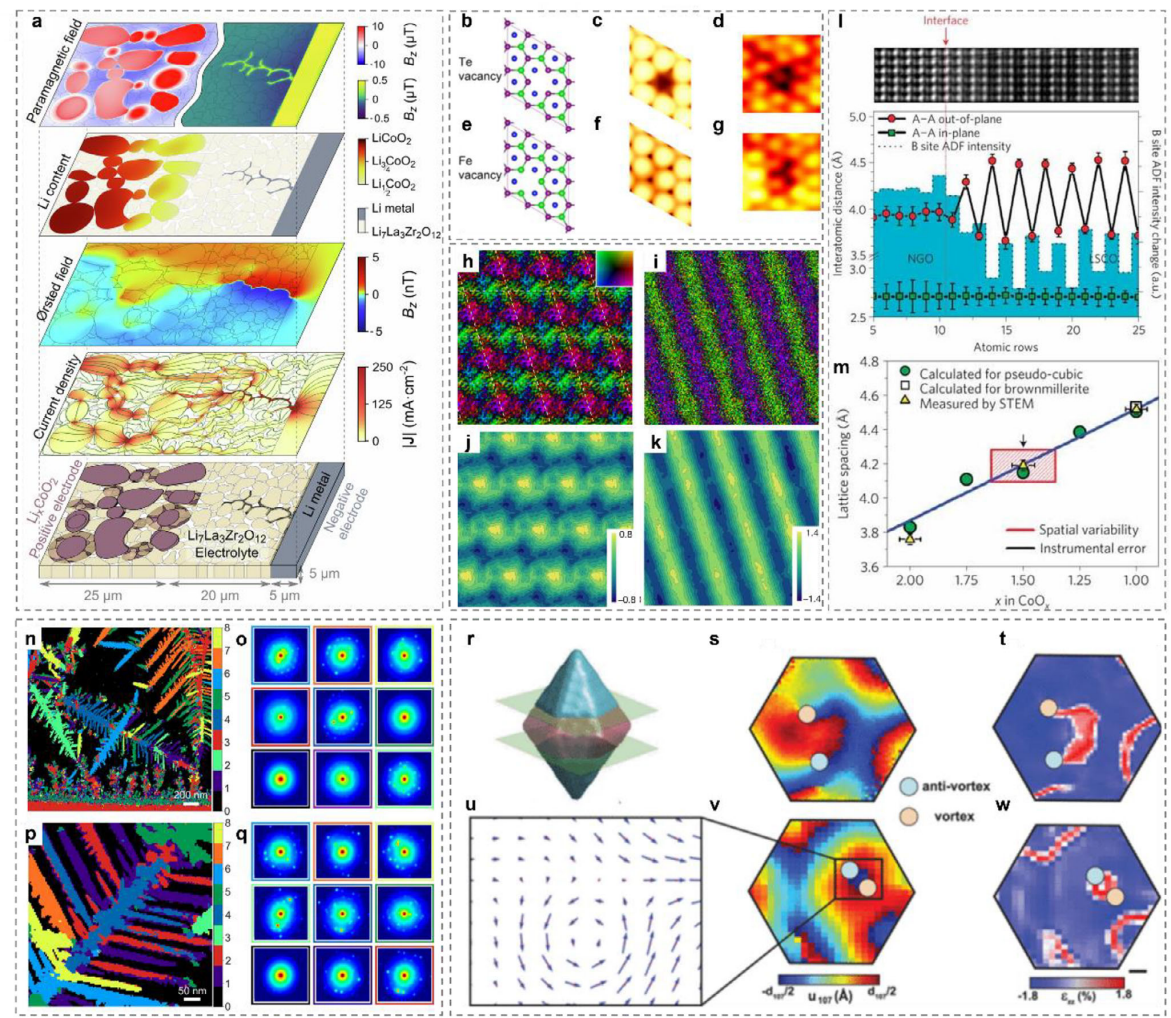
Advanced STEM‐ and scanning‐probe‐based imaging can spatially resolve buried current pathways, dendrites, atomic defects, intrinsic magnetic fields, and sub‐unit‐cell oxygen‐vacancy distributions, thereby linking local structural and ferroic heterogeneity directly to electrochemical transport and failure mechanisms. (a) Finite element simulation of a Li|Li_7_La_3_Zr_2_O_12_|Li_x_CoO_2_ solid‐state battery with the Li_x_CoO_2_ positive electrode particles containing various lithiation states and a lithium metal dendrite forming in the Li_7_La_3_Zr_2_O_12_ electrolyte grain boundaries. Here, both the dendrite and part of the positive electrode particles are buried below the surface, as shown in the lower schematics. In the middle panels, we show the magnitude of the local current density (| J |) with 23.5 mA·cm^−2^ nominal current applied as well as the out‐of‐plane component of the Ørsted magnetic field (*B_z_*) the current produces 50 nm above the surface. In the upper two panels, we show the lithium metal locations and the lithium content of the Li_x_CoO_2_ particles in addition to the magnetic field produced by the paramagnetic components in an external field of 100 *mT*. Reproduced with permission [182]. Copyright 2025, Springer Nature. (b–g) Identification of defects. Reproduced with permission [185]. Copyright 2022, American Physical Society. (b) Top view of the supercell with a Te atom removed. (c) Simulated STM topography of the supercell in (b). (d) STM topography of the Fe_3_GeTe_2_ surface, showing a Te‐site defect. (e) Same supercell, with an Fe atom removed, corresponding to *y*  =  0.14 (i.e., Fe_2.86_GeTe_2_. (f) Simulated STM topography of the supercell in (e). (g) STM topography of the Fe_3_GeTe_2_ surface, showing an Fe‐site defect. STM images (d) and (g) taken with *V*_s_ = 50 mV, *I*_s_ = 50pA. Simulated images (c) and (f) calculated for constant current and *V*_s_ = 100 mV. (h–k) Real‐space visualization of intrinsic magnetic fields via DPC STEM. Reproduced with permission [189]. Copyright 2022, Springer Nature. Projected magnetic field vector color maps observed at (h) room temperature and (i) below *T*_M_. Magnetic phase‐shift images (in mrad) at (j) room temperature and (k) below *T*_M_. (l, m) Probing oxygen vacancy concentration on the subunit‐cell level. Reproduced with permission [97]. Copyright 2012, Springer Nature. (l) Top‐most image is a portion of ADF STEM images of LSCO on NGO. The graphs at the bottom of the image show atomic spacings along the out‐of‐plane (solid circles) and in‐plane (solid squares) directions, averaged over vertical atomic rows of the images. The error bars show the standard deviation with respect to averaging for each (vertical) atomic layer in the image. (m) Lattice expansivity as a function of oxygen deficiency (x in CoO_x_). A linear fit (solid blue line) was obtained from DFT calculations (marked as solid green circles) for five models with different oxygen contents from *x* = 2 (a stoichiometric Co‐O layer in cubic perovskite) to *x* = 1 (for an oxygen‐deficient layer in brownmillerite); variability for LSCO/LSAT layer spacing is due to both instrumental error (black error bars) and intrinsic inhomogeneity (red cross‐hatched box). (n–q) *Operando* 4D‐STEM of Cu dendrites at −40 °C in the organic solution. Reproduced with permission [192]. Copyright 2025, American Chemical Society. (n, p) False‐color 4D‐STEM clustering maps of dendritic and mossy Cu structures, respectively. (o, q) Corresponding electron diffraction patterns, with different colors indicating different crystal orientations. (r–w) Reproduced with permission [193]. Copyright 2025, AIP Publishing. Topological analysis of a barium hexaferrite particle. (r) Isosurface rendering of the reconstructed particle, with the analyzed slices indicated. (s, t) Displacement and strain maps, respectively, from one selected slice, with anti‐vortices highlighted. (u) Polarization‐field map from a representative region of the reconstruction. (v, w) Displacement and strain maps from a second slice, showing the coexistence of vortex and anti‐vortex structures. Scale bars in (s, t, v, w) are 50 nm.

Magnetic imaging techniques provide nanoscale insight into ferromagnetism (*M*) and coupled ionic/electronic dynamics in battery‐relevant materials. Scanning nitrogen‐vacancy (NV) center magnetometry can quantitatively map stray magnetic fields from currents and magnetization in *operando*. Finite element simulations for a solid‐state Li|Li_7_La_3_Zr_2_O_12_|Li_x_CoO_2_ battery predict ∼10 µ*T* stray fields at 50 nm above the electrode during cycling, within NV detection limits (Figure 13a) [182]. This enables Ørsted‐field imaging of buried ionic currents and changes in magnetic susceptibility as transition‐metal redox states evolve, also inhomogeneous charge flow visualization and redox reactions in working batteries, addressing the nanoscale heterogeneities linked to degradation [183]. NV magnetometry thus links local *M* changes to electrochemical state‐of‐charge and can pinpoint “hotspots” of activity impacting battery performance [182]. In conclusion, magnetic imaging and particularly NV magnetometry may find diverse uses as a new probe for correlated and non‐correlated visualization of functional processes in lithium and post‐lithium batteries. These methods can bring new insights into redox reactions at the positive and negative electrodes and degradation pathways while probing the effect on battery charge dynamics and current distributions.

At atomic scales, spin‐polarized scanning tunneling microscopy (SP‐STM) directly resolves ferromagnetic domain structures [184]. In the 2D van der Waals ferromagnet Fe_3_GeTe_2_, SP‐STM imaging revealed domain walls only ∼2.6 nm wide – far thinner than in typical 2D ferromagnets (10‐100 nm) and closely reflects the high anisotropy and low exchange coupling of the surface layer [185]. By comparing the SP‐STM domain wall profile with magnon dispersion data, the wall width matched that expected from bulk Fe_3_GeTe_2_’s exchange (*J*) and anisotropy (*K*), confirming quantitative consistency between reAl–Space STM and reciprocAl–Space neutron scattering. Notably, the surface domain wall was narrower than the bulk prediction (∼6 nm), indicating enhanced surface magnetic anisotropy and modified exchange – an important factor for 2D spintronic devices. SP‐STM also captures how defects pin magnetic scattering and domain walls at the atomic scale (Figure 13b–g) [185]. Such reAl–Space magnetic measurements (*M*) enable direct correlation of domain morphology with magnetic exchange parameters and can guide the tuning of 2D magnets for stable information storage.

Beyond SP‐STM, functionalized magnetic tips extend scanning tunneling microscopy to probe local magnetism even without external fields. Using a NiCp_2_ molecule on an STM tip as a sensor, researchers identified nanoscale ferromagnetism in monolayer transition‐metal dichlorides [186]. In FeCl_2_, the NiCp_2_‐functional tip detected an exchange bias of 0.17 meV at zero field, evidencing an out‐of‐plane ferromagnetic order that persists without an applied field. In contrast, monolayer NiCl_2_ (easy‐plane anisotropy) showed no splitting in zero field, consistent with in‐plane magnetization undetectable by the out‐of‐plane tip sensor. These measurements, enabled by inelastic electron tunneling spectroscopy (IETS) at 4 K, confirm that FeCl_2_ retains perpendicular ferromagnetic domains down to one layer while NiCl_2_’s magnetization lies in‐plane. By tracking the NiCp_2_ signal versus tip height, the local stray field from FeCl_2_ was found to decay within ∼80 pm, highlighting the nanometric confinement of the ferromagnetic region [186]. This technique maps local exchange fields (*M*) with sub‐nanometer resolution, and importantly, the ability to write or toggle magnetic states via tip‐induced strain (magnetoelastic coupling) has been demonstrated in related systems [187].

Advanced magnetic microscopies – from NV sensors to spin‐resolved STM – provide quantitative, *operando* access to multiferroic magnetic order. The resulting observations of domain size, wall width, and current‐induced fields link the *M* parameter to underlying exchange anisotropy and electrochemical processes, informing strategies to optimize battery electrodes and spintronic materials via magnetic homogeneity. Magnetic microscopies map domain structures and stray fields to extract *J* and *K* (hence *M*), enabling correlation to electrochemical current distribution and redox heterogeneity that govern overpotential and degradation.

#### 4.3.2 STEM and X‐Ray Imaging

High‐resolution STEM techniques identify lattice distortions and visualize ferroelectric (P) and ferroelastic (ε) order parameters with atomic to nanoscale precision [188], even in active environments. Differential phase contrast (DPC) STEM has now resolved intrinsic magnetic and electric fields inside the lattice – a recent breakthrough achieved real–space mapping of the tiny antiferromagnetic fields in *α*‐Fe2O3 by atomic‐resolution DPC and annular dark‐field (ADF) STEM [189]. By operating the microscope in zero‐magnetic‐field mode and using segmented detectors, researchers differentiated the magnetic phase shift from the overwhelming electric phase shift of Fe2O3's lattice (Figure 13h‐k). The measured DPC contrast varied with the Morin transition temperature (TM ∼260 K) [189, 190], confirming that the image contrast indeed arose from the intrinsic spin orientation. This experiment demonstrated that STEM can directly image spin arrangements coupled to the crystal structure at sub‐Å resolution [189]. Such capabilities pave the way to mapping antiferromagnetic or toroidal order in multiferroics, correlating atomic‐scale spin textures with lattice distortions and temperature‐dependent transitions.

STEM can also reveal compositional defects that couple to ferroic properties. In oxide cathodes (e.g., La_0.5_Sr_0.5_CoO_3‐x_), STEM‐based lattice spacing mapping enabled quantitative oxygen vacancy imaging [97]. Local lattice expansion due to O‐vacancies was measured at the sub‐unit‐cell level, and using the concept of chemical expansivity, converted to vacancy concentration maps. Applying this to La‐Sr‐CoO_3_ films on different substrates uncovered distinctly more vacancies in films on NdGaO_3_ compared to LSAT, explaining a measured difference in their magnetization (Figure 13l) and interplanar spacing difference (Figure 13m). Polarized NR confirmed that the film with more oxygen vacancies had a greater saturation magnetization. Thus, STEM pinpointed how local non‐stoichiometry (oxygen vacancies) drives variations in multiferroic magnetization – a critical insight for solid‐oxide fuel cell cathodes and memristors where anionic defects modulate electronic/magnetic behavior. More broadly, this approach links lattice strain to functional properties, suggesting that tuning oxygen chemical potential during synthesis can control a material's magnetic or ferroelectric state.

*Operando* and in situ STEM analysis under stimuli, such as electric bias, gas environment, or temperature, is crucial for electrochemical materials. In situ temperature‐dependent STEM on free‐standing BaTiO_3_ films has shown that the ferroelectric domain structure is extremely sensitive to atmosphere [191]. In a vacuum, BaTiO_3_ exhibited a rich pattern of ferroelectric‐ferroelastic (multiferroic) domains and underwent a well‐defined phase transition on heating. Under an oxygen‐rich atmosphere, however, domain walls largely vanished (suppressed domain structure), and the ferroelectric transition temperature shifted. Notably, a peculiar reconfiguration of twin domains from an a‐c pattern to a‐a occurred at ∼60°C in vacuum, whereas in oxygen this rearrangement did not manifest [191]. This direct in situ observation illustrates that surface oxygen species adsorbates effectively screen polarization charge, stabilizing or destabilizing certain domain states. Thus, the chemical environment controls ferroelectric phase stability – a vital consideration for battery oxides and catalysts where surfaces are exposed. By combining gas dosing with automated image analysis (e.g., deep learning to detect domain changes), such studies map out how *P*‐*ε* coupling in ferroelectrics responds to realistic operating conditions, guiding the design of multiferroic interfaces to maintain polarization at desired temperatures or atmospheres.

4D‐STEM and three‐electrode electrochemical liquid‐cell STEM further enable real‐time visualization of structural evolution and heterogeneous kinetics during electrochemical reactions. Using an environmental liquid cell, Kim et al. applied *operando* 4D‐STEM to Cu electrodeposition [192]. By clustering the high‐dimensional 4D‐STEM dataset, they identified the initial formation of nanometer‐scale, randomly oriented Cu islands, followed by the growth of oriented, micron‐scale dendritic Cu filaments (Figure 16n‐q). In other words, slower diffusion‐limited deposition produces fine polycrystalline deposits, whereas faster deposition promotes oriented large‐crystal dendrites, consistent with local electric‐field intensification at dendrite tips [192]. This nanoscale observation explains why low‐temperature or high‐rate battery charging can promote dendritic growth and compromise electrochemical stability. *Operando* 4D‐STEM therefore connects structural anisotropy and grain dynamics to electrochemical conditions, informing strategies to suppress dendritic failure by controlling deposition kinetics.

Finally, Bragg coherent diffraction imaging (BCDI) uses coherent X‐rays to map 3D strain and polarization distributions inside crystals, enabling visualization of complex ferroelastic domain morphologies in single ferroelectric nanoparticles with ∼10–20 nm resolution [193]. The reconstructed particle morphology and analyzed internal slices can be visualized by isosurface rendering, providing the structural basis for locating domain and topological features (Figure 16r). From selected slices, BCDI resolves displacement and strain fields, revealing anti‐vortex features and local ferroelastic distortions within the particle (Figure 16s,t). The reconstructed polarization‐field map further captures the spatial rotation of polarization vectors, enabling identification of topological polarization textures such as vortex and anti‐vortex configurations (Figure 16u). In another slice, the coexistence of vortex and anti‐vortex structures is directly correlated with the corresponding displacement and strain distributions, illustrating how polarization topology couples to ferroelastic strain (Figure 16v,w). These x‐ray techniques uniquely capture internal strain fields and defect evolution in working or field‐stimulated materials, complementing electron microscopy. As detectors and coherent X‐ray sources continue to improve, BCDI is expected to resolve smaller domains and faster dynamics. Collectively, advanced STEM and X‐ray imaging provide a multifaceted view of multiferroic materials, from atomic polarization and defect maps to *operando* domain switching under realistic environments. This knowledge links ferroelectric and ferroelastic parameters to material performance, including the stabilization of ferroelectric order by surface chemistry and the coupling of strain accumulation to magnetic and electronic changes.

#### 4.3.3 Scanning Probe Microscopy and Polarized Light Microscopy

**FIGURE 14:**
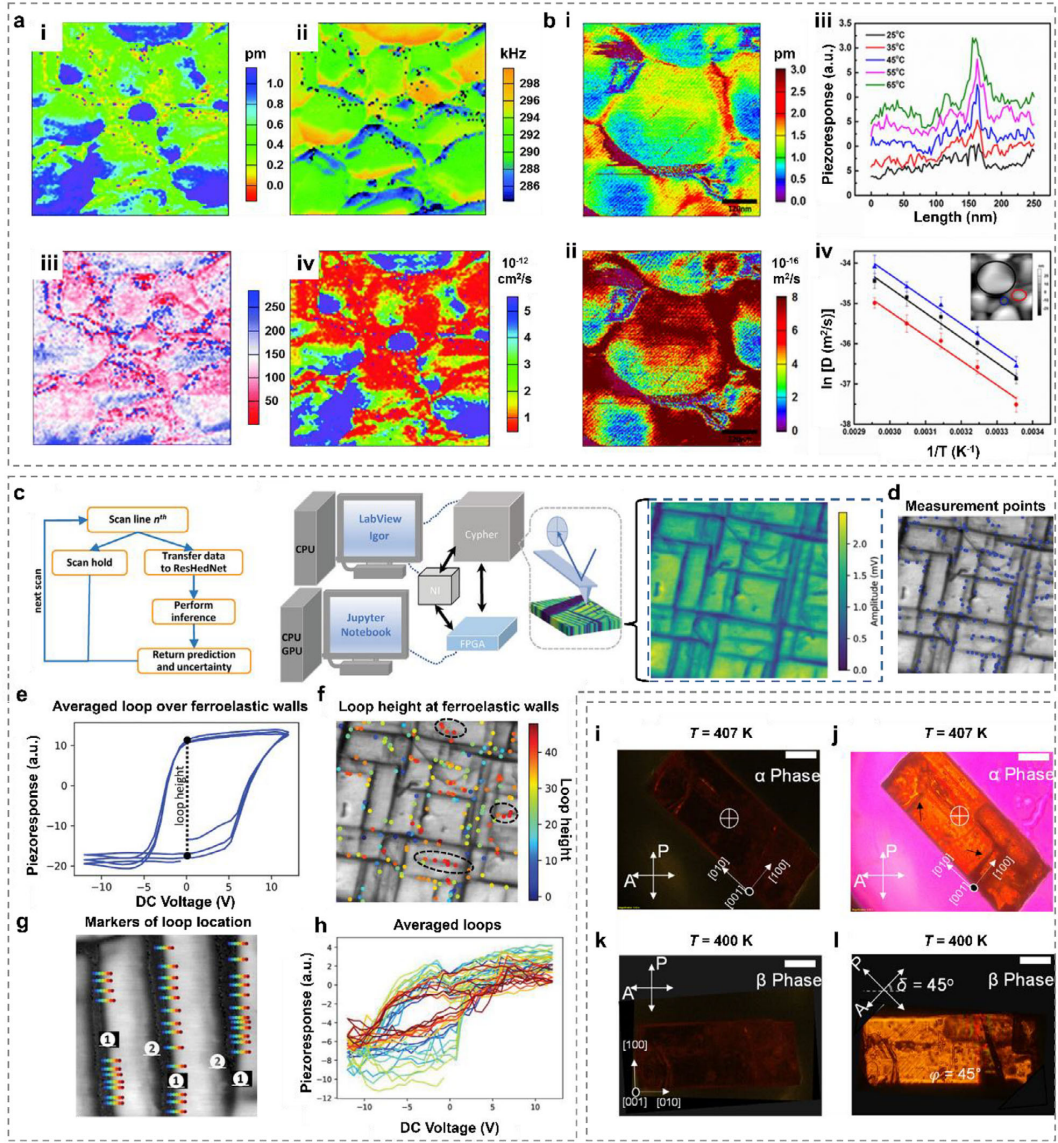
SPM and PLM can directly visualize ion‐transport heterogeneity, quantify local diffusion and activation barriers, resolve ferroelastic domain‐wall switching behavior, and identify ferroelastic domain patterns optically, thereby linking microscopic ferroic structure to transport and switching functionality in battery‐relevant materials. (a) BE‐ESM images (with 3 V_ac_ bias) of Li‐rich cathode film: (i–iv) Diffusion coefficient mapping. Reproduced with permission [194]. Copyright 2015, The Royal Society of Chemistry. (i) resonance frequency; (ii) resonance amplitude; (iii) Q‐factor images; (iv) calculated diffusion coefficient map; (b) in situ ESM for probing activation energy: (i) DRAT‐ESM amplitude image (600 × 600 nm^2^) measured at 55°C; (ii) calculated diffusion coefficient map from image (i), (iii) amplitude profile at selected line with increasing temperatures from 25 to 65°C, and (iv) Arrhenius plot of the diffusion coefficients as a function of the inverse of temperature for selected grains. Reproduced with permission [197]. Copyright 2017, American Chemical Society. (c–h) BEPFM for probing domain wall characteristics. Reproduced with permission [195]. Copyright 2022, John Wiley and Sons. (c) The workflow and integrated system for the real‐time ferroelastic wall investigation. The inset is BEPFM amplitude image of the PZT; (d) EPS measurement points on ferroelastic domain walls; (e) Averaged piezoresponse versus voltage loops over all measurement points, and schematically shows how the loop width is extracted; (f) Loop width over the ferroelastic domain walls, where the color represents loop width; (g) and (h) Averaged loops at locations near domain wall <1> with a certain distance from the domain walls. (i–l) PLM images of a (001)‐oriented CsPbBr3 crystal plate. Reproduced with permission [196]. Copyright 2023, American Chemical Society. (i) and (j) The crystal in the α phase at an arbitrary position under crossed polarizers with (j) and without (i) a superimposed first‐order red plate. (k) and (l) The same crystal in the β phase when the crossed polarizers are at δ = 0/90° (k) and δ = 45° (l).

SPM and PLM offer versatile, high‐resolution characterization of ferroelectric (*P*) and ferroelastic (*ε*) behaviors, often under functional conditions. Electrochemical strain microscopy (ESM) and conductive atomic force microscopy (AFM) allow complementary mapping of ionic mobility and conductivity. Li and Zeng showed that multifield SPM techniques can probe local coupling phenomena in energy materials. Band‐excitation ESM (BE‐ESM) on a Li‐rich cathode film (Li_1.2_Co_0.13_Ni_0.13_Mn_0.52_O_2_) was used to extract the local Li^+^ diffusion coefficient by fitting the vibration amplitude response (Figure 14a) [183, 194]. The SPM‐derived diffusion constant (∼10^−13^–10^−12^ cm^2^ s^−1^) matched the order of magnitude from bulk electrochemical tests [183], while temperature‐dependent ESM determined the activation energy for Li‐ion hopping (Figure 14b). These nanoscale measurements reveal spatial variations, such as slower diffusion near grain boundaries, that conventional methods average out. Similarly, in *operando* ESM mapping of LiNi_0.8_Mn_0.1_Co_0.1_O_2_ battery has identified regions of higher lithium intercalation strain correlating with crack initiation. By linking ionic diffusion to local strain and ultimately to electrochemical performance, SPM provides a multi‐physics view of battery electrode heterogeneity. This informs how *P*‐*ε* multiferroic coupling and ionic transport interplay at the nanoscale, guiding improvements in rate capability and cycle life through microstructure engineering.

Piezoresponse force microscopy (PFM), a contact‐mode SPM, directly images nanoscale ferroelectric domains via their electromechanical response. Importantly, PFM can be performed in controlled atmospheres to decouple electrostatic and electrochemical effects [63]. Using environmental PFM on hafnium oxide (HfO_2_) films – a binary oxide ferroelectric – researchers demonstrated that surface electrochemical state governs ferroelectricity. In ambient air, HfO_2_ showed pinched P‐E loops indicative of antiferroelectric‐like behavior. When measured in an inert Ar glovebox, the loops opened slightly but still lacked remanence, behaving as a paraelectric with negligible retained polarization. In ultrahigh vacuum, the same film exhibited clear ferroelectric switching with a wide hysteresis loop and non‐zero remanent polarization. Furthermore, temperature‐dependent PFM in different atmospheres showed that the critical transition temperature and loop shape shift with ambient chemistry.

Mechanical SPM can also deliberately manipulate ferroic domains. Scanning probe‐induced strain was recently harnessed to “write” ferroelastic domain patterns on demand. Peng et al. utilized an AFM tip to apply shear stress and reorient crystal domains in epitaxial oxide films [187]. By raster‐scanning under specific load and direction, they converted multi‐variant twin domains in a SrRuO_3_ film into a single‐domain state, effectively creating a local single crystal orientation. The AFM‐written domains were stable and could be erased or rewritten reversibly, demonstrating deterministic control of a ferroelastic order parameter by pure mechanical force. On the ferroelectric side, advanced SPM protocols with deep learning now enable real‐time tracking of domain wall motion and local switching behaviors. For instance, a deep neural network integrated with band excitation PFM (BEPFM, Figure 14c) was used to automatically identify active domain wall regions during biasing and then perform site‐specific spectroscopy on those walls [195]. This workflow (Figure 14c‐h) discovered that certain domain walls in PbTiO_3_ exhibit an “alternating” polarization switching response (periodic high vs. low switchability), and that in Pb(Zr, Ti)O_3_ films, short ferroelastic walls show significantly enhanced polarization dynamics compared to long walls (Figure 14g,h). These findings imply that domain wall length and configuration can influence local ferroelectric switching kinetics, which could be exploited in domain‐wall‐mediated charge transfer.

PLM, while limited to microscale resolution, powerfully identifies ferroelastic domain patterns via birefringence. PLM confirms an optically isotropic cubic α phase at 407 *K* (complete extinction at all *δ*, Figure 14i), uses a first‐order red plate to distinguish fixed growth steps and inclusions from genuine ferroic contrast (Figure 14j), and, upon cooling below ∼403 *K*, reveals extinction at *δ* = 0°/90° (Figure 14k) together with fine lamellar twins aligned along [131] (Figure 14l), evidencing the emergence of a ferroelastic/ferroelectric *β* phase with 90°‐type domain walls. Using PLM under applied stress, studies proved the ferroelastic nature of domain walls in halide perovskite CsPbBr_3_ [196]. In a single crystal plate viewed between cross‐polarizers, no domain pattern is seen initially, reflecting CsPbBr_3_’s ferroelastic behavior, with a mechanical threshold for domain reorientation. Such PLM observations link directly to the *ε* (strain) order parameter, and the ability to visibly distinguish ferroelastic twins and follow their motion under stimuli (stress, *E*‐field, temperature) makes PLM a convenient tool for screening multiferroics and for verifying mechanical switching pathways that SPM then quantifies at the nanoscale.

Advancing multiferroics for battery applications faces a key bottleneck: conventional electrochemical characterizations provide limited visibility into how ferroic order parameters govern performance, obscuring mechanistic understanding and slowing rational design. This section addresses that gap by outlining a more rigorous characterization and analysis framework in which SPM/PLM observables – local hysteresis loops, ionic‐strain maps, ferroelastic twin patterns, and domain‐wall dynamics – are quantitatively correlated with electrochemical metrics such as diffusion coefficients, overpotentials, capacity retention, and crack‐initiation loci.

### 4.4 Complementary Spectroscopy

#### 4.4.1 THz Spectroscopy

**FIGURE 15:**
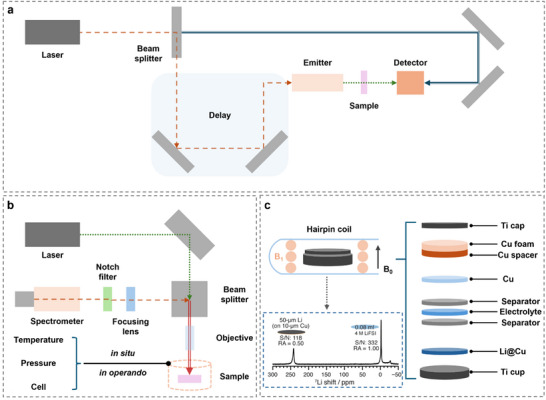
Experimental configurations for multimodal *operando* spectroscopy. (a) THz‐TDS setup illustrating femtosecond laser generation and detection of THz pulses, beam steering and delay control, and transmission through the sample positioned within the optical path. (b) in situ/*operando* Raman spectroscopy configuration, showing laser excitation, optical collection geometry, and a custom electrochemical cell enabling real‐time structural interrogation under applied bias. Reproduced with permission [202]. Copyright 2024, American Physical Society. (c) *operando* ^7^Li solid‐state NMR configuration, including the electrochemical cell integrated within the NMR probe, lithium‐metal symmetric cell architecture, and representative spectral acquisition for tracking Li environments during cycling. Together, these complementary platforms enable simultaneous access to lattice dynamics, local structural evolution, and Li‐ion transport processes under realistic operating conditions. Reproduced with permission [205]. Copyright 2021, American Association for the Advancement of Science.

Terahertz‐frequency spectroscopies provide powerful insights into low‐energy excitations such as phonons and magnons that underpin multiferroic behavior. In multiferroic BiFeO_3_, for example, THz/far‐infrared (THz‐FIR) measurements revealed that the apparent magnetodielectric coupling above ∼200 *K* is dominated by magnetoresistive effects rather than a true polarization‐magnetization interaction. Several polar phonons exhibit only incomplete softening on heating toward the ferroelectric transition, consistent with a first‐order character [198]. Concurrently, THz time‐domain spectroscopy (THz‐TDS, Figure 15a) of ferroelectric GeTe detected no critical soft‐mode behavior in the paraelectric phase but revealed a new low‐frequency relaxation, supporting an order‐disorder component to the transition [199]. THz spectroscopy can also capture coupled spin‐lattice excitations in magnetoelectric materials. In Ba_2_Mg_2_Fe_12_O_22_, THz optical spectra under a magnetic field, together with polarized inelastic neutron scattering, identified an electric‐dipole‐active magnon near 5 meV and demonstrated oscillating electric polarization along the *c*‐axis, evidencing dynamic spin‐polarization coupling (electromagnon) [200]. Moreover, terahertz‐frequency magnons at ferromagnetic surfaces have lifetimes of only tens of femtoseconds and propagate merely a few nanometers before damping, highlighting the fundamental speed limits of magnetization switching and energy dissipation in spin‐based devices [201]. While THz spectroscopy is less common in battery research, its ability to probe ultrafast ionic motions and localized dielectric responses can complement electrochemistry by informing on facile ion migration, field‐driven phase transitions, and dissipative pathways in solid electrolytes and electrodes.

#### 4.4.2 Raman Spectroscopy

Raman scattering probes vibrational modes that are exquisitely sensitive to crystal symmetry, bonding, and local strain, making it a versatile tool for diagnosing ferroic order, phase transitions, and domain‐wall physics. In the van der Waals multiferroic CuCrP_2_S_6_, in situ high‐temperature/high‐pressure Raman spectroscopy (Figure 15b) combined with electrical conductivity pinpointed the ferroelectric Curie transition at ∼423 *K* under ∼0.4 GPa and charted pressure‐driven structural/electronic changes, thereby establishing a direct spectroscopic link between lattice symmetry breaking and transport anomalies [202]. Under more extreme conditions, high‐pressure Raman/x‐ray studies on multiferroic BaFe_4_O_7_ evidenced a pressure‐induced charge transfer and an irreversible order‐disorder structural state upon decompression, with corroborating Fe‐edge XAS/extended x‐ray absorption fine structure (EXAFS) signatures; the persistence of the disordered motif at ambient conditions rationalizes pressure‐tunable ferroic/electronic responses relevant to functional property optimization [203]. Raman techniques also enable local thermophysical metrology: an optothermal Raman study on BiFeO_3_ mapped domain‐wall–mediated phonon scattering, showing that ferroelastic domain walls reduce thermal conductivity by ∼46% at room temperature (e.g., from ∼2.17 to 1.16 W m^−1^ K^−1^), with implications for heat management and electro‐thermal coupling in devices [204]. In battery systems, operando Raman tracks structural signatures of (de)intercalation (e.g., graphite staging via G‐band evolution), conversion/intermediate formation, and stress/strain evolution – allowing reaction pathways and kinetics to be correlated with polarization loss, impedance growth, and capacity fade.

#### 4.4.3 Nuclear Magnetic Resonance (NMR) Spectroscopy

NMR is a sensitive local probe of atomic‐scale environments, capable of detecting internal magnetic fields and electric field gradients via chemical shifts, Knight shifts, and quadrupolar couplings. In multiferroics, NMR can quantify order parameters and domain distributions – distinguishing ferroelectric domain orientations through their distinct hyperfine fields, thereby locally assessing coupling between magnetic and electric order. For batteries, *in operando* NMR (Figure 15c) provides mechanistic visibility inside a working cell without disassembly. A high‐field in situ study demonstrated that, by using B_1_‐field “skimming” geometries, unmodified commercial coin cells with metal casings can be probed to resolve distinct ^7^Li signals from metallic Li, β‐LiAl alloy at the anode, and Li_x_MnO_2_ in the cathode; correlated changes in the *β*‐LiAl Knight shift and *T*_2_′ during cycling reported defect‐structure evolution tied to lithiation state [205]. These capabilities allow NMR to directly track lithium plating/alloying, ion mobility, and local redox chemistry – providing a bridge from ferroic microstructure (e.g., domain‐wall‐mediated strain fields affecting local diffusion) to rate capability, reversibility, and degradation in batteries.

#### 4.4.4 Synchrotron Radiation XAS

Synchrotron‐based XAS (X‐ray absorption near edge structure (XANES)/EXAFS) provides element‐specific access to local electronic structure (valence) and coordination (bond lengths, disorder), which are central to both multiferroics and battery electrodes. In multiferroic BiFeO_3_‐BaTiO_3_ ceramics, Cu/Mn *K*‐edge XANES/EXAFS unambiguously located both dopants on perovskite B‐sites (Cu at Fe‐site, Mn at Ti‐site) and confirmed their valence states, establishing how targeted substitution perturbs local symmetry and electronic structure to tune ferroic responses [206]. In batteries, in situ XAS at transition‐metal edges quantifies redox state changes and monitors local coordination during cycling; tracking edge shifts and EXAFS shell distances reveal phase transitions, cation migration, and amorphization that underpin voltage hysteresis and capacity fade. Together with THz, NMR, and Raman, XAS thus closes the loop from local bonding and valence to ferroic domain behavior and electrochemical performance, offering a comprehensive, multi‐modal toolkit for diagnosing and engineering multiferroic battery materials.

## 5 Conclusions and Outlook

**FIGURE 16:**
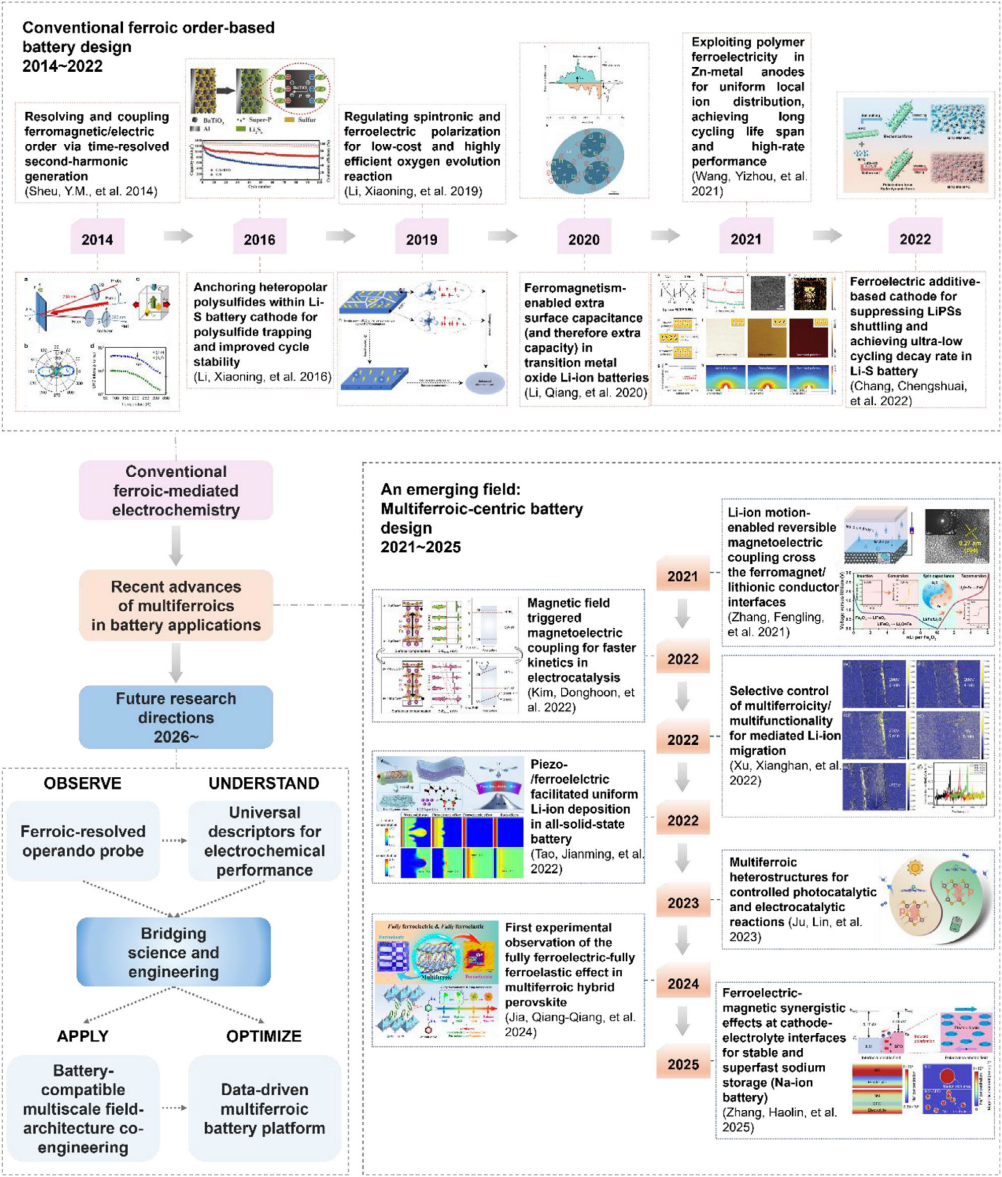
Evolution of ferroic‐mediated electrochemistry toward multiferroic‐centric battery design and future research priorities. The upper panel outlines the historical progression from representative studies of conventional ferroic‐mediated electrochemical regulation to the more recent emergence of explicitly multiferroic concepts in battery‐related systems, arranged chronologically to highlight how the field has evolved from isolated ferroic effects toward coupled polarization–magnetization–strain functionality (∼2014–2022) [76, 77, 78, 89, 141, 172]. The right panel maps representative recent advances (∼2021–2025) onto this emerging framework, illustrating how progress in coupled multiferroic mechanisms, interface regulation, adaptive transport control, and field‐responsive architectures is beginning to define a coherent path toward scalable, mechanistically grounded multiferroic‐enabled battery technologies [9, 59, 71, 116, 117, 181, 207]. The lower‐left panel distills the key research priorities identified herein: establishing ferroic‐resolved *operando* probes and universal electrochemical descriptors to bridge mechanistic understanding with practical engineering, and advancing battery‐compatible multiscale field‐architecture co‐engineering together with data‐driven multiferroic battery platforms (∼2026‐future). Together, these timelines define the aim and scope of this review and connect fundamental scientific understanding with practical materials fabrication. The middle‐left panel summarizes this transition conceptually, showing the shift from conventional ferroic‐assisted electrochemistry to recent multiferroic battery studies and, ultimately, to the future directions proposed in this review. Overall, this figure serves as a roadmap linking the field's historical development, current breakthroughs, and the most critical next steps required to translate multiferroicity from proof‐of‐concept studies into practical battery design principles.

In this review, we have systematically discussed various ferroic‐induced phenomena in material sciences and their transformative impacts across different types of batteries such as ASSB, Li–Sulfur, and metal‐air batteries (Figure 16). The underlying mechanisms by which the material multiferroic properties can be coupled and tailored to enhance corresponding performance and enrich the functionalities are thoroughly revealed. By mechanistically decomposing multiferroicity into ferroelectric, ferromagnetic, and ferroelastic contributions, and mapping each to concrete electrochemical functions – built‐in fields for space‐charge collapse and metal deposition control, spin polarization for multi‐electron electrocatalysis, and ferroelastic strain/twin architectures for chemo‐mechanical toughening and ion‐pathway guidance – we bridge a critical gap between qualitative “multiferroic effects” and quantitatively targetable battery performance metrics such as ASR, CCD, interphase stability, and fracture resistance. Beyond mechanistic insights, this work provided an explicit and transferrable framework to tune multiferroicity for battery applications, organizing materials strategies along three tightly coupled axes: (i) multiphysics field control (electric, magnetic, mechanical, optical) to tailor polarization, spin, and strain landscapes in *operando*; (ii) mesoscale and 3D architectural design that embed internal biases and gradients where charge and ion transfer are rate‐limiting; and (iii) ferroic‐centric defect engineering that leverage vacancies, elastic dipoles, chemopiezoelectricity, and magneto‐ionic effects to render ferroic responses robust under high voltage, strong redox, and solid‐solid interfaces. In parallel, we rationally integrated magnetometry, advanced microscopy (STEM/SPM/PLM), neutron‐ and synchrotron‐based probes, ultrafast optics, X‐ray techniques, and complementary techniques, to directly correlate *P*/*M*/*ε* dynamics with interfacial kinetics, degradation modes, and failure.

Despite these advances, key gaps remain (Figure 16): robust *operando* control of external and built‐in fields and pre‐conditioning protocols for coupled order parameters under realistic operating conditions; scalable imprinting and retention of targeted polarization, magnetization, and twin topology across electrodes, electrolytes, and interphases; standardized descriptors that quantitatively link multiferroic states to electrochemical metrics; and system‐specific validation frameworks that avoid uncontrolled extrapolation across halide, oxide, and sulfide chemistries; and, critically, manufacturing‐compatible and cost‐effective routes that translate multiferroic functionality from epitaxial or defect‐sensitive model systems into thick‐electrode, large‐area, and high‐throughput battery fabrication. Targeting these pain points, we have proposed four potential research directions for scientists to implement multiferroic concepts to engineer batteries:

1. Ferroic‐resolved *operando* probe for multiferroic‐induced electrochemical reaction variations: Establish an integrated *operando*, multimodal, and multiscale analyzing framework that quantitatively maps polarization (*P*), magnetization (*M*), and ferroelastic strain (*ε*) at buried interfaces in cells and compresses them into standardized descriptors of ASR, CCD, ion diffusivity, interphase evolution, and fracture toughness. Apart from *operando* characterizations, in situ setups are critical to discover the intricate materials’ properties, especially the cryogenic temperature range has been underutilized, yet it is essential for probing ferromagnetic‐/electromagnetic‐centric phenomena. Thus, advanced and tailored characterizations are the key to turn multiferroicity from a qualitative narrative into a rigorous, causal design variable for battery performance.
2. Universal descriptors: Harness spin‐selection rules, magneto‐ionic migration, chemopiezoelectric responses, and ferroelastic/ferroelectric switching to create dynamically reconfigurable multiferroic units whose *P*‐*M*‐*ε* landscapes self‐adjust during cycling to buffer chemo‐mechanical stress, suppress dendrite‐ and crack‐mediated degradation, and accelerate interfacial charge transfer and multi‐electron electrocatalysis in a physically grounded, reversible manner.
3. Battery‐compatible multiscale field‐architecture co‐engineering: Integrating 3D structural modifications, via controlled core‐shell architectures, amorphous/crystalline dual phases, heterojunctions, textured grains, and engineered twin/domain‐wall networks, with both built‐in and external fields, such multiscale approaches enable researchers to embed ferroic orders across all components within a battery. Specifically, the advancement of chemically robust multiferroic chemistries and heterostructures, such as HfO_2_‐based and layered/VDW multiferroics, polar metals, twin‐rich ferroelastics, can sustain strong, switchable *P*‐*M*‐*ε* coupling under high voltage, reductive, and solid‐state conditions. Together with *operando* fields, they can synergistically guide ion flux, metal nucleation, and reaction pathways, directly addressing transport limitations, interfacial mismatch, and failure in thick, high‐loading electrodes. This methodological framework rigorously exploits multiferroics’ scientific and engineering potential, paving a novel way for next‐generation battery design.
4. Data‐driven multiferroic battery platforms: A practical multiferroic battery platform requires a unified computational‐experimental framework that integrates high‐throughput density functional theory (HT‐DFT), machine‐learning potentials (MLPs), multiscale/multiphysics modeling, and *operando* data analytics. HT‐DFT can rapidly screen multiferroic materials databases for battery‐relevant descriptors, such as spontaneous polarization, magnetic moment, switching barrier, elastic anisotropy, defect energetics, ion‐migration barrier, and interfacial stability, while MLPs extend these predictions to realistic spatiotemporal scales by resolving domain evolution, space‐charge‐layer thickness, strain thresholds, and defect‐coupled ferroic responses in complex battery environments. When coupled with continuum models and experimental feedback under programmable external stimuli, these descriptors can be translated into quantitative predictions of ASR, CCD, interphase evolution, reaction selectivity, and mechanical reliability. This closed‐loop, data‐driven workflow will allow multiferroicity to be screened, encoded, and dynamically regulated in practical cells, establishing a mechanistically grounded route from lab‐scale discovery to scalable multiferroic‐enabled battery technologies.
5. Scalable and manufacturing‐compatible embodiment of multiferroic functionality: A decisive next step is to translate multiferroic functions from epitaxial, single‐crystal, or delicately defect‐engineered model systems into battery‐relevant architectures that are compatible with high‐throughput manufacturing. This requires process‐tolerant embodiments of ferroic responses in polycrystalline ceramics, composite interphases, textured particles, printable heterostructures, and field‐ or stress‐imprinted metastable states that survive slurry casting, calendering, co‐sintering, and long‐term cycling. The central metric should therefore shift from maximizing ideal ferroic order to defining the minimum effective P‐M‐ε response needed to generate measurable gains in ASR, CCD, interphase stability, or fracture resistance under practical cell conditions. Coupling such materials development with techno‐economic analysis, thermal‐budget minimization, precursor selection, and line‐compatibility assessment will be essential for establishing multiferroicity as a scalable and cost‐effective design variable in commercial batteries.

## Author Contributions

**Jiaqi Su**: conceptualization, formal analysis, writing – review and editing, writing – original draft, visualization, methodology, validation, software, project administration, data curation.

**Yanda Zhu**: conceptualization, visualization, formal analysis, writing – review and editing.

**Hao Peng**: writing – review and editing, visualization.

**Manman Li**: visualization, writing – review and editing, investigation.

**Xuzihan Zhang**: writing – review and editing, visualization, investigation.

**Meixiao Wu**: investigation, visualization, writing – review and editing, formal analysis, resources.

**Siyuan Zhang**: investigation, visualization, writing – review and editing.

**Yuhan Zeng**: investigation, visualization, writing – review and editing.

**Jiwen Liao**: investigation, visualization, writing – review and editing.

**Ming Luo**: investigation, visualization, writing – review and editing.

**Hetaishan Huang**: investigation, visualization, writing – review and editing.

**Yutong Wang**: investigation, writing – review and editing.

**Sean Li**: conceptualization, writing – review and editing, supervision, funding acquisition.

**Wenxian Li**: conceptualization, writing – review and editing, supervision.

## Conflicts of Interest

The authors declare no conflicts of interest.

## Data Availability

Data sharing not applicable to this article as no datasets were generated or analysed during the current study.
